# Whole genome and RNA sequencing analyses for 254 Taiwanese hepatocellular carcinomas

**DOI:** 10.1186/s40364-023-00492-7

**Published:** 2023-07-04

**Authors:** Ya-Sian Chang, Siang-Jyun Tu, Hong-Da Chen, Chin-Chun Chung, Ming-Hon Hsu, Yu-Pao Chou, Ya-Ting Lee, Ju-Chen Yen, Long-Bin Jeng, Jan-Gowth Chang

**Affiliations:** 1 Center for Precision Medicine, China Medical University Hospital, 2 Yuh-Der Road, Taichung, 404 Taiwan; 2 Epigenome Research Center, China Medical University Hospital, 2 Yuh-Der Road, Taichung, 404 Taiwan; 3 Department of Laboratory Medicine, China Medical University Hospital, Taichung, Taiwan; 4 School of Medicine, China Medical University, Taichung, Taiwan; 5 Organ Transplantation Center, China Medical University Hospital, 2 Yuh-Der Road, Taichung, 404 Taiwan

**Keywords:** Whole genome sequencing, RNA sequencing, Taiwanese HCC, Alternative splicing, Immune checkpoint gene, Tumor microenvironment

## Abstract

**Background:**

Comprehensive and integrative analysis of hepatocellular carcinoma (HCC) is important. In this study, we explored Taiwanese HCCs using multi-omics analyses.

**Methods:**

We analyzed 254 HCCs by whole genome sequencing and total RNA sequencing, and then used bioinformatic tools to analyze genomic and transcriptomic alterations in coding and non-coding sequences to explore the clinical importance of each sequence.

**Results:**

The frequencies of the five most commonly mutated cancer-related genes were *TERT*, *TP53*, *CTNNB1*, *RB1*, and *ARID1A*. Genetic alteration frequencies influenced the etiology of HCC; some alterations were also correlated with clinicopathological conditions. Many cancer-related genes had copy number alterations (CNAs) and structure variants (SVs) that changed according to etiology and exhibited potential associations with survival. We also identified several alterations in histone-related genes, HCC-related long non-coding RNAs, and non-coding driver genes that may contribute to the onset and progression of HCC. Transcriptomic analysis revealed that 229 differentially expressed and 148 novel alternative splicing (AS) genes, as well as the presence of fusion genes, were associated with patient survival. Moreover, somatic mutations, CNAs, and SVs were associated with immune checkpoint gene expression and tumor microenvironment. Finally, we identified relationships among AS, immune checkpoint gene expression and tumor microenvironment.

**Conclusions:**

This study shows that genomic alterations are associated with survival, including DNA-based and RNA-based data. Moreover, genomic alterations and their associations with immune checkpoint genes and the tumor microenvironment may provide novel insights for the diagnosis and treatment of HCC.

**Supplementary Information:**

The online version contains supplementary material available at 10.1186/s40364-023-00492-7.

## Background

Liver cancer, of which hepatocellular carcinoma (HCC) is the predominant form, is the fifth most common cancer and the second most common cause of cancer-related death worldwide [1]. In addition to genetic factors, the onset and progression of HCC are associated with chronic viral infection, alcohol abuse, diabetes mellitus, obesity, metabolic diseases, hemochromatosis, and autoimmune hepatitis [2, 3]. These conditions induce liver injury and progressive inflammation; liver cells eventually exhibit necrosis, regeneration, somatic mutations, and chromosomal instability [4–7]. Recent studies have revealed many genomic alterations in HCCs, along with numerous frequently altered genes (e.g., *TP53*, *CTNNB1*, and *TERT*) [4, 8–18]. Despite many potential therapeutic targets, few drugs have demonstrated promising effects in clinical settings; most of the drugs only increased survival by a few months, indicating the need for novel treatment options for HCC [3, 19, 20].

Since 1984, the Taiwanese government has introduced a series of national initiatives to prevent hepatitis B virus (HBV)-related HCC through universal HBV vaccination. These efforts have resulted in a reduced incidence of HBV-related HCC in the young population [21]. Chinese herbal prescriptions for diseases including chronic hepatitis are also part of the national health system [22]. We have hypothesized that, in Taiwan, HCC is associated with interactions among viral infections, economic boom-related overconsumption of food and alcohol, and Chinese herbal prescriptions for chronic diseases [21–23]. Because of these complex interactions, the genomic landscape of HCC in Taiwan may differ from the landscapes in other areas, where it is mainly associated with HBV, hepatitis C virus (HCV), non-alcoholic fatty liver disease, and alcoholic liver disease. Comprehensive and multi-omics analyses of HCC in Taiwan are lacking. In this study, we used whole genome sequencing (WGS), total RNA sequencing (RNA-seq), and clinical data to explore the complex interactions contributing to HCC onset and progression in Taiwan.

## Methods

HCC was identified by pathological diagnosis. Tumor and adjacent non-tumor liver tissue samples were collected after surgical resection, then frozen at − 80 °C and stored at the Tissue Bank of China Medical University Hospital (CMUH). all specimens were obtained from patients with appropriate consent from the relevant Institutional Review Board (Ethics Committee of CMUH, Protocol No.: CMUH109-REC3-055). DNA and RNA were collected from samples using the Mini (Qiagen) and NucleoSpin (Macherey-Nagel) kit, respectively. We used commercial technology for WGS and RNA-seq. Data were analyzed using the Illumina DRAGEN Bio-IT Platform. Detailed methods are presented in the supplementary material.

## Results

### Clinical data

The demographic data of the 254 HCC patients are presented in Table S1. We found that treatment received by HCC patients were associated with patient survival, as shown in Fig. S1.

### Somatic mutations

**Fig. 1:**
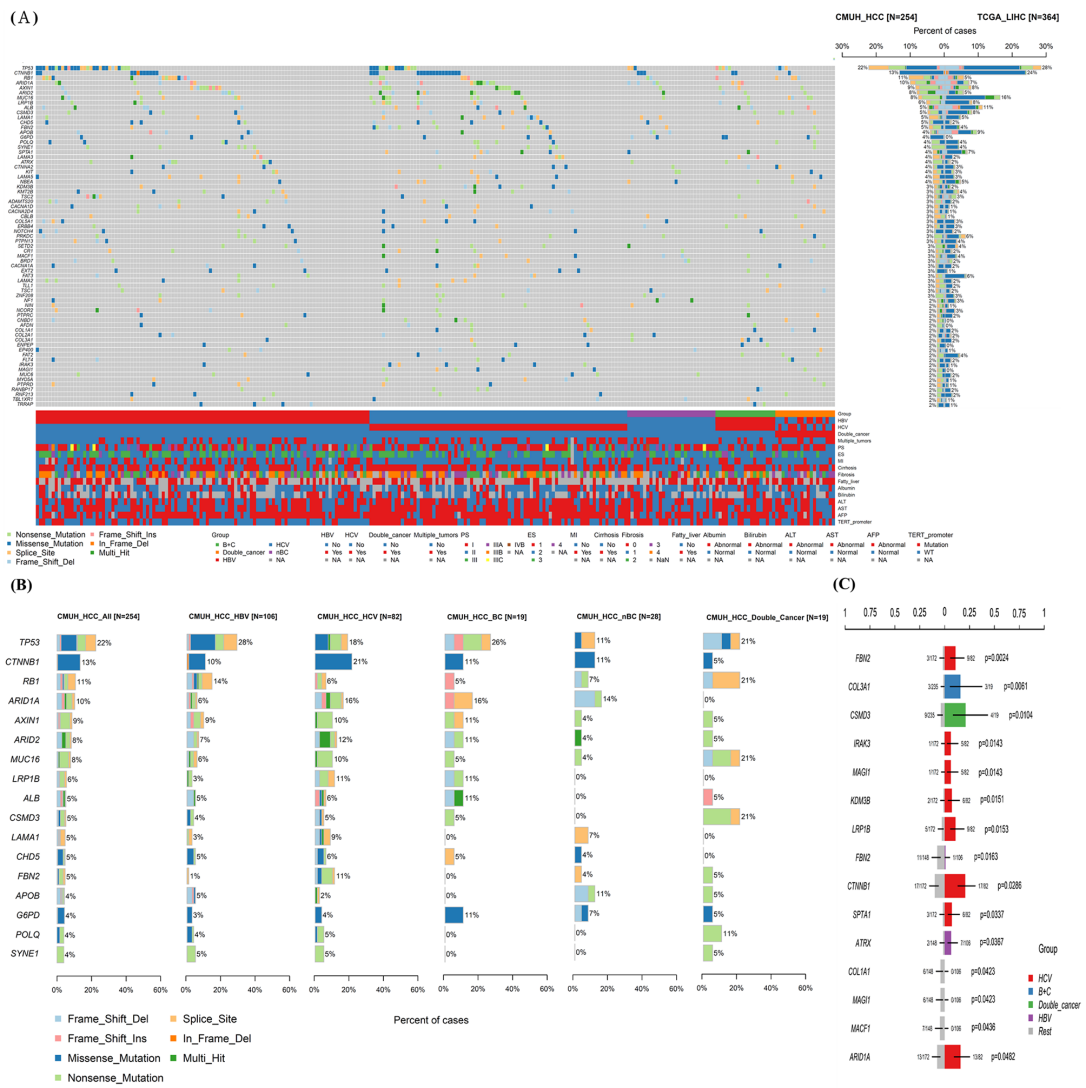
The cancer-related genes mutation landscape of Taiwanese HCCs. (**A**) Top panel shows individual tumor mutation burden. Middle panel shows genes with mutation frequencies > 2%, as well as mutated genes (left column) and comparison with TCGA-LIHC (right column); clinical data are shown at bottom. Bottom panel shows details of HCC classification into five subgroups, along with clinicopathological results and *TERT* mutations, for 254 HCC patients. (**B**) Mutational frequencies of 17 commonly mutated genes and one germline mutation in five subgroups of HCCs. (**C**) Frequencies of mutated genes with statistically significant differences among subgroups

The mutational landscape is shown in Fig. 1. The top 10 most common mutated genes were *TP53* (22% vs. 28%), *CTNNB1* (13% vs. 24%), *MUC16* (8% vs. 16%), *LRP1B* (6% vs. 8%), *ALB* (5% vs. 11%), and *CSMD3* (5% vs. 8%) and the overall mutation rate was lower than the rate reported in The Cancer Genome Atlas-Liver Hepatocellular Carcinoma (TCGA-LIHC). Some mutated genes were present at higher frequencies, such as *RB1* (11% vs. 5%), *ARID1A* (10% vs. 7%), *AXIN1* (9% vs. 8%), and *ARID2* (8% vs. 5%), compared with TCGA-LIHC; the *TERT* mutation rate (47.24%) was determined by analysis of promoter (Fig. 1A). Figure 1B shows the most commonly mutated genes and gene mutation frequencies among the various etiologies of Taiwanese HCCs.

We also compared the mutational frequencies of Taiwanese HCCs with the frequencies in various subgroups in TCGA; we found that the frequencies of cancer-related genes were different from the rates in other regions, even in Asia (Fig. S2).

Analysis of mutational frequencies according to etiology showed that *FBN2*, *IRAK3*, *MAGI1*, *KDM3B*, *LRP1B*, *CTNNB1*, *SPTA1*, and *ARID1A* were more frequently mutated in HCV-related HCC (p = 0.0024, 0.0143, 0.0143, 0.0151, 0.0153, 0.0286, 0.0337, 0.0482, respectively), *ATRX* was more frequently mutated in HBV-related HCC (p = 0.0367), *COL3A1* was more frequently mutated in dual HBV/HCV inflection (p = 0.0061), and *CSMD3* was more frequently mutated in double cancer-related HCC (p = 0.0104) (Fig. 1C).

Analysis of associations between clinical data and genomic alterations showed that more women had HCV infection (p = 0.0127), and HCV-related HCC was associated with a higher rate of cirrhosis (p = 2.961e-05). Kaplan-Meier survival analysis indicated that the different etiologies did not influence survival. (Fig. S3). *TERT* promoter mutations frequently occurred in HCV-related HCC (p = 5.024e-06) and were uncommon in HBV-related HCC (p = 2.812e-06). Moreover, the presence of *TERT* promoter mutation was associated with a significant decrease in overall survival (p = 0.039) (Fig. S4).

### Copy number alterations (CNAs)

CNAs, which involved chromosomal regions and related genes, are shown in the heatmap (Fig. S5A) and circos plot (Fig. S6A). The most frequent alterations in chromosomal arm were copy number gains in 1q, 6p, 7, 8q, and 17q, as well as copy number losses in 4q, 8p, 13p, 16, and 17p; whole chromosomal amplification did not affect patient survival (p = 0.78). Loss of 17p was associated with better survival (p = 0.05), whereas 7q amplification was associated with worse survival (p = 0.053) (Fig. S5B). Subsequent analyses of cancer-related genes in these regions of chromosomal alteration revealed that they may contain several oncogenes and tumor suppressor genes (TSGs), resulting in simultaneous gain or loss of these genes (i.e., overexpression or down-expression of both oncogene and TSG). For example, deletion of TSGs (*CHD3*, *GPS2*, *PER1*, and *YWHAE* in the17p deletion) [24, 25] was associated with better survival. Survival-related CNA-involved genes are shown in Fig. S5C and Fig. S6B. Associations between HCC etiology and CNAs of related genes are shown in Fig. S6C. Overall, double cancers and HBV-related HCCs had more copy number gains, compared with other etiologies.

### Structural alterations

Analysis of cancer driver structural variants **(**SVs) revealed 17,639 somatic SVs in our cohort (Fig. S7). We used probability of being loss-of-function intolerant (pLI) to analyze somatic SVs; we identified 1108 (p < 2.2e-16, odds ratio = 1.69) and 1761 (p < 2.2e-16, odds ratio = 0.60) involved oncogenes or TSGs, respectively [24–27], for somatic SVs in our HCC cohort (Fig. S8).

**Fig. 2:**
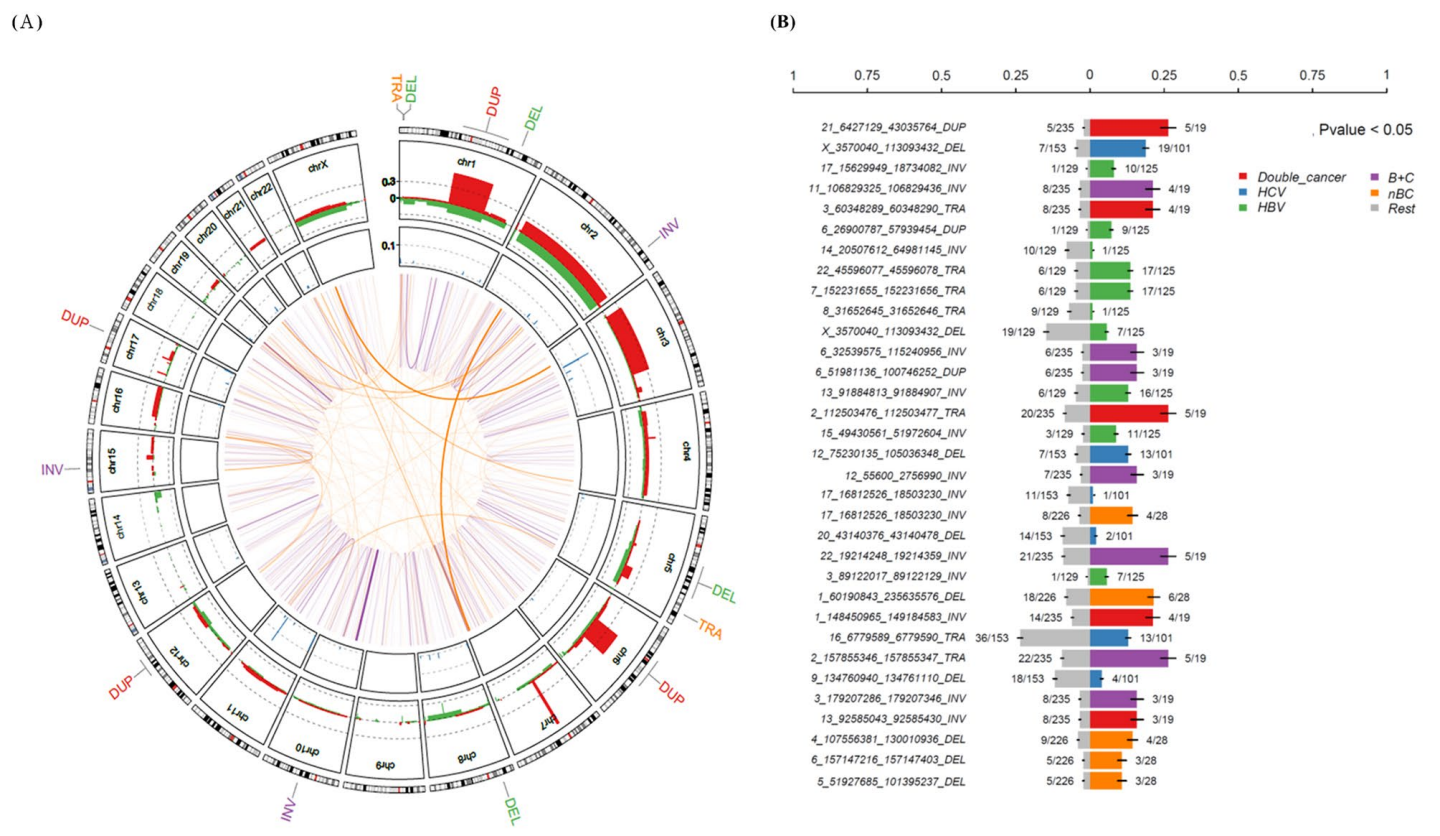
The landscape of somatic structural variants (SVs) in 254 Taiwanese HCCs. (**A**) Circos plot of somatic SVs. Outer: 13 survival-related somatic SVs; middle: chromosomal location of SVs; inner: details of SV-involved chromosome(s). (**B**) Frequencies of somatic SVs with statistically significant differences among etiologies

We then analyzed the clinical significance of somatic SVs with > 3% recurrent frequencies; associations between HCC etiology and SVs are shown in Fig. 2. Most SVs were on the same chromosome, and only a few SVs involved different chromosomes. Thirteen SVs (all associated with patient survival) were in the outer areas of the circos plot of chromosome (Fig. 2A). Five SVs (one duplication, two translocations, and two inversions) were associated with double cancers, two deletion SVs were associated with HCV-related HCC; seven SVs (four inversions, two translocations, and one duplication) were associated with HBV-related HCC, seven SVs (five inversions, one duplication, and one translocation) were associated with dual HBV/HCV infection HCC; and five SVs (one inversion and four deletions) were associated with non-HBV/non-HCV-related HCC (Fig. 2B). We identified 13 somatic SVs associated with patient survival (Fig. S9); 7 of the 13 somatic SVs were novel (Fig. S10).

### Mutation analysis of 114 histone-related genes, 74 HCC-related long non coding RNAs (lncRNAs), and 36 non-coding driver genes

Among 114 histone-related genes, we identified 39 variants with ClinVar pathogenic/likely pathogenic (P/LP; one case) or CADD ≥ 30 (21 cases); *HILS1* (4 cases) had the highest mutated rate (Table S2). We validated *HILS1* mutations using Sanger sequencing (Fig. S11). Among 74 HCC-related lncRNAs, we identified 24 possible driver variants in 254 HCCs; *HAGLR* and *LINC473* (5 cases) had more mutations (Table S3). Among 36 non-coding driver genes, we identified two *RMRP* promoter variants with ClinVar P/LP and one *COX6B2* variant with CADD ≥ 30. Many *NEAT1* and *Malat1* variants were detected, and their CADD scores were null; further analyses are needed to identify the roles of these variants (Table S4).

### Mutational signatures

**Fig. 3:**
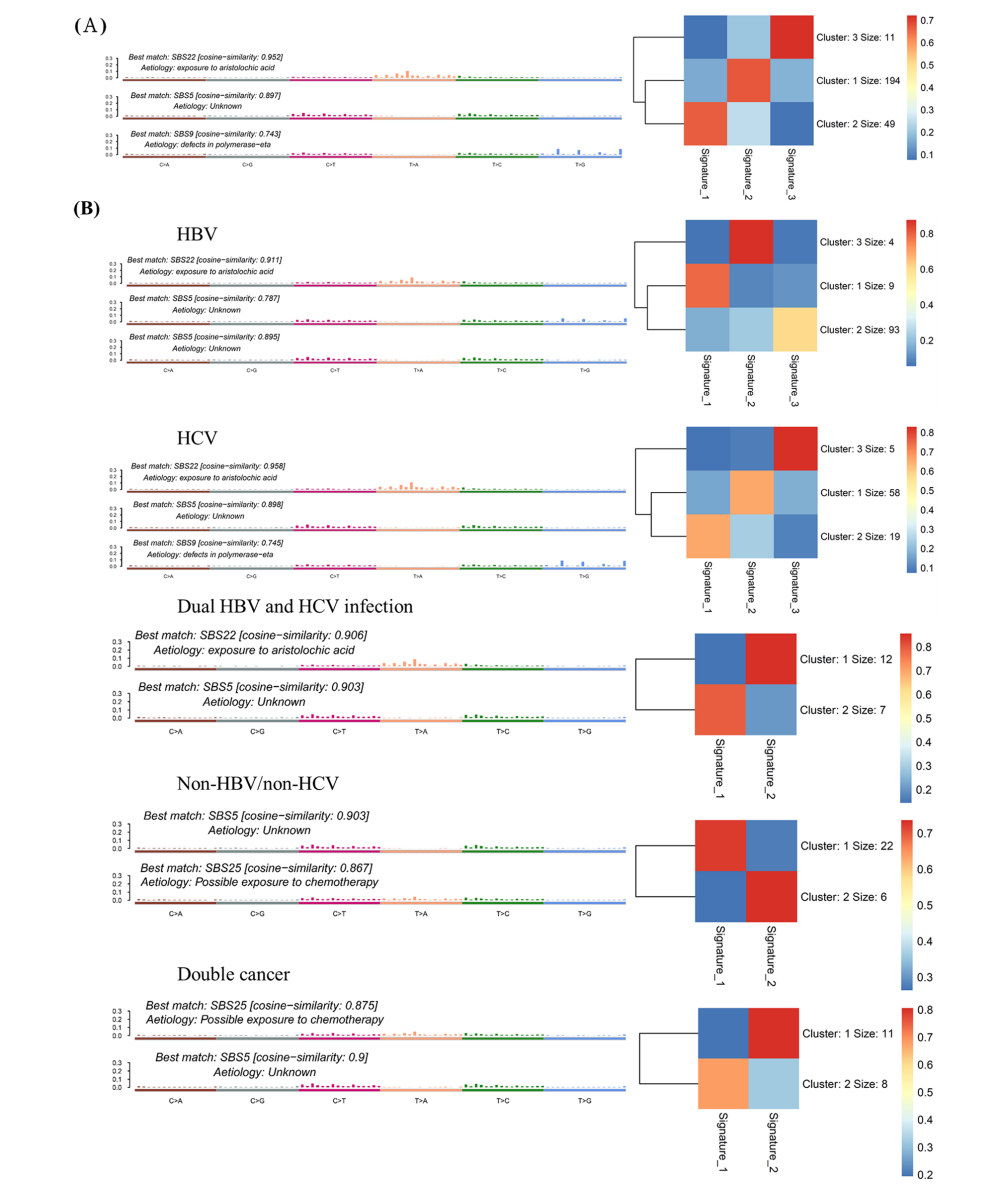
Mutational signature on 254 Taiwanese HCCs. (**A**) Three signatures were extracted from 254 Taiwanese HCC and compared to COSMIC signatures catalogs, and the signature 1, 2 and 3 were correlated with SBS22 (aristolochic acid exposure), SBS5 and SBS9 respectively. Cluster with a cosine similarity of Taiwanese HCC resulting in three signatures and related case number. (**B**) Mutational signature and subgroup of HCCs: HBV infection, HCV infection, dual HBV and HCV infection, non-HBV/non-HCV, and double cancers

We performed mutational signature analysis on the core set of 254 HCCs by non-negative matrix factorization. There were three types of mutational signatures in Taiwanese HCCs. The case numbers and distributions of mutational signatures in each cluster are shown in Fig. 3A. Associations among viral type, biochemical data, and *TERT* mutation are shown in Fig. S12. Signature 1 (19.29%, 49 cases) was matched to SBS22 and significantly associated with the plant-derived carcinogen aristolochic acid (AA) signature, with a predominance of A:T-to-T:A transversions at T/CAG tri-nucleotide motifs. Signature 2 (194 cases) was matched to SBS5 with unknown etiology. Signature 3 was matched to SBS9 (11 cases) and associated with polymerase eta.

Greater proportions of HCV and dual HBV/HCV infection-related HCCs exhibited the AA exposure signature (19/82 and 7/19, respectively); this signature was present in 9/106, 0/28, and 0/19 of HBV, non-HBV/non-HCV, and double cancer-related HCC, respectively. Among cases of non-HBV/non-C and double cancer-related HCCs, 6/28 and 8/19, respectively, exhibited the chemotherapy-related signature SBS25; no cases with other etiologies exhibited SBS25 (Fig. 3B).

### Differential expression genes (DEGs) and clinical significance

In total, 3585 of 51,497 genes were differentially expressed: 2694 genes were overexpression, and 891 genes were down-expression (**details can be provided on request**). Next, we analyzed the DEGs and their associations with survival; 229 genes (123 protein-coding and 106 non-coding) were associated with survival. Among the 123 protein-coding genes, increased expression levels of 28 genes were associated with better survival, whereas increased expression levels of the remaining 95 genes were correlated with worse survival. Among the 106 non-coding genes, increased expression levels of 33 genes (including 16 novel genes) and the remaining 73 genes (including 29 novel genes) were associated with better or worse survival, respectively (Table S5).

We conducted an additional analysis to investigate the correlation between various etiologies and the expression levels of 229 genes. Our finding revealed that *ASB15*, *CYP17A1*, *SLC5A10*, and *ADRA2B* exhibited low expression levels in cases with viral etiology, while *TFPI2*, *IGF2BP3*, *ANKS1B*, and *AC132807.2* demonstrated higher expression levels in cases with viral etiology (p < 0.05) (Table S6A).

We also compared the expression levels of 229 genes between genders and identified five genes (*IGF2BP3*, *PKIA*, *ANKS1B*, *AL049734.2*, and *AC072028.1*) that were significantly overexpressed in females (p < 0.001) (Table S6B).

### Fusion gene analysis and clinical significance

After low confidence fusion events had been filtered out, 206 fusion events from 180 fusion genes remained (Table S7); 88 cases exhibited fusion genes, including 19 cases with > 2 fusion genes. Three fusions (*SLC45A2-AMACR*, *ITCH-ASIP*, and *RNF138-RNF125*) were present in > 1 case (Fig. S13A). Compared with HCC patients who lacked fusion events, HCC patients with fusion events had better prognoses (Fig. S13B).

Fifty of fusion genes have been reported and 14 involving known cancer genes (*ALDH2-ACAD10*, *BIRC6-SPAST*, *BIRC6-TTC27*, *CGNL1-TCF12*, *CPEB3-IDE*, *CTNNA1-KDM3B*, *DNAJB1-PRKACA*, *LRP5-CHKA*, *PPFIBP1-STK38L*, *RNF213-SLC26A11*, *RXRA-WDR5*, *SEC16A-NOTCH1*, *WNK1-ERC1*, and *ACVR1B-ACVRL1*). Among the 111 novel fusion genes, 18 involving known cancer genes (*APOH-NKTR*, *ARHGEF10L-HEG1*, *C1S-GNAI2*, *CCND1-FGF19*, *CHP1-INO80*, *CPB2-LCP1*, *KANSL1-AC005324.3*, *LASP1-NCOR1*, *LPP-RNF139*, *PIK3R1-NKD2*, *QKI-CDC45*, *RHOA-MST1*, *RNF139-LPP*, *RXRA-RAPGEF1*, *SLC30A7-DPYD*, *SRSF3-PNPLA1*, *TCF12-TMOD3*, and *UBL3-SYNE1*) (Table S8).

### Analysis of HBV DNA and transcripts using WGS and RNA-seq

**Fig. 4:**
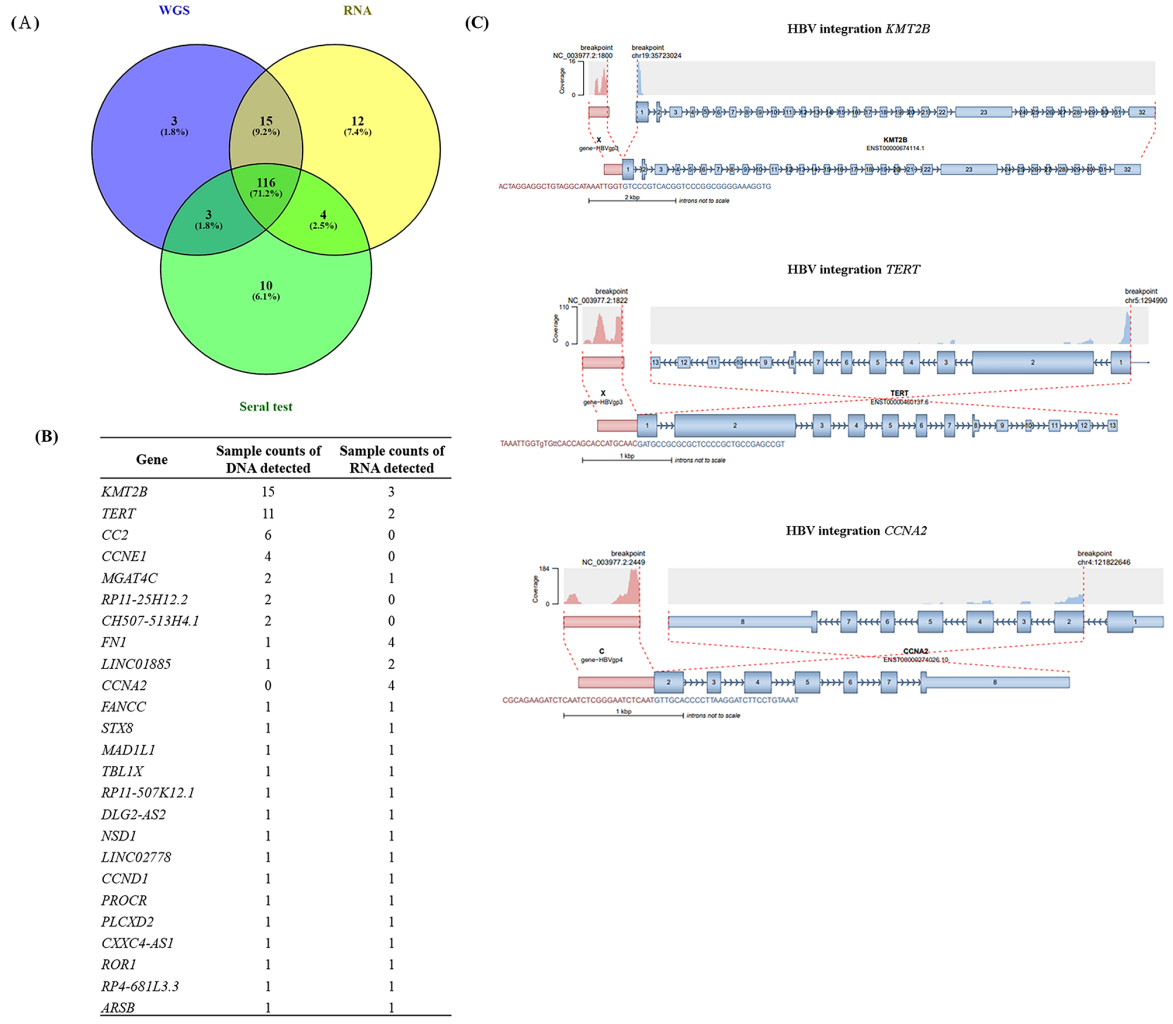
HBV integration analysis for 254 Taiwanese HCCs. (**A**) Association among results of HBV serum testing, WGS, and RNA-seq. (**B**) Association between results of WGS and RNA-seq for 25 HBV integrated genes. (**C**) Junction of representative fusion genes

We used kraken tools to analyze HBV DNAs and HBV-related transcripts. In total, 116 HBV-related HCCs were closely associated with the results of serum testing, RNA-seq, and WGS; 131 cases were closely associated with the results of WGS and RNA-seq; and 3 and 12 cases were closely associated with only WGS or only RNA-seq, respectively (Fig. 4A).

Among HBV-related fusion genes, we selected fusion detected by WGS or RNA-seq that exhibited > 2 reads. Twenty-five genes were fused with HBV virus; the top three fusion genes were *KMT2B*, *TERT*, and *CC2*. Only a few DNA fusion genes had RNA transcripts (Fig. 4B); the structures of representative fusion genes are shown (Fig. 4C). We compared the survival of HCCs with and without fusions, we found that HBV-related fusion gens were not associated with patient survival (p = 0.86 using RNA analysis, p = 0.15 using DNA analysis) (Fig. S14).

### Alternative splicing (AS) analysis and clinical significance

We used SUPPA2 to estimate the abundances of AS events and calculate proportion spliced-in (PSI) values for Taiwanese HCCs. There were 914 AS events for 0.2 PSI and 920 events for 0.3 PSI. For both 0.2 and 0.3 PSI, the number of common events was 345 (Fig. S15). We then analyzed survival-related AS events; 98 events in 93 genes and 86 events in 75 genes were associated with better or worse survival, respectively (Fig. S16).

For the novel AS genes, 148 have not been previously reported (Table S9). Increased expression level of 79 genes (79/93) that underwent AS were associated with better survival. However, increased expression levels of 65 other genes (65/75) that underwent AS were associated with worse survival. AS for *CDK13*, *CFLAR*, *EGLN1*, and *ZNF717* exhibited aberrant effects on survival that were caused by different types of AS event (Fig. S17); for example, *CDK13* alternative first exon was associated with better survival, whereas *CDK13* alternative last exon was associated with worse survival.

**Fig. 5:**
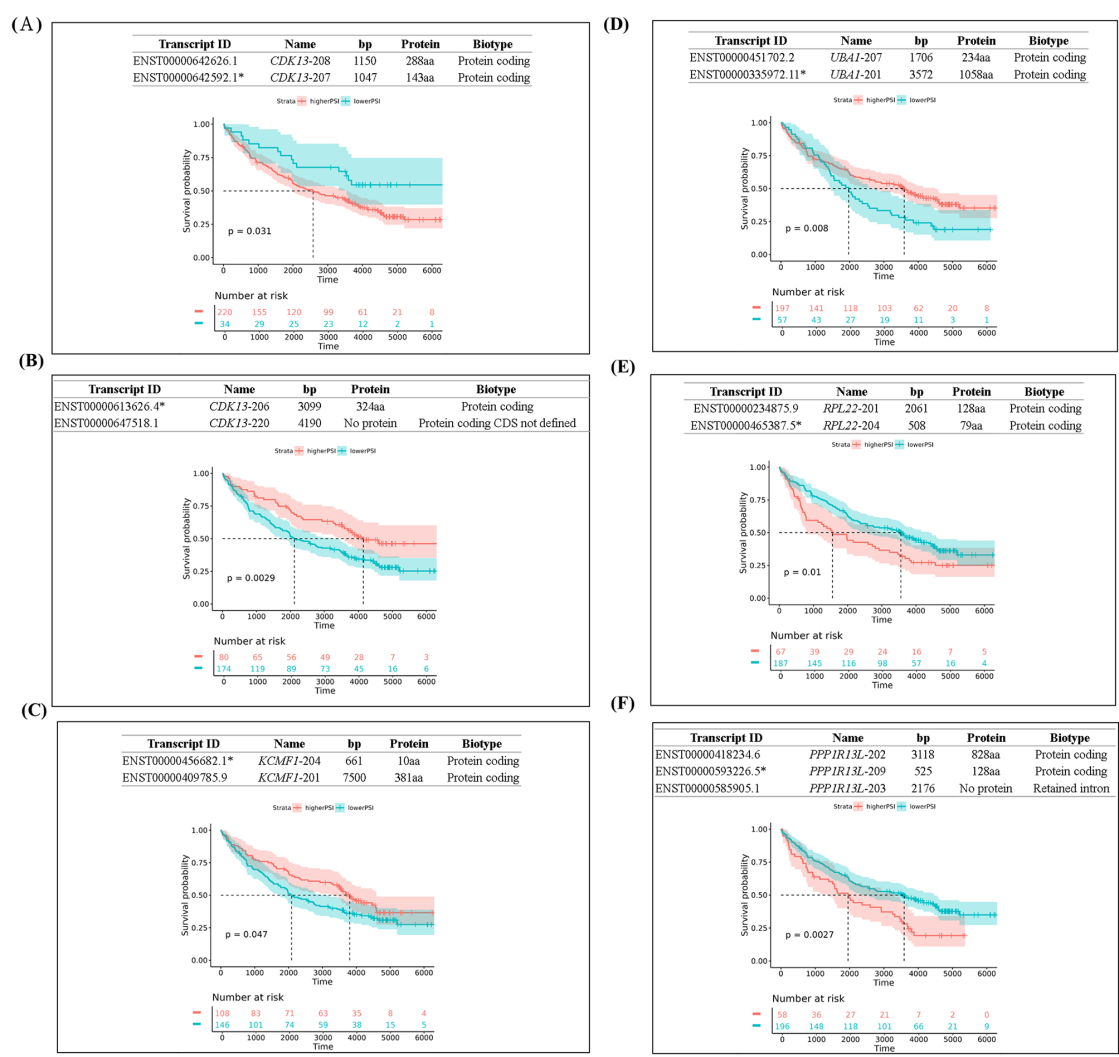
Representative examples of the clinical significance of alterations in protein-coding genes with survival-related alternative splicing (AS) event. (**A**-**B**) *CDK13* (**C**) *KCMF1* (**D**) *UBA1* (**E**) *RPL22* (**F**) *PPP1R13L* *: survival-related AS event

We analysis the impacts of AS on protein structure; different AS forms of the same gene led to changes in survival (Fig. 5A-B). AS of oncogene-like genes [28, 29] resulting in loss of function (e.g., frameshift with nonsense decay or protein truncation) was associated with better survival (Fig. 5C-D and Fig. S18). With respect to AS of TSG-like genes [24, 30]with worse survival, short truncated proteins were identified by analysis of possible coded protein from AS transcripts (Fig. 5E-F and Fig. S19).

### Associations between immune checkpoint gene expression and genomic alterations

We analyzed the effects of genomic alterations on the expression patterns of 42 immune checkpoint genes. Eight highly recurrent mutated genes (*TP53*, *CTNNB1*, *RB1, ARID1A, AXIN1, ARID2, MUC16*, and *TERT*) were selected for analysis (**Details can be provided on request**). Only mutations in *ARID1A* was associated with higher expression of *CD70* (Fig. S20A).

Recurrent copy changes in many cancer-related genes also affected the expression patterns of immune checkpoint gene (**Details can be provided on request**). Statistically significant copy changes occurred in *AKT1* (influencing *TNFSF14*), *AKT2* (influencing *CD40LG*), *ARID1A* (influencing *CD276*), *ARID1B* (influencing *CD40LG* and *TNFRSF4*), *AXIN1* (influencing *CD276*, *CD96*, and *PDCD1LG2*), *GNAS* (influencing *TNFSF14* and *PDCD1LG2*), *IDH2* (influencing *CD274* and *CD96*), *NF1* (influencing *CD40LG*), *SMARC4* (influencing *HHLA2*, *TNFSF9*, and *VTCN1*), *STAT3* (influencing *CD40LG*, *LGALS3*, and *CD96*) and *TSC2* (influencing *CD276* and *TRAC*) (Fig. S20B-V).

### Associations of 64 immune and stroma cell types/three scores with various genomic alterations

We used xCell to explore associations between genetic alterations and the tumor microenvironment. For mutation association analysis, we selected the top eight mutated genes (described above) to explore their associations with the numbers of immune and stroma cell. We found that *CTNNB1* mutations were associated with a decrease in epithelial cells but increases in hematopoietic stem cells and megakaryocyte-erythroid progenitor (MEP). *RB1* mutations and copy loss were associated with decreases in adipocytes, lymphatic (ly) endothelial cells, stroma score, and preadipocytes; they were associated with increases in common lymphoid progenitor (CLP), pro B-cells, and Th1 and Th2 cells. *TERT* mutations were associated with a decrease in neurons, whereas *ARID1B* copy loss was associated with an increase in microvascular (mv) endothelial cells. *IL6ST* copy loss was associated with a decrease in microenvironment score. *MDM4* amplification was associated with a decrease in pro B-cells, whereas *TSC1* amplification was associated with a decrease in adipocytes. *TSC1* copy loss was associated with an increase in sebocytes. *AXIN1*, *BRD7*, *IDH2* and *TSC2* copy loss were each associated with a decrease in hepatocytes (Fig. S21).

With respect to 17p deletion and 7q duplication, none remained statistically significant after adjustment. The SVs (AnnotSV_ID: 1_105963115_182721369_DUP_1) involving 38 protein genes was associated with basophils (Fig. S22).

**Associations among the expression of immune checkpoint genes, the number of 64 cell types/3 scores, and AS**.

**Fig. 6:**
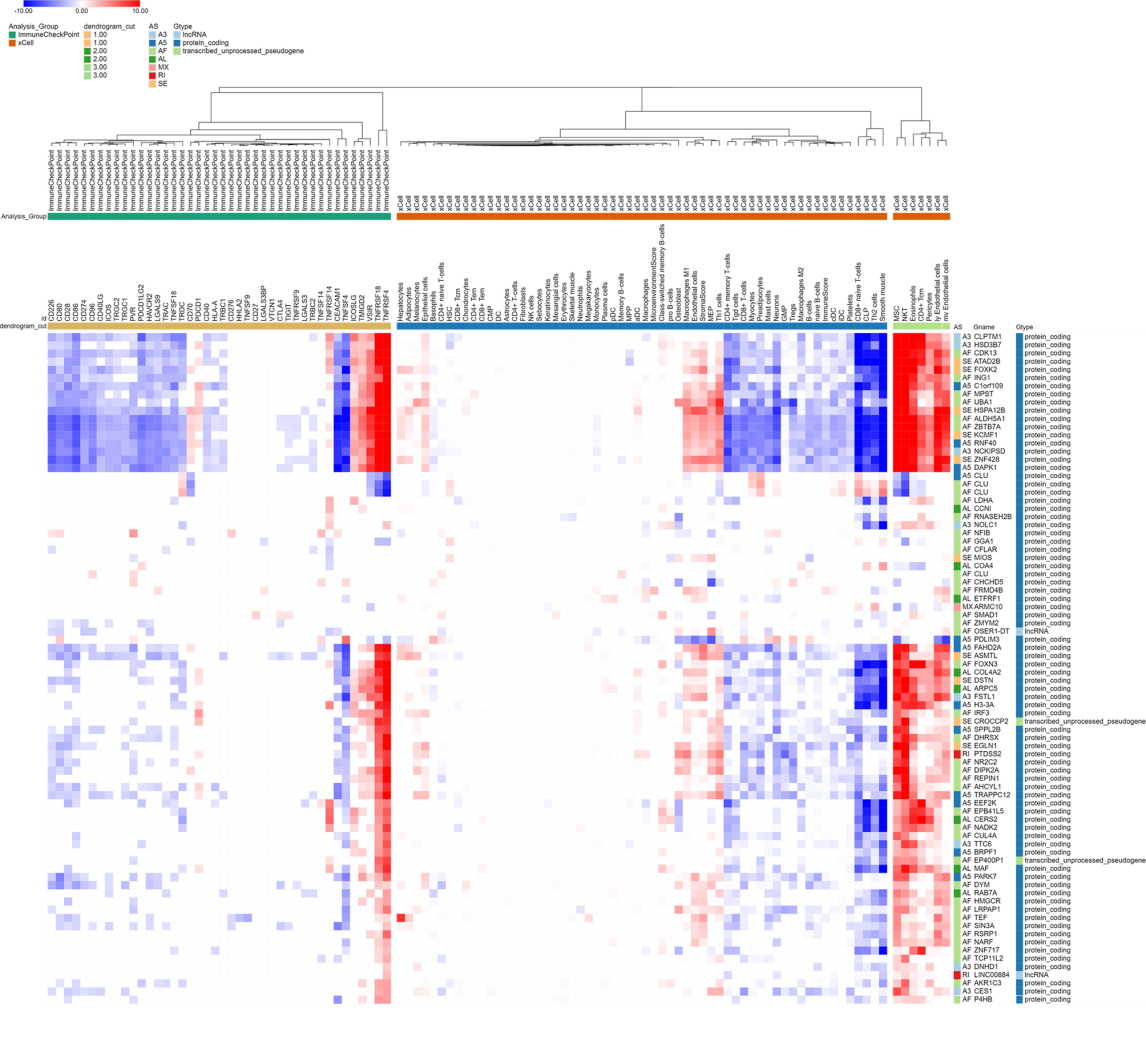
Heatmap of associations between better survival-related AS events and expression patterns of immune checkpoint genes, as well as numbers of immune and stroma cell types and corresponding scores. Heatmap illustrates significant differences between higher and lower PSI groups with respect to better survival-related AS events. Red indicates that median cell score or gene expression is higher in the higher PSI group than in the lower PSI group, whereas blue indicates the opposite relationship. Color depth indicates adjusted p-value, such that deeper colors representing lower p-values

We explored associations among survival-related AS events, immune checkpoint genes, and 64 cell types/3 scores. AS events in well-known cancer-related genes involving more cancers usually have greater influence on both the numbers of immune and stromal cells and the expression patterns of immune checkpoint genes. For example, better survival-related AS of 17 well-known cancer-related genes (1st to 17th ) was associated with larger changes in the numbers of mesenchymal stem cells (MSC), natural killer T (NKT), eosinophils, central memory CD4 + T cell (CD4 + Tcm), pericytes, ly endothelial cells, mv endothelial cells, Th1 cells, MEP, endothelial cells, and inflammatory (M1) macrophages, as well as greater changes in stroma score; it was associated with smaller changes in the numbers of CD8 + naïve T-cells, CLP, Th2 cells, and smooth muscle. This AS was also associated with significant increases in the expression levels of the immune checkpoint genes *TNFRSF4*, *TNFRSF18*, *VSIR*, *TMIGD2*, *ICOSLG*, *TNFRSF14*, *PDCD1*, and *CD70*, along with decreases in the expression levels of *TNFSF4*, *CEACAM1*, *PDCD1LG2*, and *CD86*; AS of other genes showed similar associations (Fig. 6). Fig. S23 shows that worse survival-related AS events were associated with distinct changes in cell type and immune checkpoint gene expression.

## Discussion

In this study, we used WGS to analyze a large number of HCC cases and confirm the uniqueness of Taiwanese HCCs implied in our previous study [31, 32]. We identified different frequencies of cancer-related gene mutations; new SVs, AS genes, and fusions; and a high frequency of CNAs that differed from the frequencies in other areas [8–18]. We also analyzed mutations in 74 HCC-related lncRNAs, which revealed 26 cases (10.24%) of driver variants in 254 HCCs; *LINC473* and *HAGLR* had a higher mutation rate, as indicated by highly stringent selection. Three cases had non-coding driver gene mutations. We also found that 120 cases had *TERT* promoter mutations. These non-coding genes may play a role in the onset and progression of Taiwanese HCCs.

Recently, the roles of histone gene mutations in cancer onset and progression have been clarified [33]; however, most studies of HCC have been focused on alterations in histone modification enzymes and expression of histone variants [34], rather than mutations in histone structural genes. Here, we analyzed mutations in 114 histone-related genes among Taiwanese HCCs; 8.66% (22/254) had mutations, and *HILS1* had a higher mutated rate, as indicated by highly stringent selection. Thus, we suspect that histone mutations contribute to the onset and progression of HCC [33, 34].

Each case of HCC usually had many alterations of large CNAs; these CNAs may contain several genes (e.g., oncogenes and TSGs), resulting in simultaneous gain or loss of these genes. For example, deletion of 17p includes *AURKB*, *CHD3* (TSG), *GAS7*, *GPS2* (TSG), *PER1* (TSG), *TP53*, *RABEP1*, *USP6* (oncogene), and *YWHAE* (TSG) resulting in both oncogene and TSG deletions that are associated with better survival. Our results indicate that the analysis of alterations (including bases and copy numbers) in each oncogene or TSG does not accurately assess the clinical impacts; direct evaluation of large CNAs is needed to improve the overall understanding of HCC onset and progression.

In SV analysis, we used pLI methods, Taiwan biobank and gnomAD databases, and non-tumor tissues and benign liver lesions to select somatic SVs. Using this approach, we identified many HCC-related somatic SVs; 13 unique SVs were correlated with patient survival.

We also analyzed fusion genes and found that they involve known cancer genes whether they are already reported fusions or novel fusions. Bayard et al., indicated that some HCC subgroups exhibit cyclin activation through various mechanisms, including HBV and adeno-associated virus type 2 insertions and enhancer hijacking and recurrent *CCNA2* fusions, defining a homogenous entity of aggressive HCC [35]. Although we did not reveal any *CCNA2* fusion in this study, we identified that HBV inserted into *CCNA2* and *CCNE1*.

Expression patterns of immune checkpoint genes in tumor cells have important role in evading attacks by host immune cells; mutations in cancer-related genes may influence these processes [36]. Our results showed that alterations in these genes influenced the expression patterns of immune checkpoint genes; for example, loss-of-function mutations in *ARID1A* led to high levels of *CD70* expression.

Diversity in the immune cell population and tumor microenvironment and associations with clinical outcomes have been identified in many studies [37–41]. Most studies have focused on point mutations, CNAs, or the expression patterns of immune genes and the numbers of immune cells; comprehensive and integrative analyses of distinct genomic alterations (e.g., somatic mutations, CNAs, SVs, fusions, and AS) within the tumor microenvironment have been limited. In this study, we explored their associations in 254 Taiwanese HCCs; we found that genomic alterations, CNAs, and SV influenced the tumor microenvironment. For example, *RB1* mutations and copy loss were associated with decreases in adipocytes, ly endothelial cells, stromal score, and preadipocytes; they were associated with increases in CLP, pro B-cells, and Th1 and Th2 cells. The SV (AnnotSV_ID: 1_105963115_182721369_DUP_1) was associated with a decrease in basophils. Accordingly, we suspect that genomic alterations have important roles in the HCC microenvironment [37].

In terms of the effects of survival-related AS events on immune cells and the expression patterns of immune checkpoint genes, AS of cancer-related gene had greater effects on immune and stroma cells, relative to AS without these characteristics; the numbers of immune and stroma cells were associated with both survival-related AS and the expression patterns of immune checkpoint genes. For example, AS that resulted in the loss of the *KCMF1* oncogene was associated with increases in the numbers of MSC, NKT, eosinophils, CD4 + Tcm, pericytes, and ly and mv endothelial cells; it was associated with decreases in the numbers of CD8 + naïve T-cells, CLP, Th2 cells, and smooth muscle; and it was associated with upregulation of *TNSRSF4*, *TNSRSF18*, and *VSIR*, as well as downregulation of *TNFSF4* and *CEACAM1*. Some AS patterns have already been associated with immune cell behavior; example associations include *NCKIPSD* (*SPIN90*) with stromal fibroblast activation in low stiffness stroma [42], *HSD3B7* with T cell-dependent plasma cell responses [43], *DAPK1* with NK cell ability and CD8 + T cell activation [44], and *HSPA12B* with tumor-associated endothelial cell activity and M2 polarization [45]. Based on these results, we suspect that survival-related AS events will be important in the evaluation and treatment of HCC; a combination of checkpoint blockade and AS modulating molecules may be more effective than an approach involving a single method. In our previous studies, we employed amiloride and its derivatives to modulate AS for the purpose of cancer treatment [46, 47].

Our study makes several significant contributions to the field of HCC research. Firstly, by conducting a comprehensive analysis of data that includes WGS and RNA-seq from a large number of HCCs, our study provides a more complete understanding of the genetic and transcriptomic landscape of HCC. Secondly, we explored the relationships among immune checkpoint gene expression, the tumor microenvironment, and AS, and our findings suggest that AS could be a promising approach for HCC treatment. Thirdly, we have reconfirmed the uniqueness of HCCs in Taiwanese patients.

A notable limitation of this study was that it used stringent criteria to select HCC-related genomic alterations, which may have resulted in the exclusion of some rare or weak effective alterations. Additionally, all cases were surgically treated, and we excluded patients who underwent transplantations as this may have resulted in different survival times compared to other studies. Finally, we used total RNA for transcriptome analysis, which may result in the loss of genes with low expression levels.

## Conclusions

In summary, we performed multi-omics analysis in HCC patients. CNAs, SVs, expression levels, alternative transcripts, and fusion transcripts could serve as potential biomarker for HCC. Overall, comprehensive and integrated approaches will provide a better understanding of the molecular basis of HCC and may facilitate the development of new therapeutic strategies.

## Electronic supplementary material

Below is the link to the electronic supplementary material.

Additional file 1: Figure S1. Survival curve indicating the effect of the HCC treatment approach on patient survival.

## Data Availability

The WGS and RNA-seq data for this study were submitted to the NCBI Sequence Read Archive under the BioProject PRJNA885992 and PRJNA870935.

## Abbreviations

HCC: Hepatocellular carcinoma
CNAs: Copy number alterations
SVs: Structure variants
AS: Alternative splicing
HBV: Hepatitis B virus
HCV: Hepatitis C virus
WGS: Whole genome sequencing
RNA-seq: RNA sequencing
TCGA-LIHC: The Cancer Genome Atlas-Liver Hepatocellular Carcinoma
TSGs: Tumor suppressor genes
pLI: Probability of being loss-of-function intolerant
lncRNAs: Long non coding RNAs
P/LP: Pathogenic/likely pathogenic
AA: Aristolochic acid
DEGs: Differential expression genes
PSI: Proportion spliced-in
MEP: Megakaryocyte-erythroid progenitor
ly: Lymphatic
CLP: Common lymphoid progenitor
mv: Microvascular
MSC: Mesenchymal stem cells
NKT: Natural killer T
CD4 + Tcm: Central memory CD4 + T cell
