# Supplementary material for: Whole genome and RNA sequencing analyses for 254 Taiwanese hepatocellular carcinomas
Source: Biomark Res. 2023 Jul 4;11:68. doi: 10.1186/s40364-023-00492-7 (PMC10320951; doi:10.1186/s40364-023-00492-7)
Supplement: Supplementary file 1 — Additional file 1: Figure S1. Survival curve indicating the effect of the HCC treatment approach on patient survival. [file 40364_2023_492_MOESM1_ESM.docx]

**Whole genome and RNA sequencing analyses for 254 Taiwanese hepatocellular carcinomas**

Ya-Sian Chang^1,2,3,4^, [Siang-Jyun Tu](https://pubmed.ncbi.nlm.nih.gov/?term=Tu+SJ&cauthor_id=34573440)^1,2,3^, Hong-Da Chen^1,2,3^, Chin-Chun Chung^1,2^, Ming-Hon Hsu^1,2,3^, Yu-Pao Chou^1,2,3^, Ya-Ting Lee^1,2^, Ju-Chen Yen^1,2^, [Long-Bin Jeng](https://pubmed.ncbi.nlm.nih.gov/?term=Jeng+LB&cauthor_id=36230503)^5^, Jan-Gowth Chang^1,2,3,4^

**Contents**

[**Supplementary methods** 3](#_Toc133049447)

[**Liver samples and clinical data** 3](#_Toc133049448)

[**DNA extraction and whole genome sequencing (WGS)** 3](#_Toc133049449)

[**Somatic variant calling** 4](#_Toc133049450)

[**Copy number variation** 5](#_Toc133049451)

[**Structure variant (SV) calling** 5](#_Toc133049452)

[**Mutational signature analysis** 6](#_Toc133049453)

[**RNA extraction and RNA sequencing (RNA-seq)** 6](#_Toc133049454)

[**RNA differential expression (DE) analysis** 7](#_Toc133049455)

[**Gene fusion analysis** 8](#_Toc133049456)

[**Metatranscriptomic analysis** 8](#_Toc133049457)

[**RNA alternative splicing (AS) analysis** 8](#_Toc133049458)

[**Cell score** 9](#_Toc133049459)

[**Statistical analyses** 9](#_Toc133049460)

[**Supplementary figures** 10](#_Toc133049461)

[**Fig. S1.** 10](#_Toc133049462)

[**Fig. S2.** 11](#_Toc133049463)

[**Fig. S3.** 12](#_Toc133049464)

[**Fig. S4.** 14](#_Toc133049465)

[**Fig. S5.** 16](#_Toc133049466)

[**Fig. S6.** 20](#_Toc133049467)

[**Fig. S7.** 23](#_Toc133049468)

[**Fig. S8.** 24](#_Toc133049469)

[**Fig. S9.** 25](#_Toc133049470)

[**Fig. S10.** 26](#_Toc133049471)

[**Fig. S11.** 27](#_Toc133049472)

[**Fig. S12.** 28](#_Toc133049473)

[**Fig. S13.** 29](#_Toc133049474)

[**Fig. S14.** 30](#_Toc133049475)

[**Fig. S15.** 31](#_Toc133049476)

[**Fig. S16.** 32](#_Toc133049477)

[**Fig. S17.** 33](#_Toc133049478)

[**Fig. S18.** 34](#_Toc133049479)

[**Fig. S19.** 35](#_Toc133049480)

[**Fig. S20.** 36](#_Toc133049481)

[**Fig. S21.** 37](#_Toc133049482)

[**Fig. S22.** 39](#_Toc133049483)

[**Fig. S23.** 40](#_Toc133049484)

[**Supplementary tables** 41](#_Toc133049485)

[**Table S1** 41](#_Toc133049486)

[**Table S2** 44](#_Toc133049487)

[**Table S3** 46](#_Toc133049488)

[**Table S4** 48](#_Toc133049489)

[**Table S5** 51](#_Toc133049490)

[**Table S6** 60](#_Toc133049491)

[**Table S7** 82](#_Toc133049492)

[**Table S8** 90](#_Toc133049493)

[**Table S9** 95](#_Toc133049494)

[**Supplementary results** 99](#_Toc133049495)

[**Clinical data** 99](#_Toc133049496)

[**References** 100](#_Toc133049497)

**Supplementary methods**

**Liver samples and clinical data**

Hepatocellular carcinoma (HCC) was identified by pathological diagnosis. Tumor and adjacent non-tumor liver tissue samples were collected after surgical resection, then frozen at −80 °C and stored at the Tissue Bank of China Medical University Hospital (CMUH). The Tissue Bank was established in 2005; has been accredited by the Taiwanese government since October 25, 2012; and contains > 20,000 cancer tissues of more than 20 types of cancers. We performed the Cancer Genome Sequencing project of CMUH [The Cancer Genome Atlas (TCGA) of CMUH], which was approved by the Ethics Committee of CMUH (CMUH110-REC3-221); this project sequenced > 1000 cases of different types of cancers from the Tissue Bank of CMUH. This HCC study was also approved by the Ethics Committee of CMUH (CMUH 109-REC3-055), and informed consent was obtained from all participants in accordance with the standard procedure of the CMUH Tissue Bank. Additionally, both the Declaration of Helsinki and the Good Clinical Practice Guidelines were followed.

**DNA extraction and whole genome sequencing (WGS)**

Frozen tissue DNA was extracted using a QIAamp® DNA mini kit (Qiagen, Heidelberg, Germany), in accordance with the manufacturer’s instructions. Barcoded DNA libraries were generated using a DNA PCR-Free Prep kit (Illumina, San Diego, CA, USA). Genomic DNA was diluted to 40 ng*/*μl using Resuspension Buffer (Illumina). Then, 25 μl of Bead-Linked Transposomes were used to fragment DNA; this was followed by the ligation of DNA fragments to barcoded adapters without polymerase chain reaction amplification. WGS libraries were sequenced on the NovaSeq 6000 instrument (Illumina) using 2x151-bp paired-end sequencing flow cells, in accordance with the manufacturer’s instructions.

**Somatic variant calling**

On average, > 400 Gb and 40–60× depth of mappable sequence data were obtained. After using Illumina Dragen Bio-IT Platform (v3.7) analysis of WGS data, we selected the variants of PASS on the FILTER column of VCF file, and data were analyzed in our bioinformatics pipeline. Variant frequencies were evaluated using the gnomAD (<https://gnomad.broadinstitute.org/>) and Taiwan biobank (TWB) (<https://www.twbiobank.org.tw/new_web/>) databases, as well as our database. Variant characteristics were analyzed using the ClinVar database (https://ftp.ncbi.nlm.nih.gov/pub/clinvar/vcf_GRCh38/) and combined annotation dependent depletion (CADD; https://cadd.gs.washington.edu/download). We collected pathogenic or likely pathogenic (P or LP) variants from the ClinVar database, as well as variants without annotations (after filtering out variants with a CADD score < 30, variants classified as benign or likely benign in ClinVar, and variants with allele frequency > 0.01 in the gnomAD and TWB databases, as well as our database) as previous described [1]. Variants were annotated using VEP (v104.3); the data for 1,171 cancer-related genes [2] were converted into an MAF file using vcf2maf (v1.6.21). The MAF file was loaded into R software for analysis and visualized using maftools (v2.6.05).

Mutations of histone-related genes, HCC-related long non-coding RNAs (lncRNAs), and non-coding driver genes were analyzed. We collected 114 histone-related genes from HistoneDB 2.0 [3] and selected the important variants using criteria similar to the constraints selected for cancer-related genes. We collected 74 HCC-related lncRNAs [4] and 36 non-coding driver genes, as described by Rheinbay et al. [5], then analyzed their alterations to explore their roles in the onset and progression of HCC [1].

**Copy number variation**

For copy number alteration (CNA) analysis, we used the Illumina Dragen CNV pipeline (v3.7), annotation by BEDTools and GENCODE gene data (GRCh38, V37), analysis in R, and visualization with the circlize package (v0.4.13).

**Structure variant (SV) calling**

SV merge by SURVIVOR (v1.0.7) (max distance, 1000; minimum size of SVs, 30; and minimum number of supporting callers, 1), annotate and query of SVs vcf file by BCFtools, and final annotated by AnnotSV (v3.0) were performed. For analysis of somatic SVs, we used gnomAD and WGS of 1491 samples from TWB and our database to generate a reference set. Then, we compared these SVs with non-cancerous and benign liver tissues to identify HCC-related somatic SVs. We used probability of being loss-of-function intolerant (pLI) ≥ 0.9 to select important SVs [6, 7]. The SV included cancer driver gene(s) was selected as HCC-related driver SV.

**Mutational signature analysis**

Mutational signature analysis was performed using the non-negative matrix factorization method in the R package NMF [8], along with BCFtools (v1.9) to filter variants (FILTER=PASS, ALT frequency > 0.1, DP > 20, QUAL > 30) [9]. Reference mutational signatures were obtained from the Catalogue of Somatic Mutations in Cancer (COSMIC) v3 (May 2019) [10]

**RNA extraction and RNA sequencing (RNA-seq)**

Total RNA was extracted from tissue samples using a NucleoSpin® RNA Kit (Macherey-Nagel, Duren, Germany), in accordance with the manufacturer’s instructions and our existing method [11]. The quality, quantity, and integrity of total RNA were evaluated using a NanoDrop1000 spectrophotometer and a Bioanalyzer 2100 (Agilent Technologies, Santa Clara, CA, USA). Samples with an RNA integrity number > 6.0 were used for RNA-seq. A ribosomal RNA (rRNA)-depleted, barcoded total RNA library was generated using a Total RNA Library Preparation Kit (Illumina). Brief, 100 ng of total RNA were treated with a Ribo-Zero Gold rRNA Removal Kit (Illumina) to deplete rRNA. The resulting RNA was fragmented, reverse transcribed, and synthesized to generate double-stranded complementary DNA (cDNA), which was subsequently ligated to barcoded adapters and polymerase chain reaction-amplified to construct a cDNA library. The libraries were sequenced on the NovaSeq 6000 instrument (Illumina), using 2×76-bp paired-end sequencing flow cells in accordance with the manufacturer’s instructions. Approximately 100 million pairs of reads were generated from each library.

**RNA differential expression (DE) analysis**

RNA DE analysis was performed as previously described [11]. Briefly, after RNA-seq, the raw data were processed by the Illumina Dragen RNA Pipeline v3.6 and v3.7, reads were aligned to GRCh38, and bam files were used to quantify gene expression based on GENCODE v35. Transcript per million (TPM) values and read counts were used for differential expression gene (DEG) analysis.

To characterize DE between HCC tumor and non-tumor tissues, the R package Deseq2 (v1.36.0) was used after normalization and the removal of genes for which there were few or no reads. Genes with log_2_ fold change ≥ ±1.5 and adjusted p-values < 0.01 were considered DEGs in DEseq2 analysis; these genes were visualized using volcano plots with EnhancedVolcano (v11.6.0). To identify DEGs important for survival, we compared tumor samples with expression levels in the top 25 % and bottom 25%.

**Gene fusion analysis**

Fusion genes were predicted with Illumina Dragen Bio-IT version 3.7. Locus and transcripts information were obtained from GENCODE version 35; the human genome version is GRCh38. High-confidence fusion genes were selected using the following criteria: (1) number of split read and supported fragment alignment (NumSplitReads and NumPairedReads) > 5, (2) prediction score from Dragen > 0.9, and (3) absence in adjacent normal samples.

**Metatranscriptomic analysis**

Kraken2 (v2.1.1) was used for analysis of metatranscriptomic data [12]. The July 2020 Kraken2 database was used to annotate human, viral, archaea, bacterial, and fungal genes; National Center for Biotechnology Information (NCBI) RefSeq was used as the reference sequence database [13]. For the standard Kraken output, the lowest common mapping data of each k-mer in the sequence(s) were used to identify reads containing both human and HBV taxon IDs (#9606 and #10407, respectively). RNA-seq reads were then extracted from the fastq file, and Arriba was used to detect gene fusions in RNA-seq data, as previously described [2, 14].

**RNA alternative splicing (AS) analysis**

Bioinformatics identification of AS events was performed as previous described [15]. Briefly, high-confidence transcripts were identified in conditions where the TPM expression value was > 0.1 in more than 80% of the sample. Proportion spliced-in (PSI) value was calculated with SUPPA2 [16] and used to estimate abundances of AS event. The types of AS event (e.g., skipping exon, retained intron, mutually exclusive exon, alternative 5’ splice site, alternative 3’ splice site, alternative first exon, and alternative last exon) were collected from the GENCODE v35 annotation file. The splicing events, which contained the PSI value for all samples, were used for downstream analysis.

**Cell score**

For evaluate of tumor microenvironment characteristics, xCell [17] was used to calculate the enrichment scores of 64 immune and stromal cell types from gene expression data, as described previously [14].

**Statistical analyses**

Survival analysis was performed using survival v. 3.2.3 [18] using the log-rank test for comparisons among groups. Kaplan–Meier survival curves were visualized using ggfortify v. 0.4.10 [19] and surviminer v.0.4.9*.* Patients who had undergone transplantation were excluded for survival analysis*.* The wilcoxon rank sum test was performed for comparisons between two groups, using the R package ggpubr (v0.4.0).

**Supplementary figures**

**Fig. S1.**

**Fig. S1. Survival curve indicating the effect of the HCC treatment approach on patient survival.** OP: Operation, OP/CT: Operation/Chemotherapy, OP/CT/TACE: Operation/Chemotherapy/Trans-Arterial Chemo-Embolization, OP/IMM: Operation/Immune drug therapy, OP/TACE: Operation/Trans-Arterial Chemo-Embolization.

**
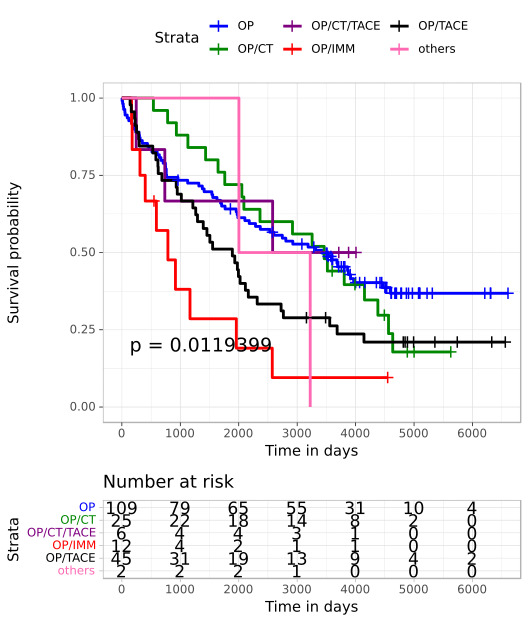
**

**Fig. S2.**

**Fig. S2. Mutation frequency comparison and correlation of mutated genes.** Comparison of frequencies of 17 common mutated genes between subgroups of Taiwanese HCCs (CMUH_HCC) and TCGA-LIHC [full cohort and split by major racial subgroups, namely Asian (TCGA_LIHC_A) and White people (TCGA_LIHC_W)].


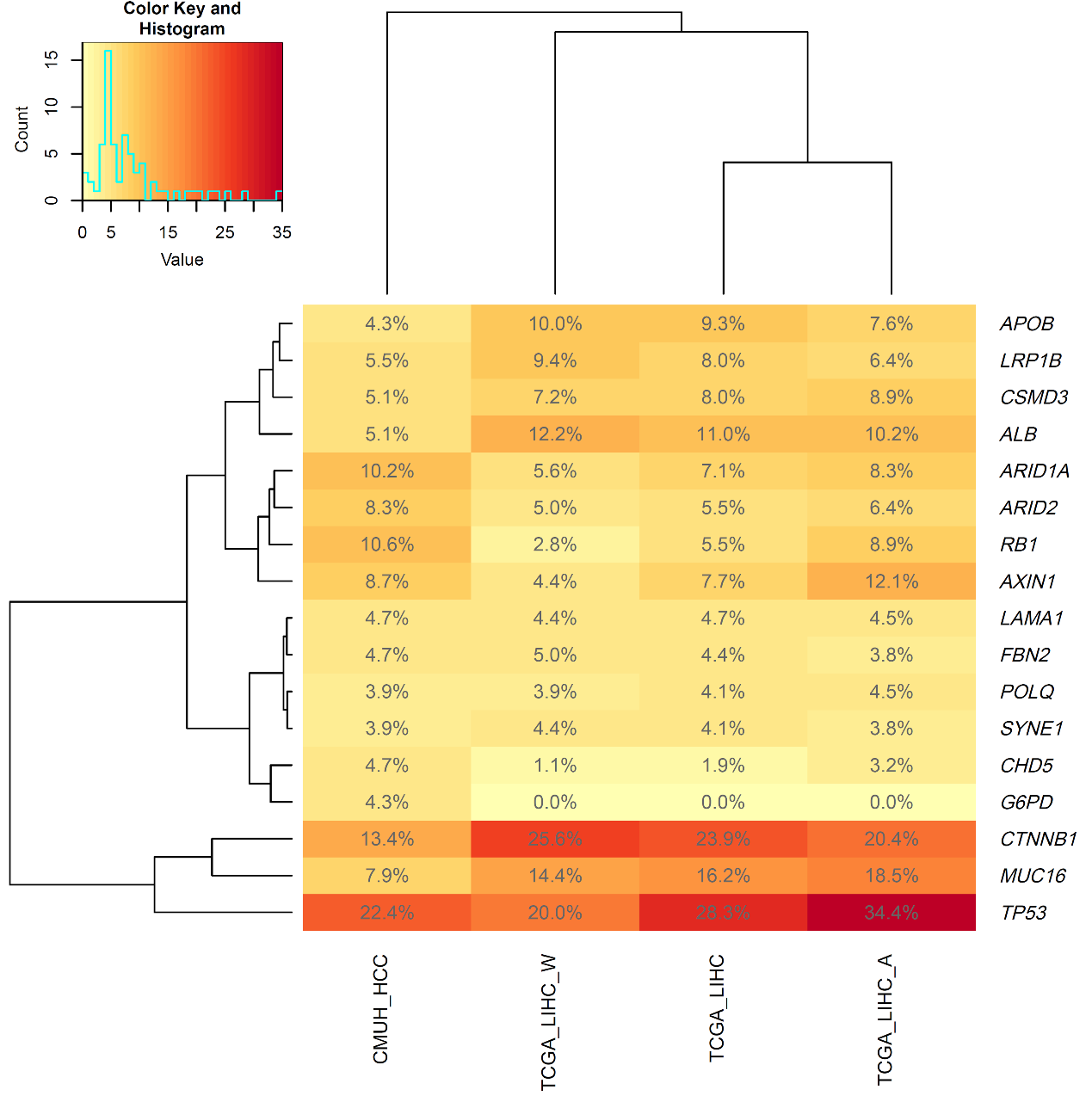


**Fig. S3.**

**Fig. S3. Correlation between clinical data and etiology of HCC. (**A) Female has more HCV-related HCC. (B) HCV-related HCC has more cirrhosis. (C) No correlation of survival with etiology of HCC. Other: no HBV or no HCV or no non-HBV/non-HCV or no dual HBV/HCV; variants: HBV or HCV or non-HBV/non-HCV or dual HBV/HCV.

(A)

| Gender\HCV | No | Yes |
| --- | --- | --- |
| Female | 25+2* | 36 |
| Male | 109+8* | 65+9* |
| p = 0.0127 | | |

*Patients with double cancer count

p-value by Chi-square test or Fisher’s exact test when appropriated.

(B)

| Cirrhosis\HCV | No | Yes |
| --- | --- | --- |
| No | 65+6* | 25+1* |
| Yes | 69+4* | 76+8* |
| p = 2.961e-05 | | |

*Patients with double cancer count

p-value by Chi-square test or Fisher’s exact test when appropriated.

(C)


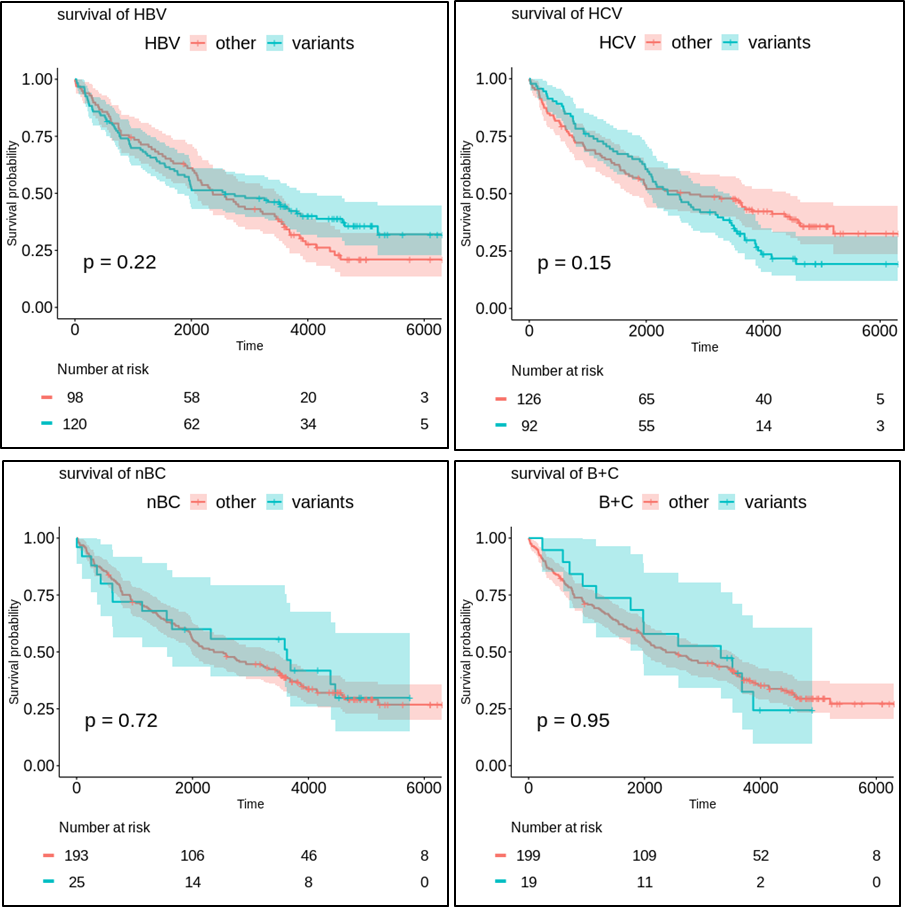


Patients were excluded if transplanted receive (36 cases) in survival analysis.

**Fig. S4.**

**Fig. S4. Correlation between clinical data and *TERT* mutation.** (A) *TERT* mutation correlation with HCV-related HCC, but not HBV. (B) *TERT* mutation correlated with poorer survival of HCC. Other and variants represent without or with *TERT* promoter mutation, respectively.

(A)

| *TERT*\HBV | No | Yes |
| --- | --- | --- |
| Mutation | 69+7* | 40+4* |
| Wild-type | 41+4* | 85+4* |
| p = 2.812e-06 | | |

*Patients with double cancer count

p-value by Chi-square test or Fisher’s exact test when appropriated.

| *TERT*\HCV | No | Yes |
| --- | --- | --- |
| Mutation | 45+5* | 64+6* |
| Wild-type | 89+5* | 37+3* |
| p = 5.024e-06 | | |

*Patients with double cancer count

p-value by Chi-square test or Fisher’s exact test when appropriated.

(B)


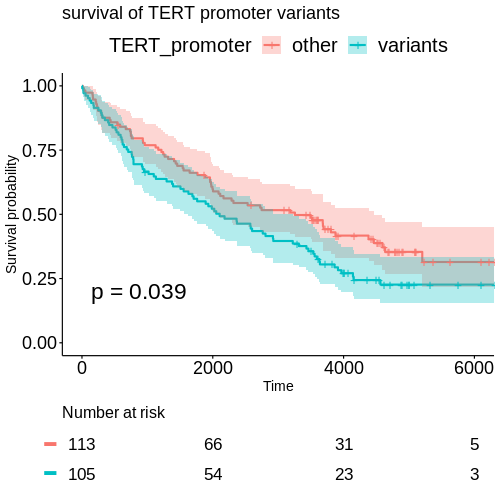


Patients were excluded if transplanted receive (36 cases) in survival analysis.

**Fig. S5.**

**Fig. S5. Chromosome alterations and survival correlation of HCC.** (A) The heatmap of chromosomal alterations. Left: Unsupervised hierarchical clustering using chromosomal alterations, and the association with *TERT* mutations and etiological groups. Upper: chromosome association. Right: the meaning of color markers. The most frequently altered chromosome arms include 1q, 6p, 7, 8q, 17q gains and 4q, 8p, 13p, 16, 17p losses. (B) The survival correlation between chromosomal changes and patient’s survival. The overall survival was significantly better in the 17p loss compared with other group (p = 0.05), but 7q gain was associated with poorer survival (p = 0.053). (C) The genes in the chromosomal alteration regions and the correlation between involved genes and survival. The same chromosomal region contains many oncogenes and tumor suppressor genes (TSGs) that can increase or decrease simultaneously.

(A)


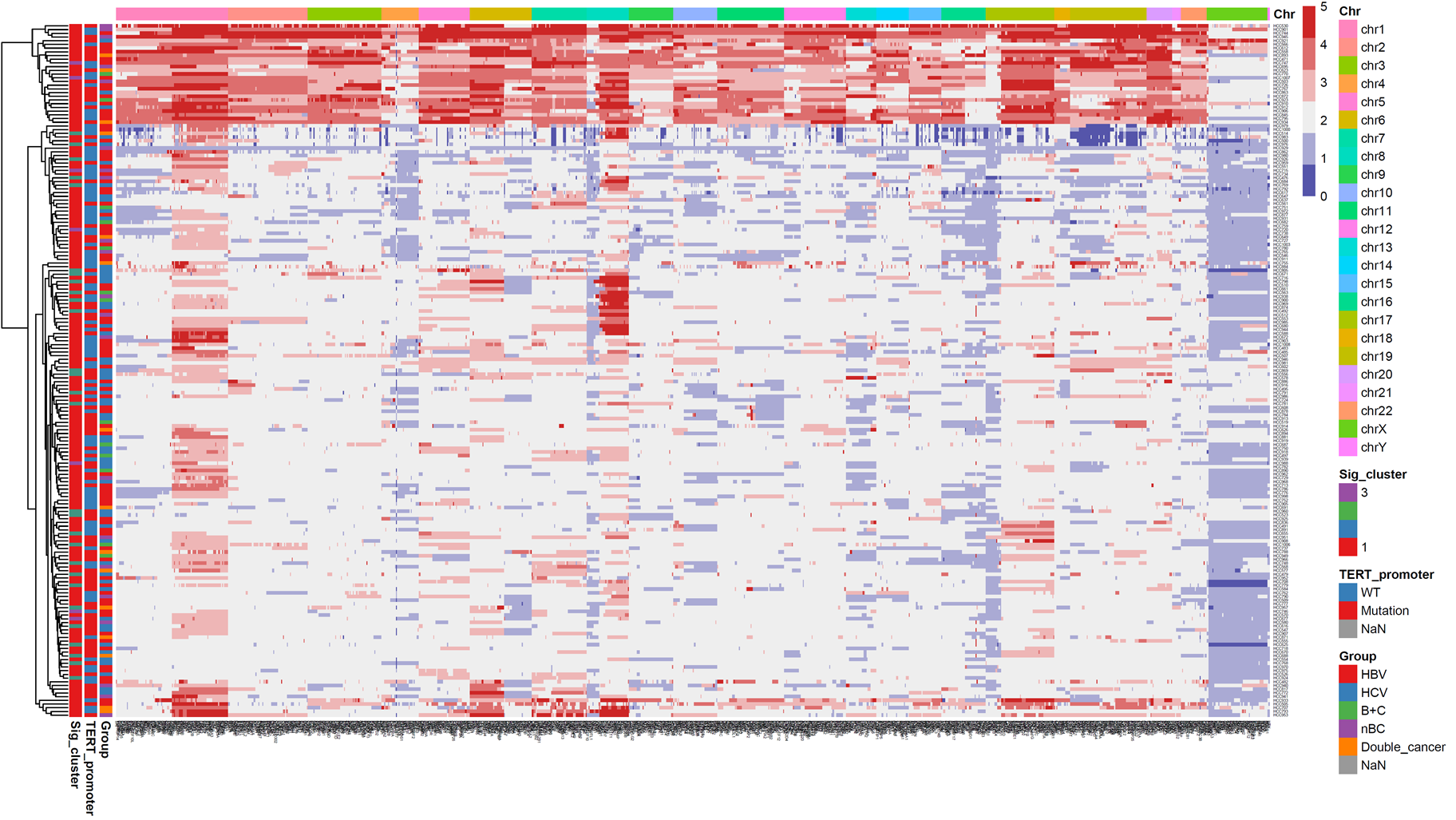


(B)


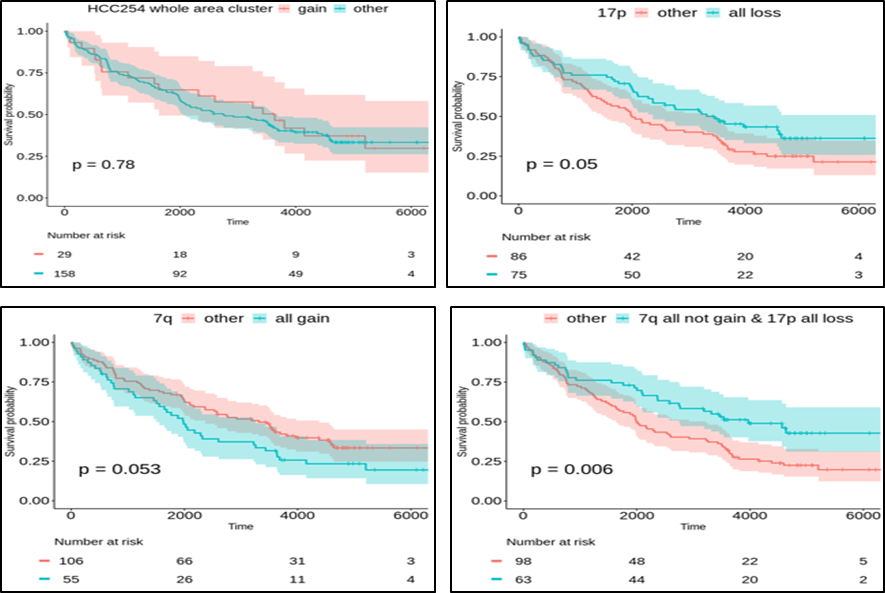


(C)


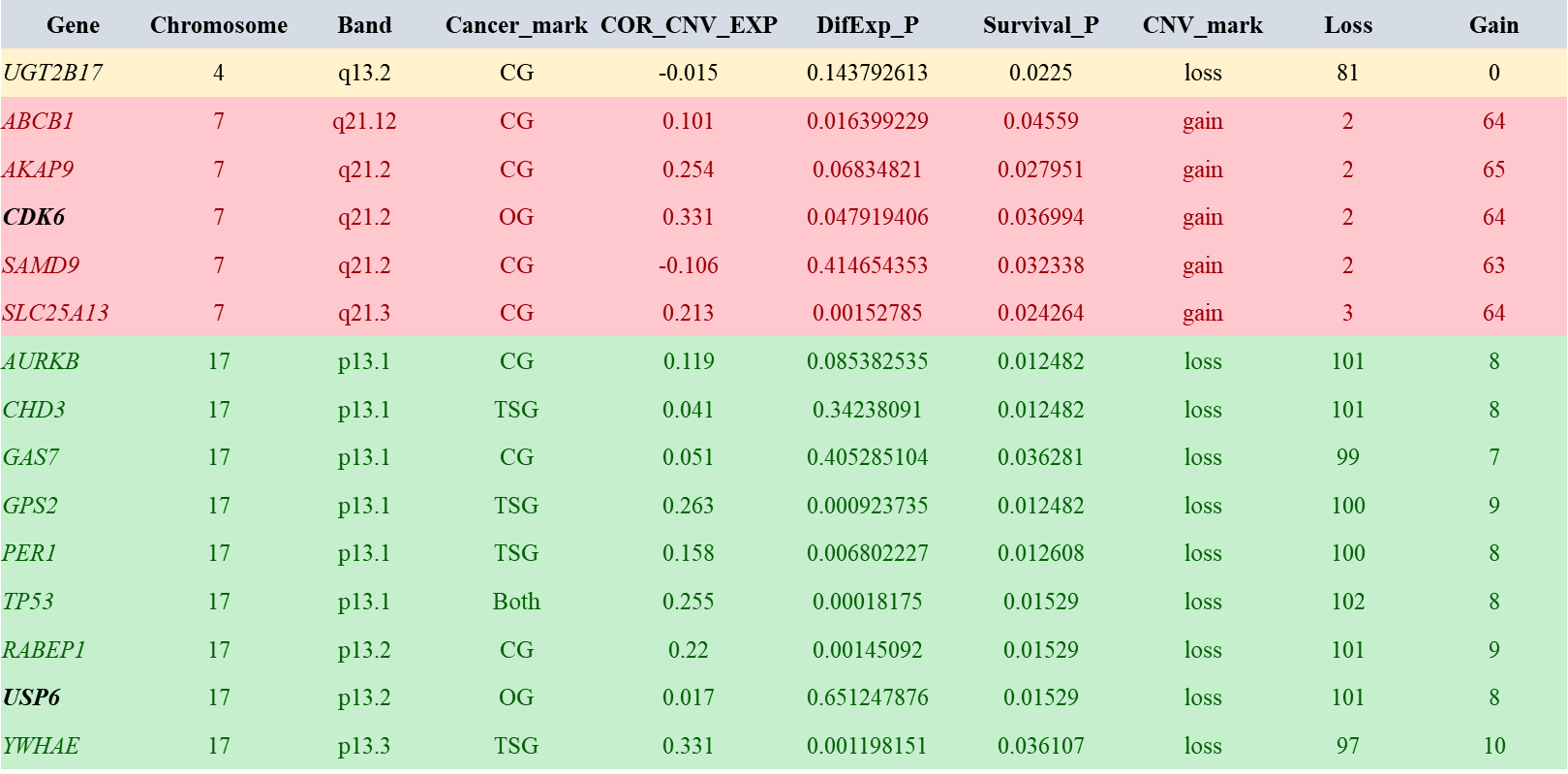


**Fig. S6.**

**Fig. S6. The landscape of copy number alterations (CNAs) in 254 Taiwanese HCCs.**

(A) The circos plot of CNAs. Outer: copy alteration and correlation with down expression (green) or over-expression (red) and survival. Middle: chromosome marker. Inner: copy gain (red) and copy loss (green). Oncogene, tumor suppressor gene, and both characters are depicted in bold, italics, and bold italic, respectively. (B) Survival-related copy changes. CNV: copy number variation, CG: cancer-related gene, OG: oncogene, TSG: tumor suppressor gene, other: without gain or loss. (C) The correlation between different etiology and copy alterations.

(A)


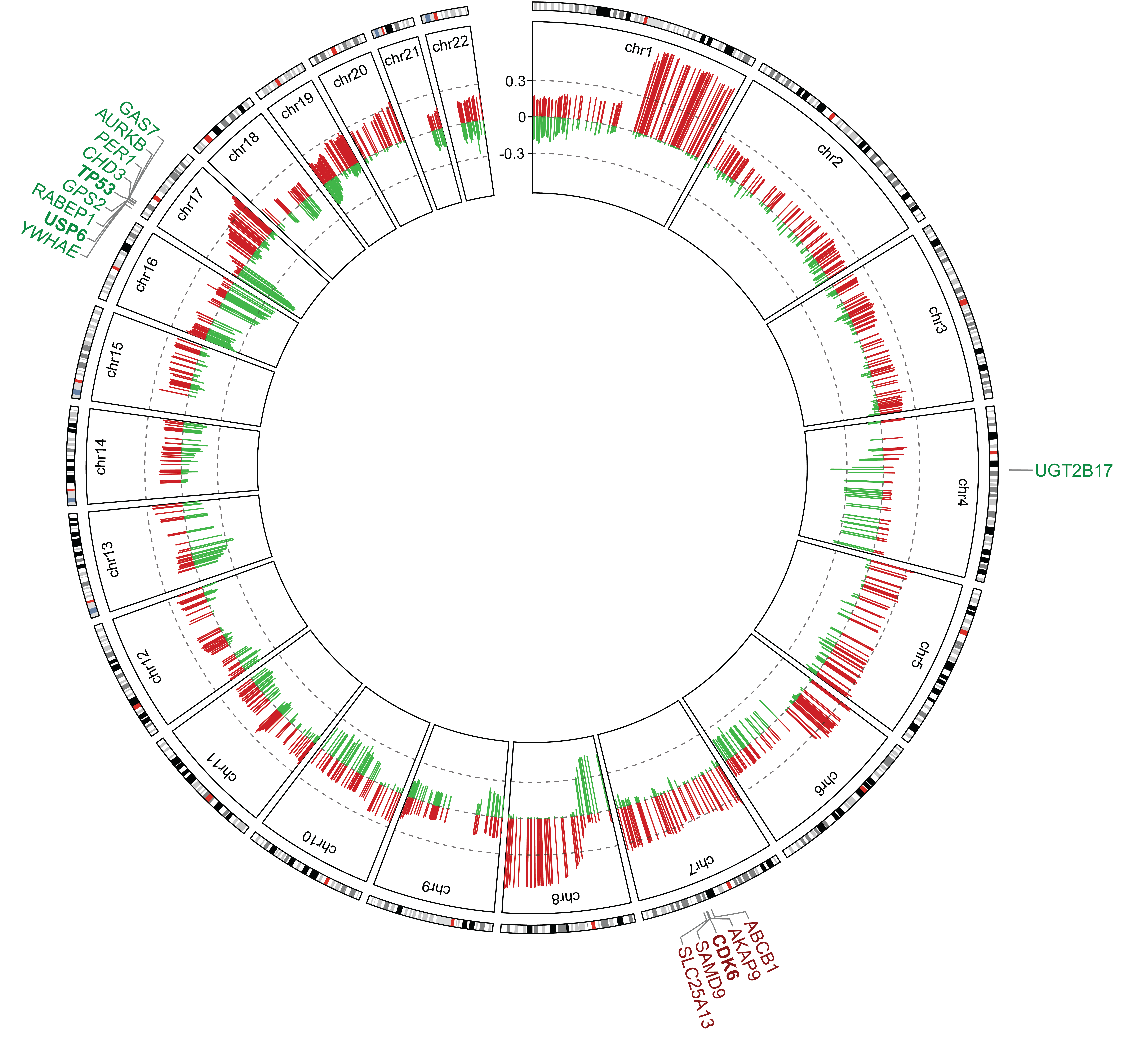


(B)


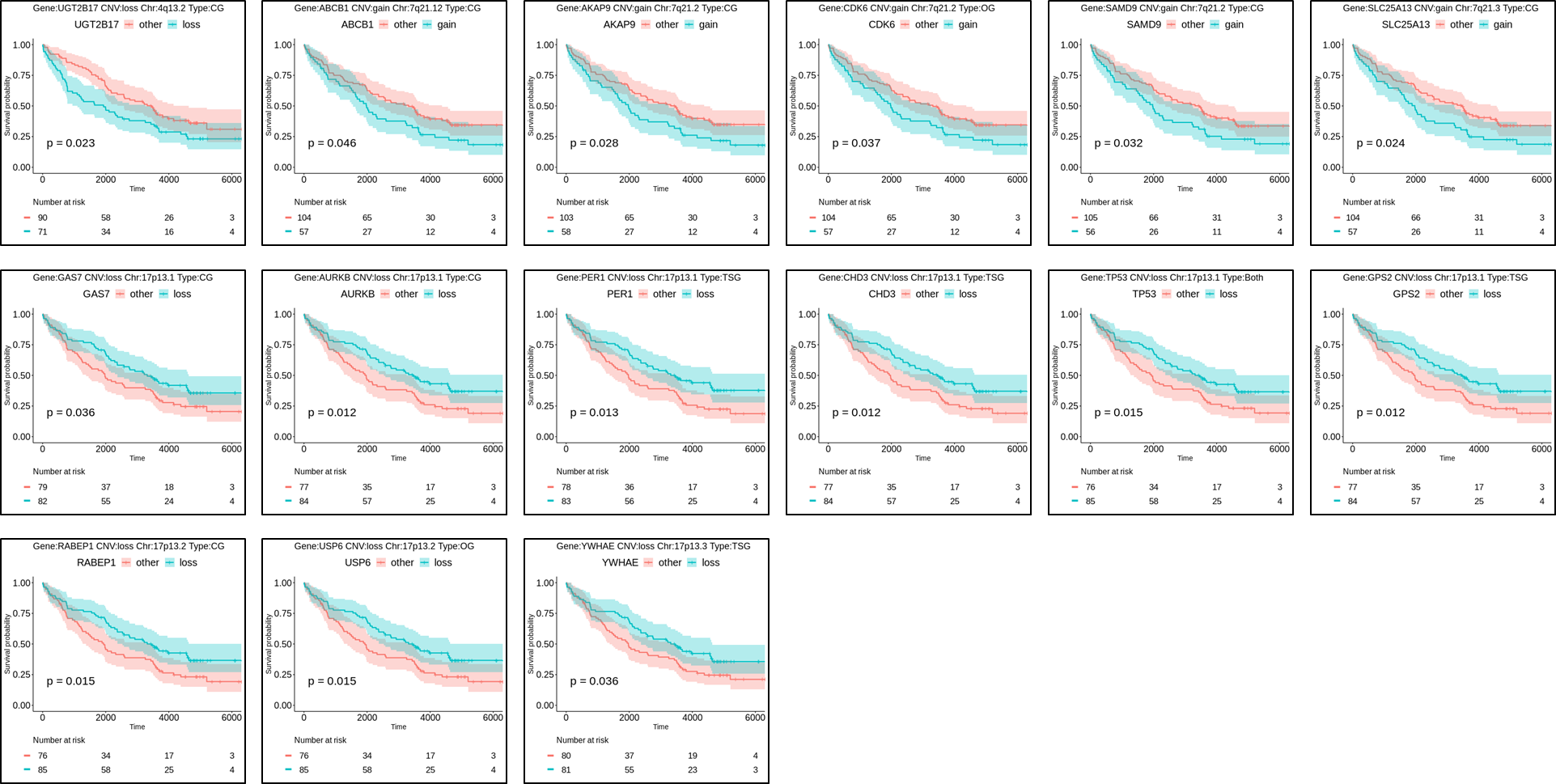


(C)


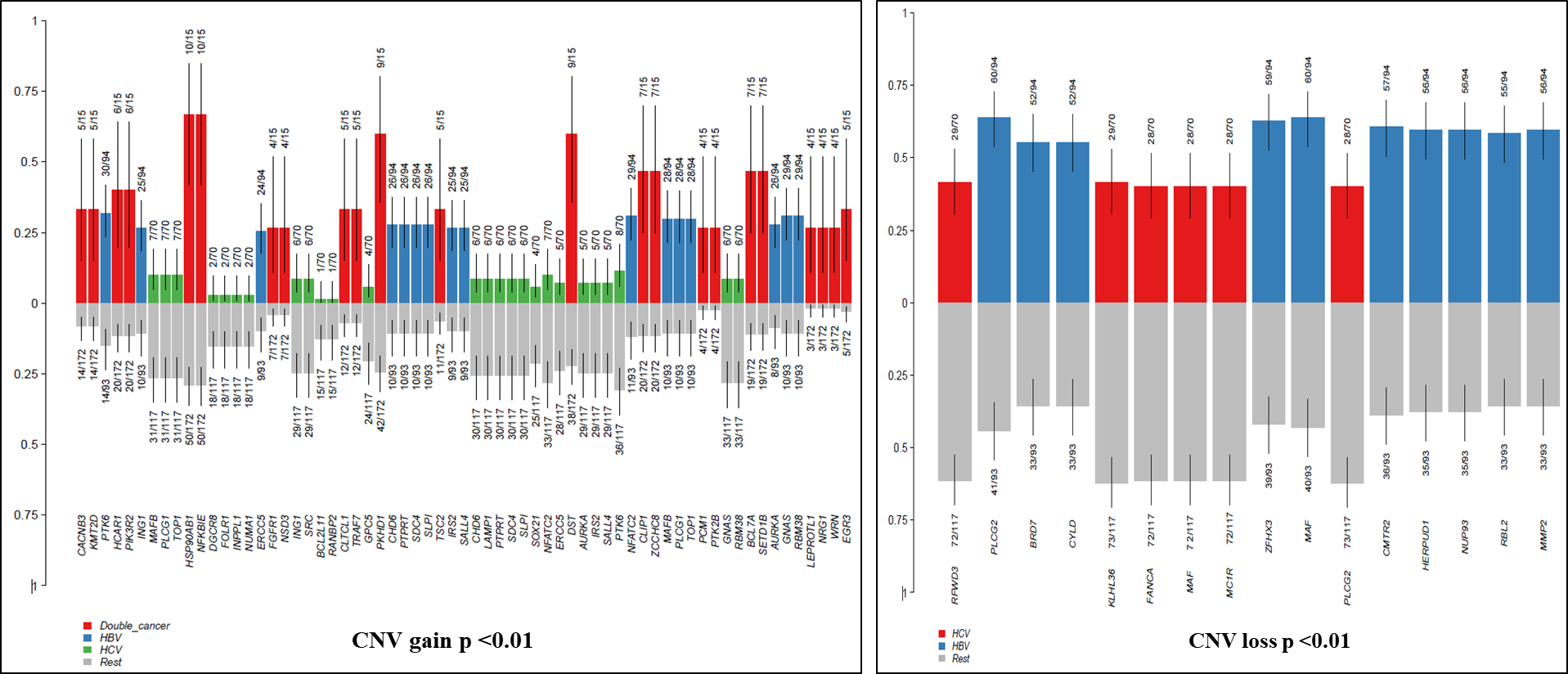


**Fig. S7.**

**Fig. S7. The results of analysis of structural variants (SVs).** The Venn plot of SVs among 254 HCCs in our cohort, Taiwan biobank (TWB), normal controls and liver benign lesions.


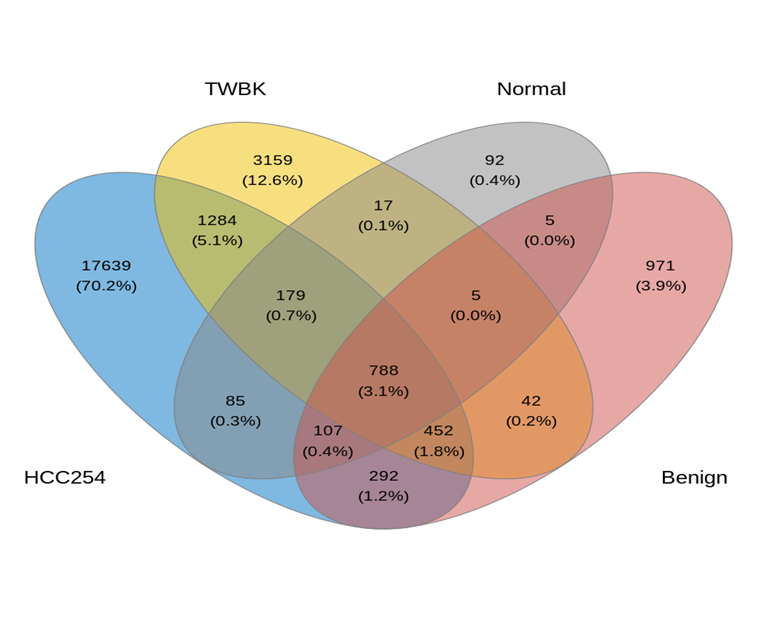


**Fig. S8.**

**Fig. S8. The correlation between probability of being loss-of-function intolerant (pLI) analysis and cancer-related genes.** pLI analysis of unique SVs with loss-of-function-intolerance of 254 HCCs in our cohort. p-value by t-test.


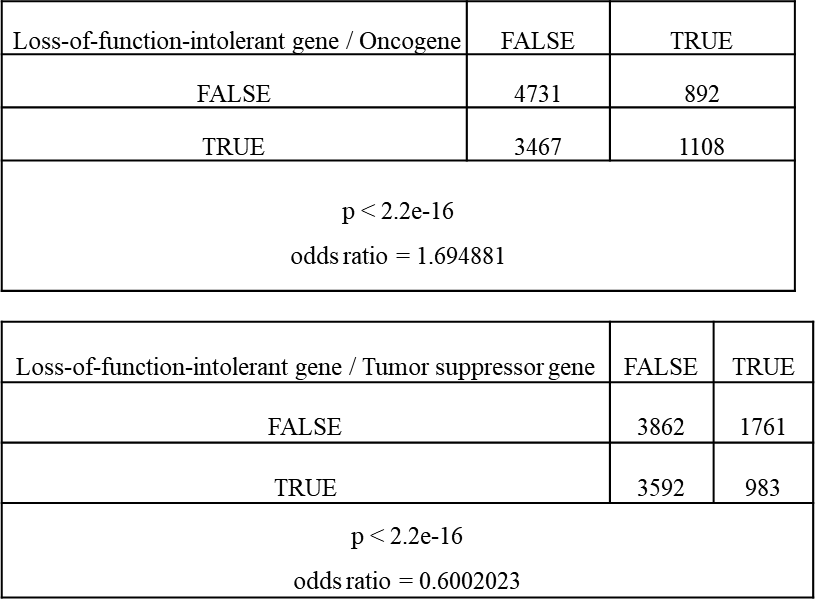


**Fig. S9.**

**Fig. S9. The Kaplan-Meier plot of 13 survival-related somatic SVs for 254 HCCs in our cohort.** The words in each figure represent**:** 1st lane is AnnotSV ID including SV- related chromosome (number), SV location from SV start to SV end and SV type; 2nd lane is GnomAD-related pLI score; 3rd lane is tumor suppressor gene (TS gene); 4th lane is oncogene (Onco_gene); 5th lane is cancer-related gene (Cancer_gene). SV 0 or 1 represent with or without SV, respectively.


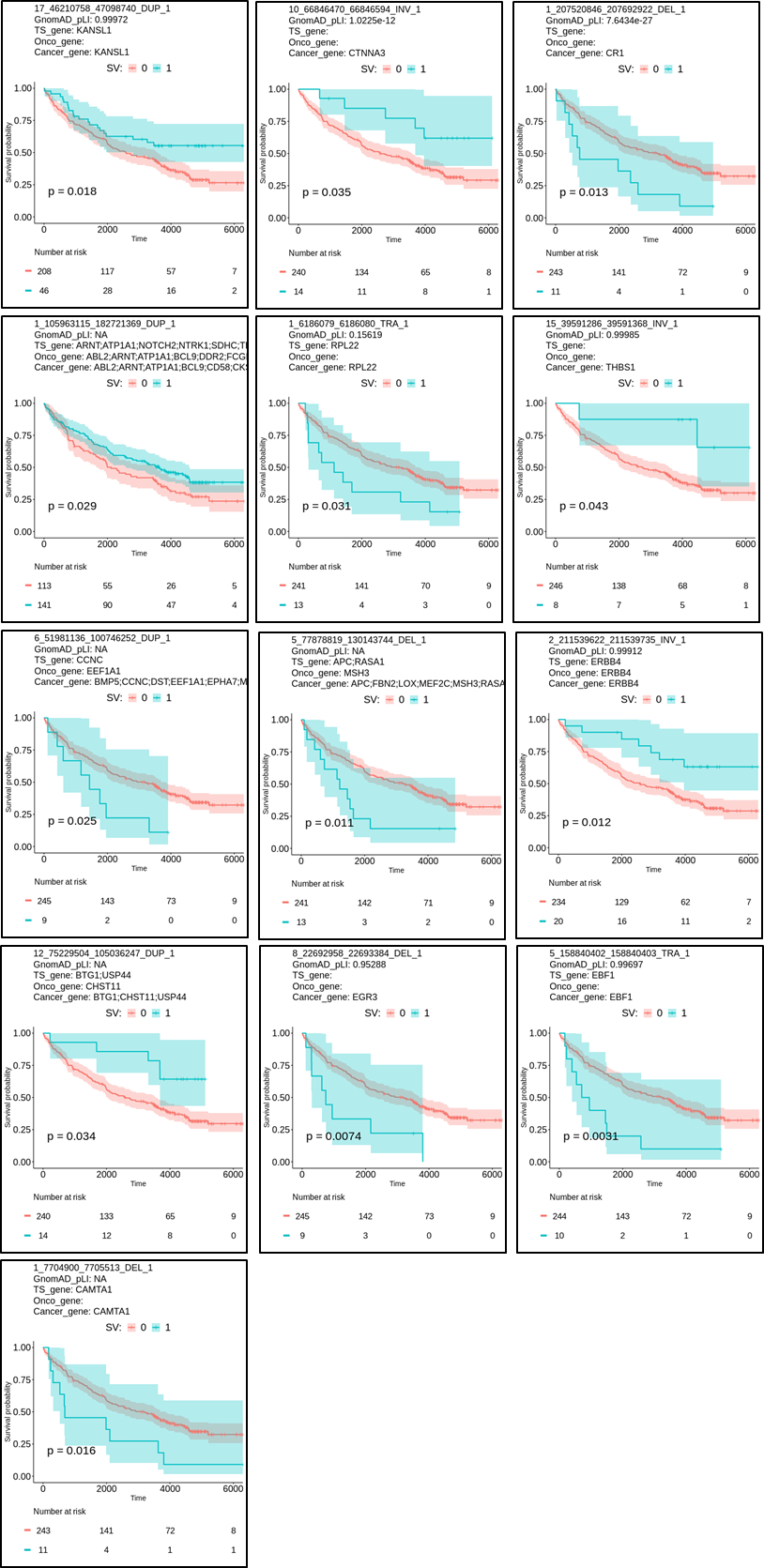


**Fig. S10.**

**Fig. S10. The details of novel survival-related somatic SVs for 254 HCCs in our cohort.**


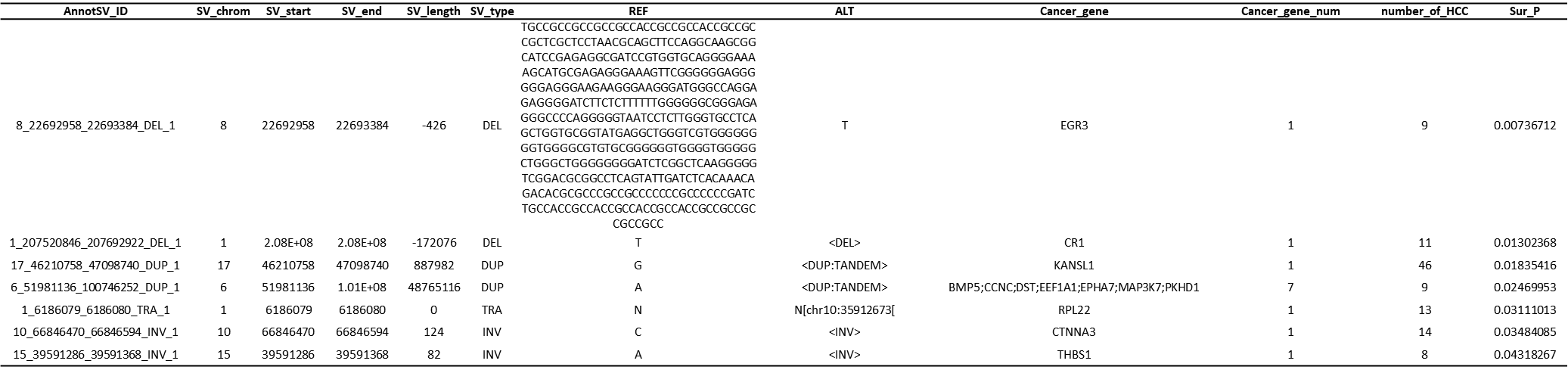


REF: Reference, ALT: Alteration, DEL: Deletion, DUP: Duplication, TRA: Translocation, INV: Inversion

Known SVs means following paper was reported.

Li Y, Roberts ND, Wala JA, Shapira O, Schumacher SE, Kumar K, et al. Patterns of somatic structural variation in human cancer genomes. Nature 2020;578:112-121.

**Fig. S11.**

**Fig. S11. Sanger sequencing validated the *HILS1* mutations detected by our WGS data.**

**
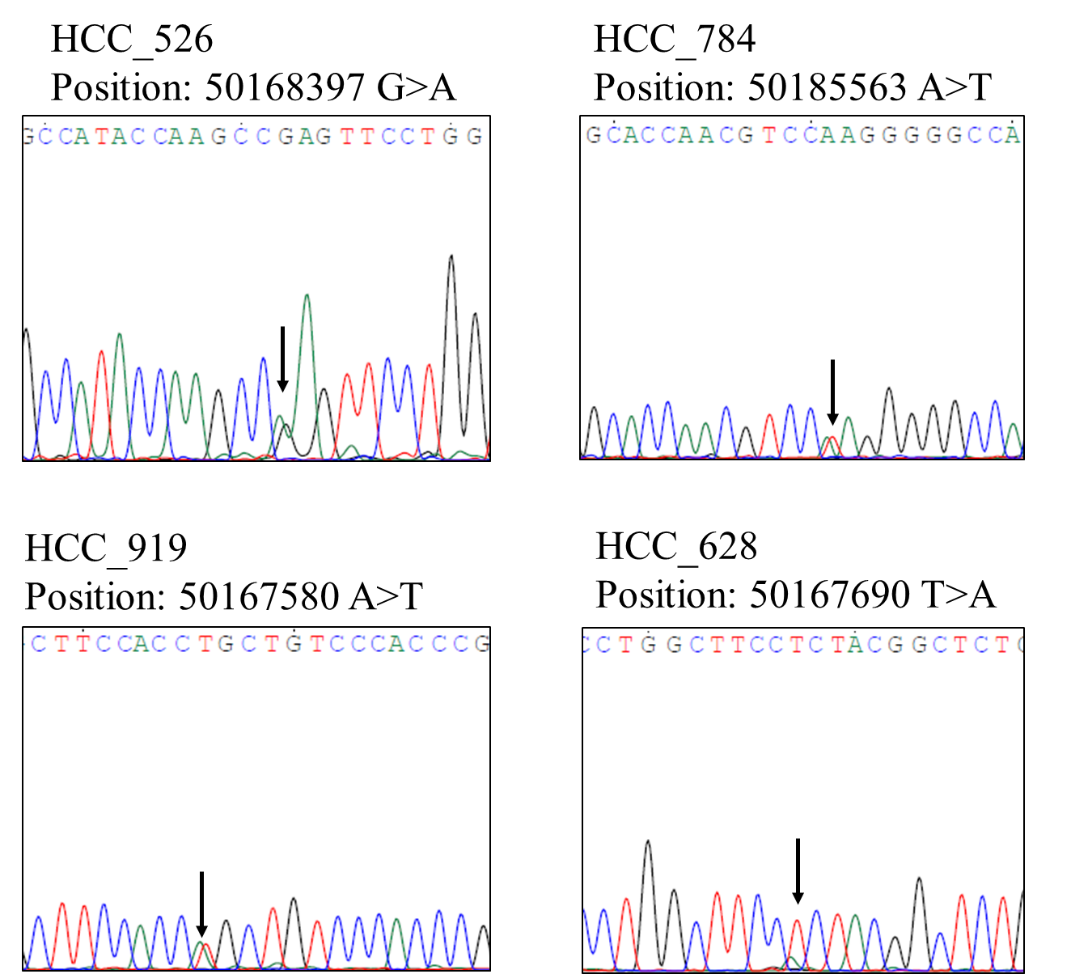
**

**Fig. S12.**

**Fig. S12.** **Mutational signature on 254 Taiwanese HCCs.** Cosine similarity by validated signatures in each sample and correlated with clinicopathological data and *TERT* mutation. Three signatures were extracted from 254 Taiwanese HCC and compared to COSMIC signatures catalogs.


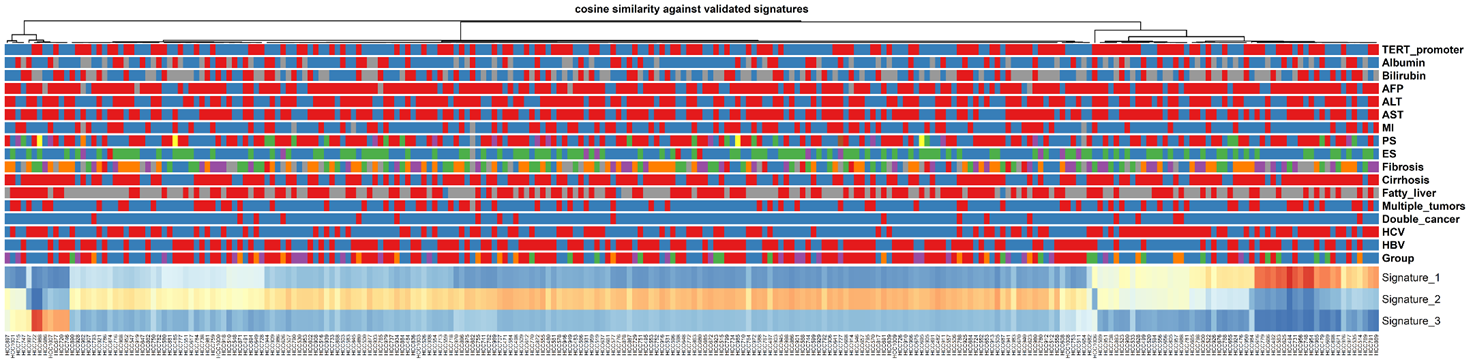


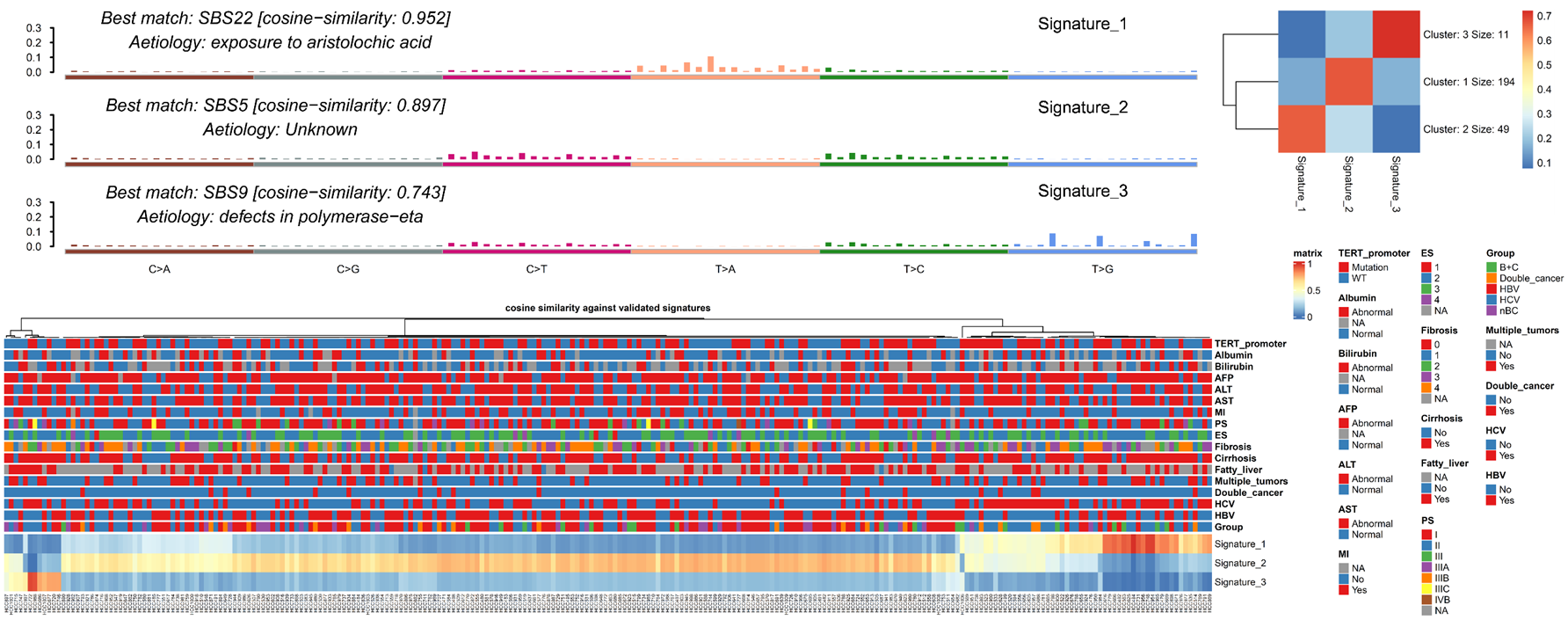


**Fig. S13.**

**Fig. S13. The results of correlation between fusion genes and survival.** (A) The results of fusion genes involving more than one case are shown. (B) The results of correlation of fusion genes with patient’s survival.

(A)


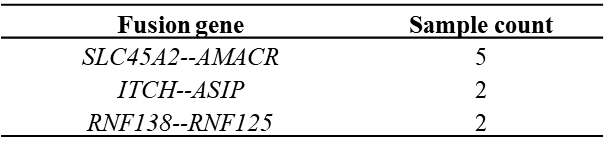


(B)


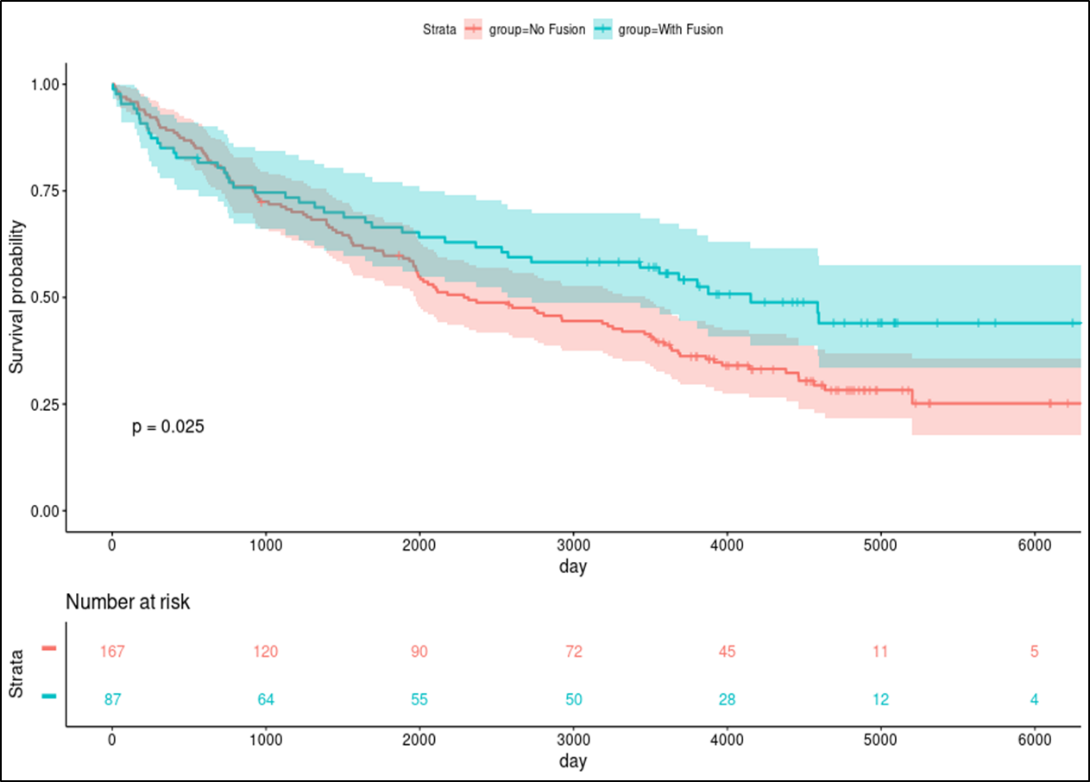


**Fig. S14.**

**Fig. S14. The results of correlation between HBV fusion genes and survival.** (A) RNA fusion. (B) DNA fusion.

(A)


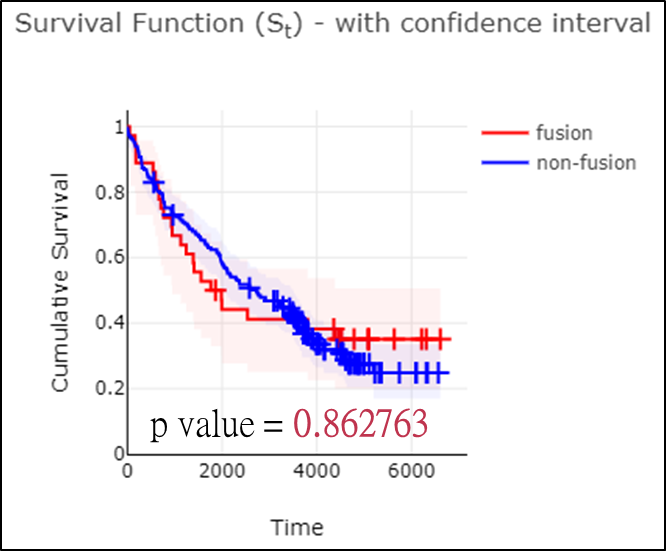


(B)


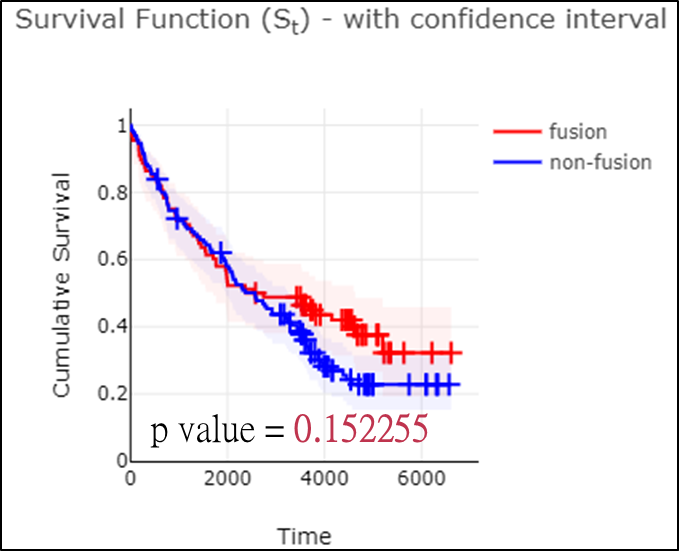


**Fig. S15.**

**Fig. S15. The results of analysis of alternative splicing (AS) events using proportion spliced-in (PSI).** Venn plot shows 345 intersection events using both 0.2 and 0.3 PSI.


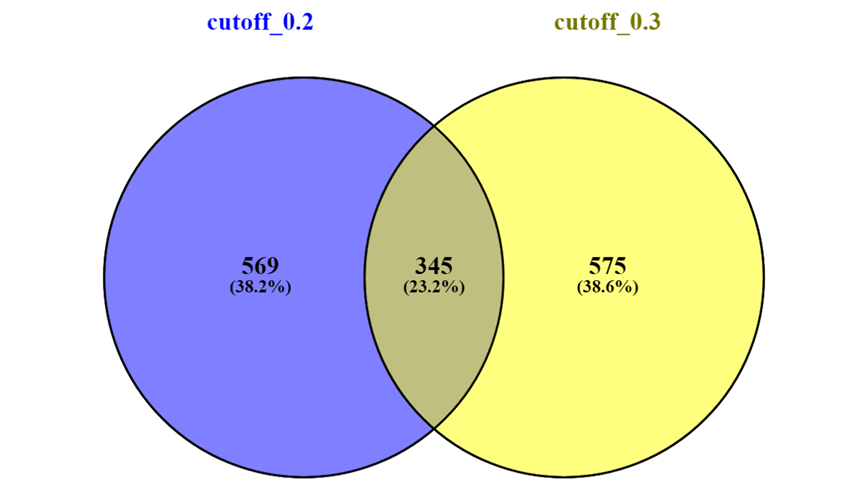


**Fig. S16.**

**Fig. S16. The results of analysis of survival-related AS events.** We only select the 98 and 86 AS events with both percentage (pct) 20 and 30 positive for good or bad condition for further study. SE: skipping exon, RI: retained intron, MX: mutually exclusive exon, AL: alternative last exon, AF: alternative first exon, A5: alternative 5’ splice site, A3: alternative 3’ splice site


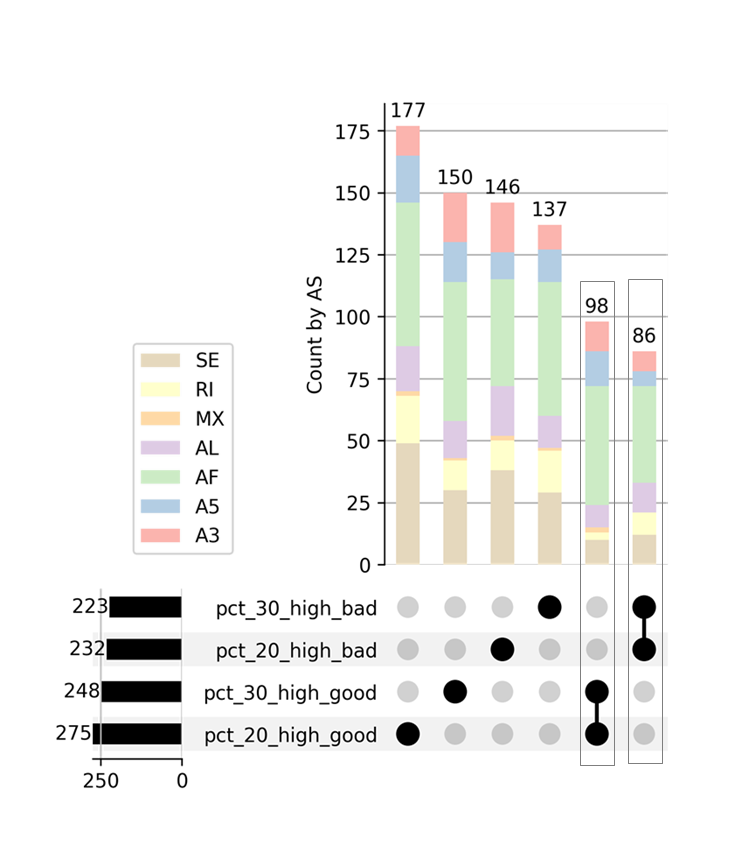


**Fig. S17.**

**Fig. S17. The novel alternative splicing genes correlation between good and poor survival-related genes.**


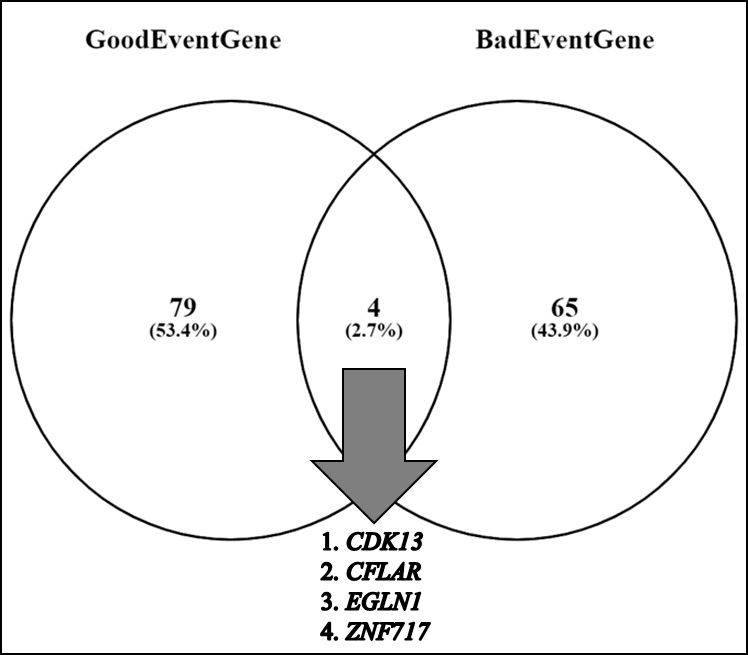


Known AS gene means following papers were reported.

[1] Chen H, Gao F, He M, Ding XF, Wong AM, Sze SC, et al. Long-Read RNA Sequencing Identifies Alternative Splice Variants in Hepatocellular Carcinoma and Tumor-Specific Isoforms. Hepatology 2019;70:1011-1025.

[2] Endo K, Terada T. Protein expression of CD44 (standard and variant isoforms) in hepatocellular carcinoma: relationships with tumor grade, clinicopathologic parameters, p53 expression, and patient survival. J Hepatol 2000;32:78-84.

[3] Wang XQ, Luk JM, Leung PP, Wong BW, Stanbridge EJ, Fan ST. Alternative mRNA splicing of liver intestine-cadherin in hepatocellular carcinoma. Clin Cancer Res 2005;11:483-489.

[4] Liu Z, Ye J, Khan AA, Chen J, Zhou L, Zheng S, et al. Genome-Wide Profiling of Alternative Splicing Signatures Associated with Prognosis and Immune Microenvironment of Hepatocellular Carcinoma. Med Sci Monit 2021;27:e930052.

[5] Li S, Hu Z, Zhao Y, Huang S, He X. Transcriptome-Wide Analysis Reveals the Landscape of Aberrant Alternative Splicing Events in Liver Cancer. Hepatology 2019;69:359-375.

**Fig. S18.**

**Fig. S18. The representative examples of size changes of coding amino acid for the gene with better survival-related alternative splicing (AS) event.** (A) to (N) for the amino acid size changes of *ALDH5A1*, *ATAD2B*, *C1orf109*, *CLPTM1*, *DAPK1****,*** *FOXK2*, *HSD3B7*, *HSPA12B*, *ING1*, *MPST*, *NCKIPSD*, *RNF40*, *ZBTB7A*, and *ZNF428*, respectively.

**
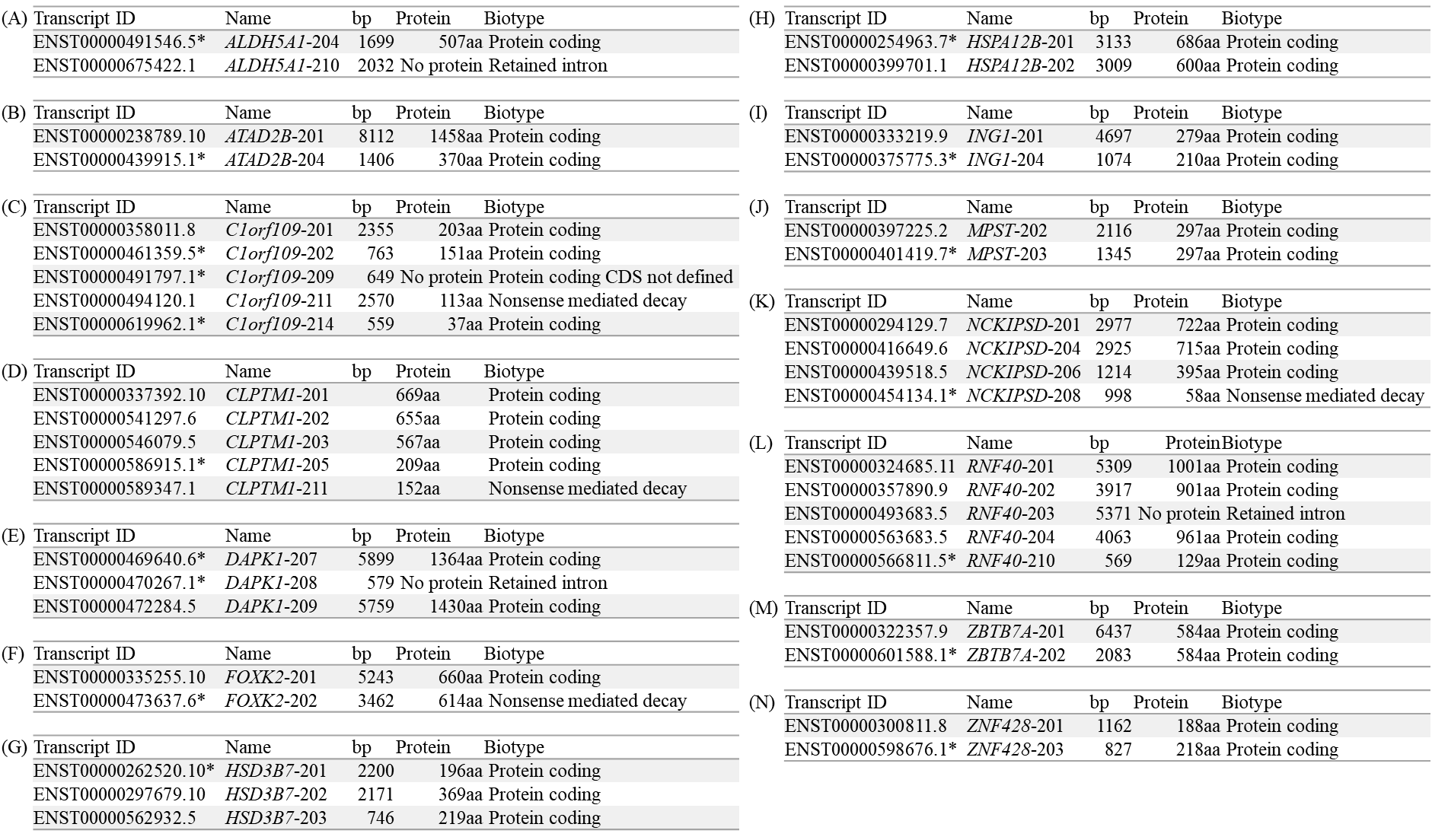
**

*: survival-related AS event. Oncogenes: *ATAD2B*, *C1orf109*, *CLPTM1*, *FOXK2*, *HSD3B7*, *HSPA12B*, *ING1*, *MPST*, *RNF40*, *ZBTB7A*

**Fig. S19.**

**Fig. S19. The representative examples of size changes of coding amino acid for the gene with poorer survival-related alternative splicing (AS) event.** (A) to (L) for the amino acid size changes of *BRD7*, *CDK13*, *CTNND1*, *DNM2*, *FAM20A*, *FHIT*, *NBN*, *PPP1R13L*, *RPL22*, *RSL1D1*, and *TRIM25*, respectively.

**
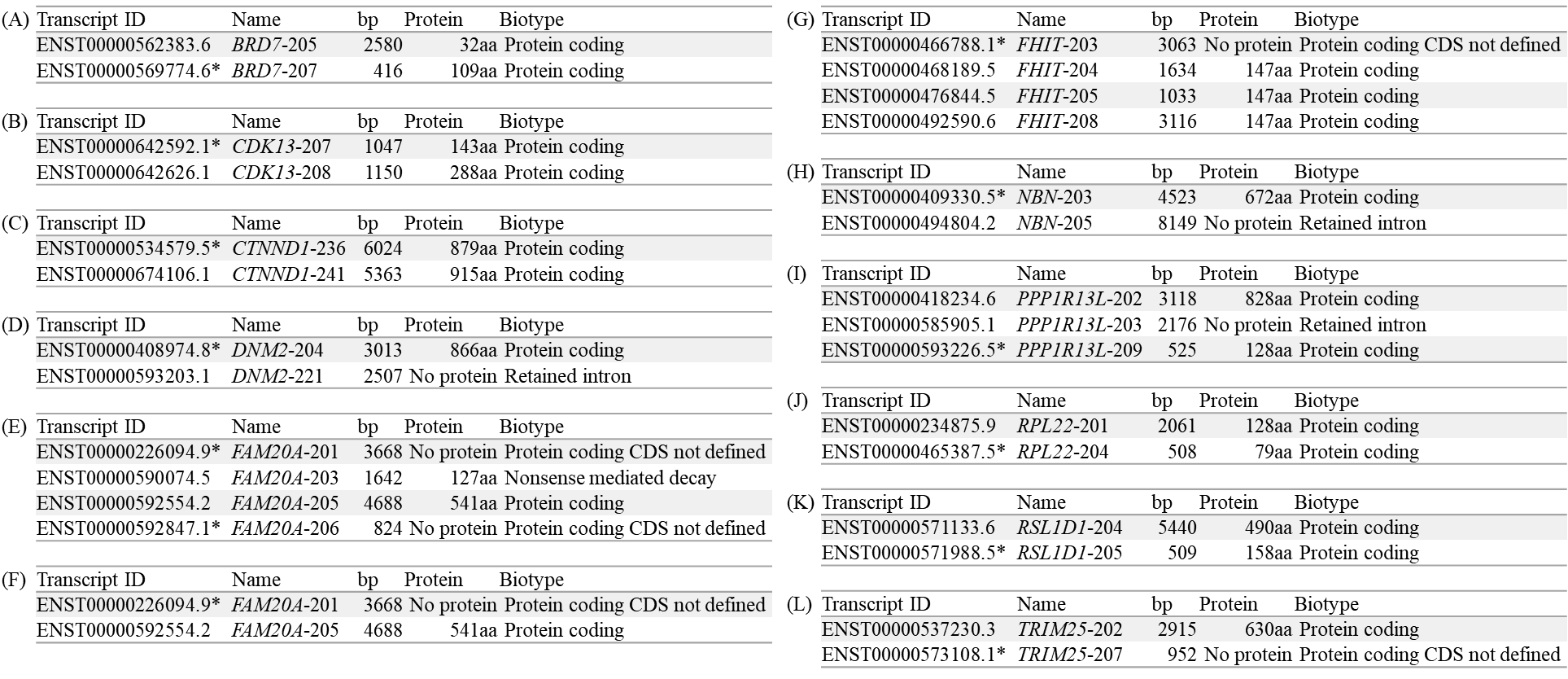
**

*: survival-related AS event

**Fig. S20.**

**Fig. S20. The results of correlation between the expression of immune checkpoint genes and genomic alterations including somatic mutations and copy alterations.** (A) *ARID1A* mutation with higher *CD70* expression. (B)-(V) copy alterations of *AKT1*, *AKT2*, *ARID1A*, *ARID1B*, *AXIN1*, *GNAS*, *IDH2*, *NF1*, *SMARCA4*, *STAT3* and *TSC2* influencing the expressions of different immune checkpoint genes.

**
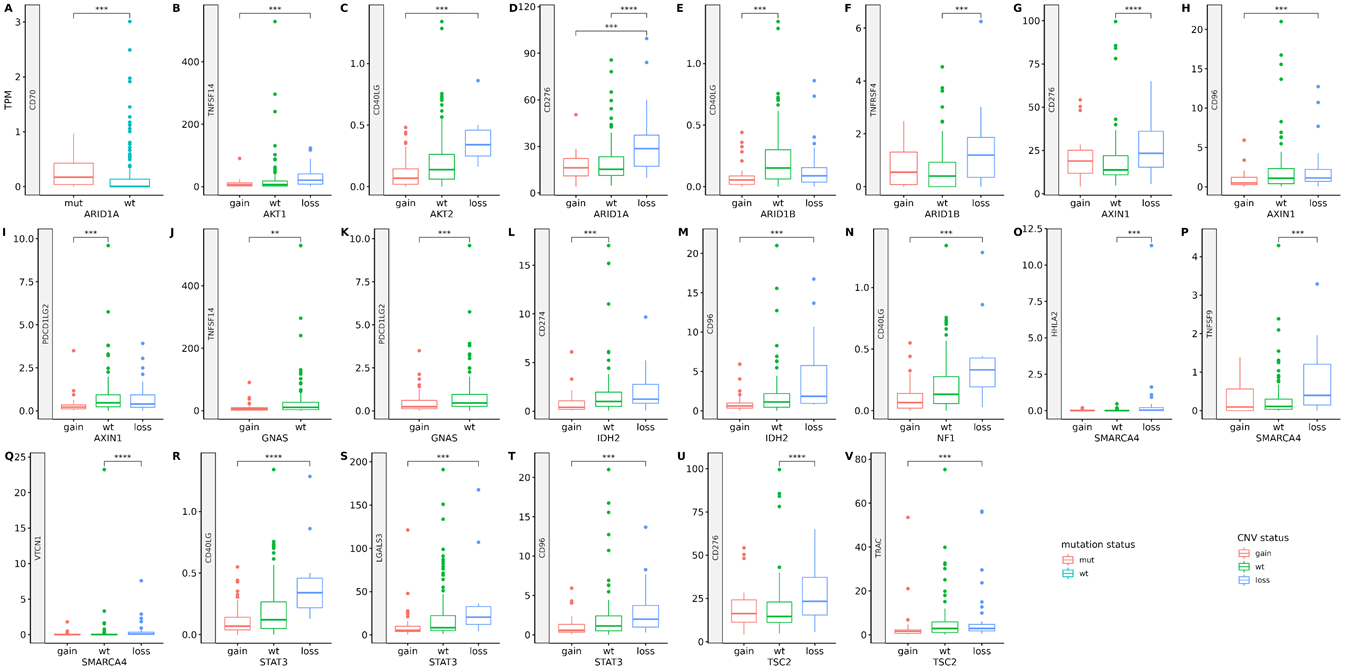
**

**Fig. S21.**

**Fig. S21. The correlation of point mutations and copy alterations of cancer-related genes and quantity of 64 immune and stroma cells and 3 scores.** (A) Point mutations of cancer-related genes and immune, stroma cells and scores. (B) Copy alterations of cancer-related genes and immune, stroma cells and scores.

(A)


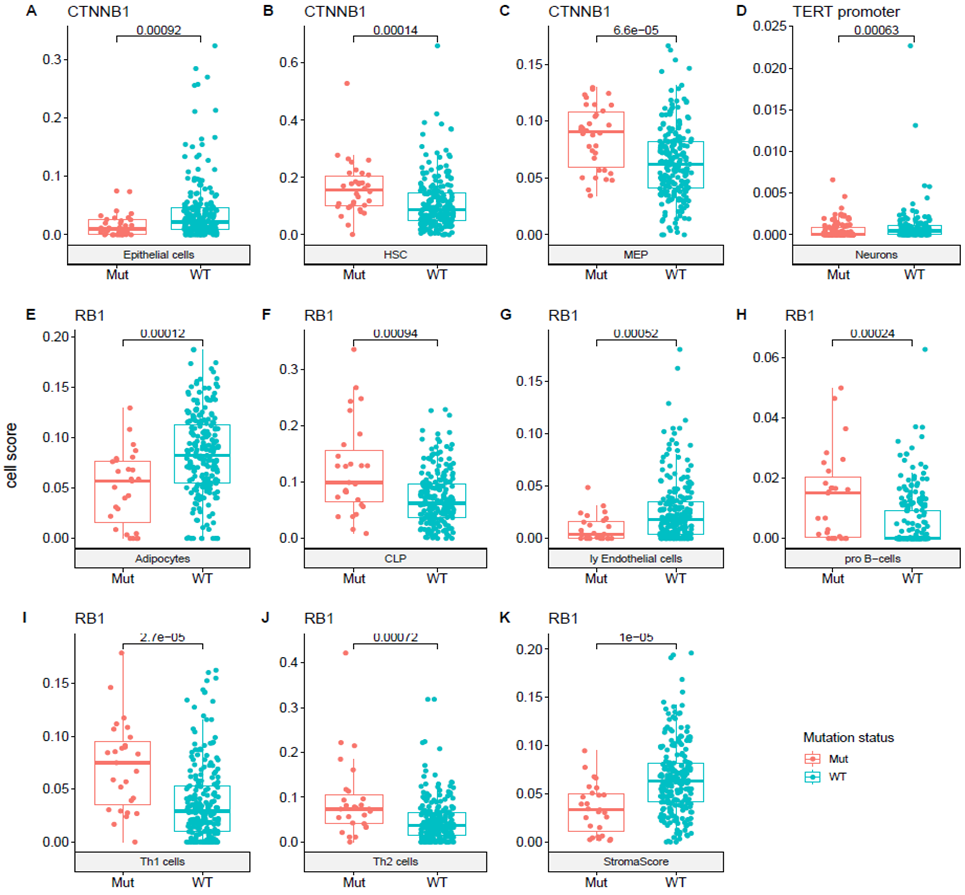


(B)


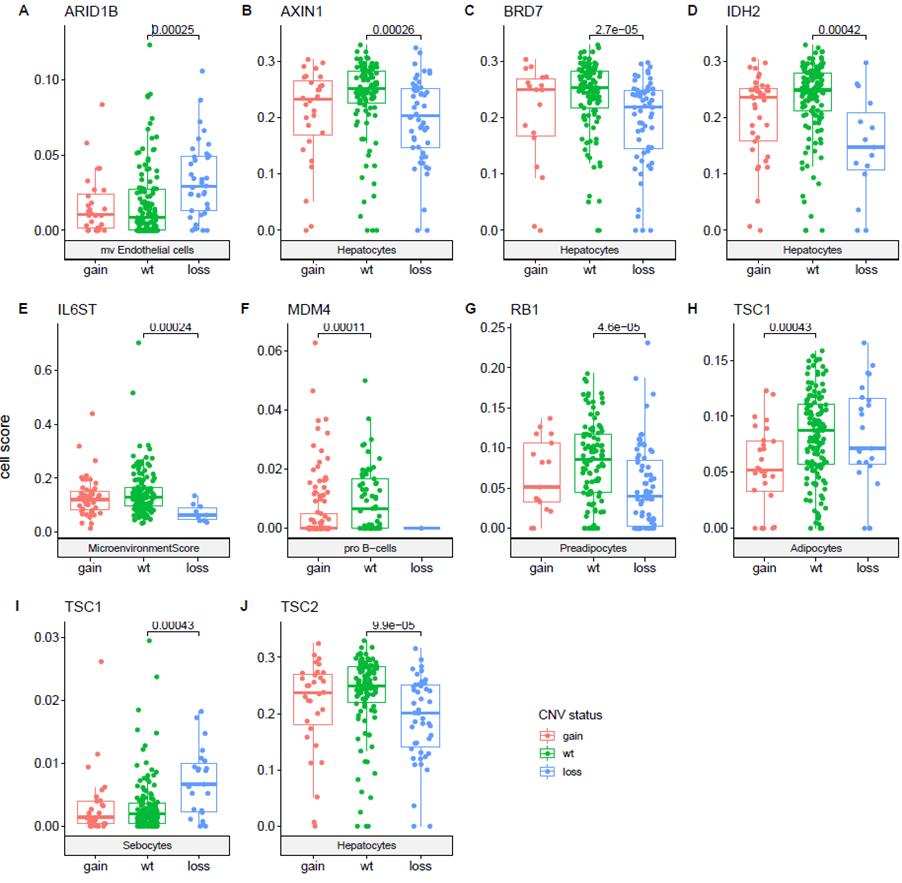


**Fig. S22.**

**Fig. S22. The results of correlation between tumor microenvironment and SVs.** The details of the only SV (AnnotSV_ID: 1_105963115_182721369_DUP_1) with significance after further selection using adjusted p-value.


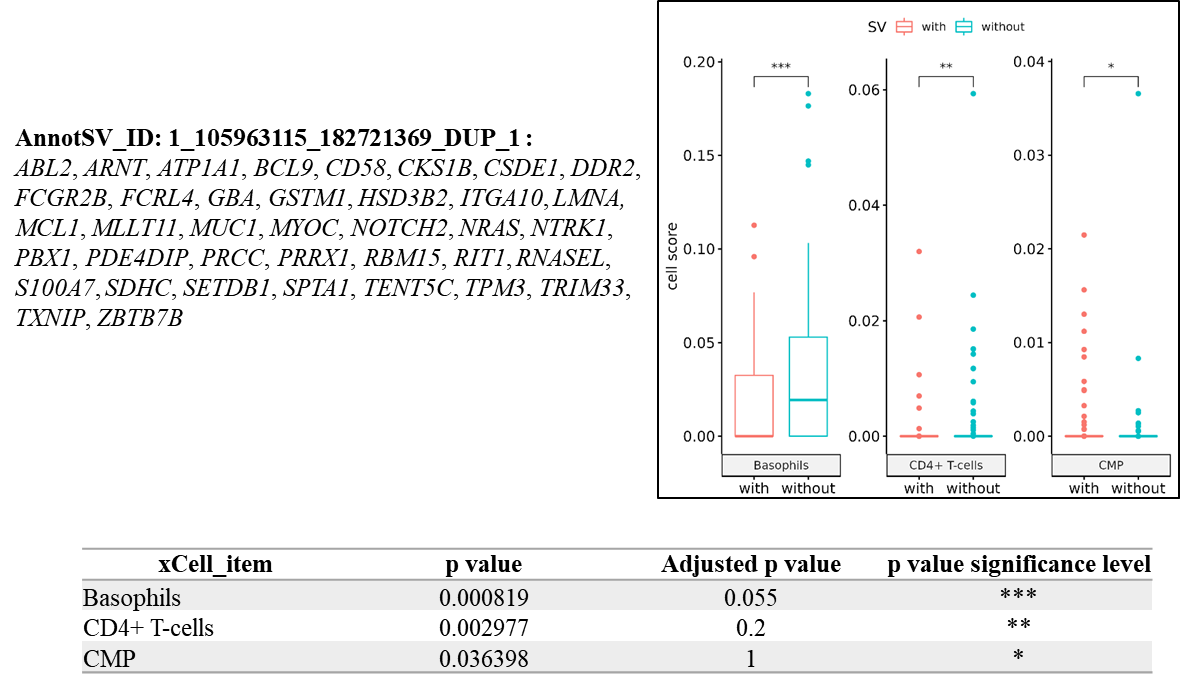


**Fig. S23.**

**Fig. S23. The heatmap for the association between bad survival-related alternative splicing (AS) events and the expression of immune checkpoint genes, as well as the immune and stroma cell types and scores.** The heatmap illustrates the significant differences between higher and lower PSI groups of bad survival-related AS events. The red color indicates that the median cell score or gene expression in the higher PSI group is higher than in the lower PSI group, while blue indicates the opposite. The depth of the color indicates the adjusted p-value, with deeper colors representing lower p values.

**
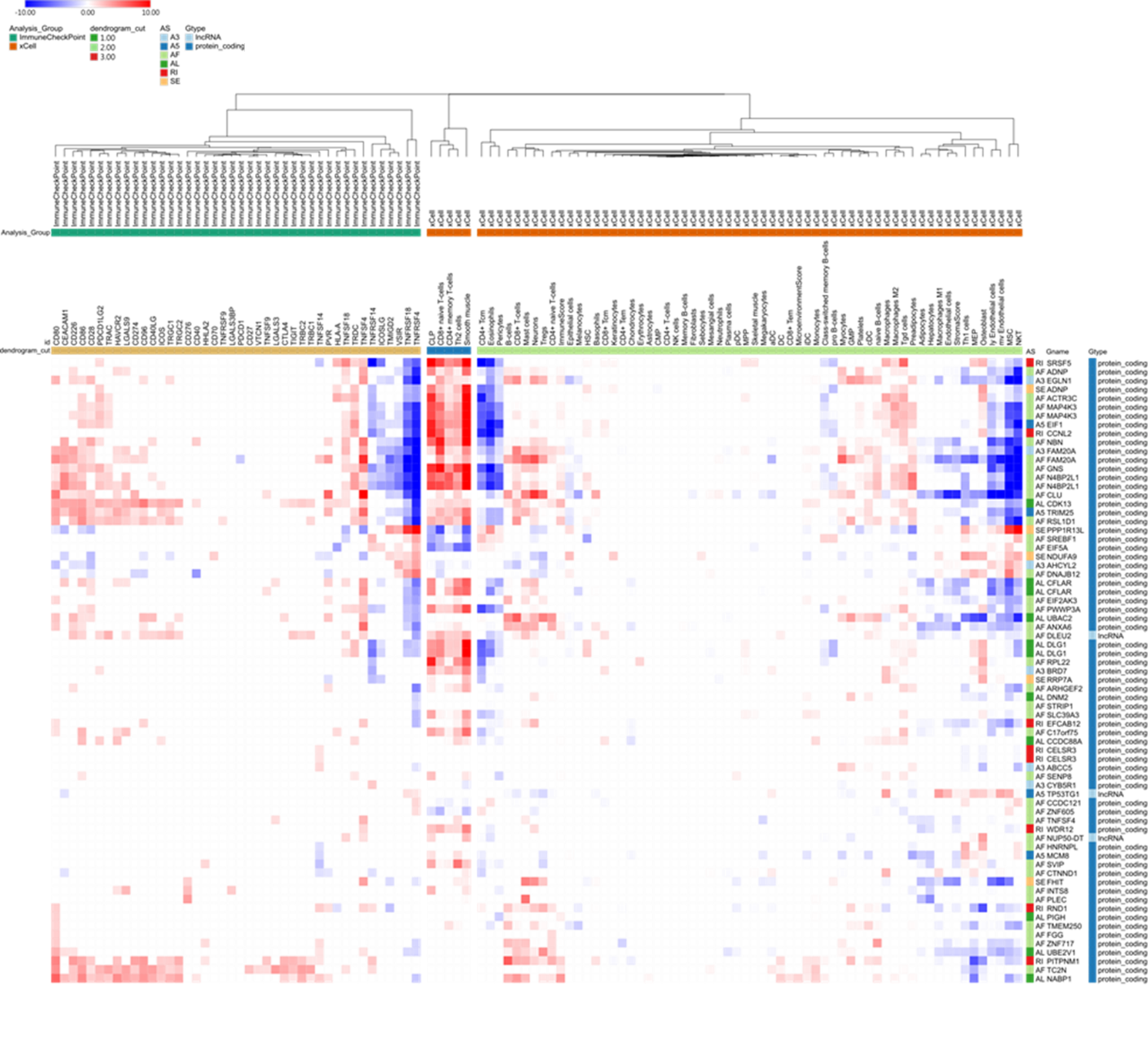
**

**Supplementary tables**

**Table S1**

**Table S1.** The demographic data of 254 Taiwanese HCCs.

| Clinical Variable |  | All Partients n (%) | HBV+ HCV- n (%) | HBV- HCV+ n (%) | HBV+ HCV+ n (%) | HBV- HCV- n (%) | Double Cancer n (%) |
| --- | --- | --- | --- | --- | --- | --- | --- |
| Cohort size |  | 254 ( 100 ) | 106 ( 42 ) | 82 ( 32 ) | 19 ( 7 ) | 28 ( 11 ) | 19 ( 7 ) |
| Gender | Male | 191 ( 75 ) | 84 ( 79 ) | 51 ( 62 ) | 14 ( 74 ) | 25 ( 89 ) | 17 ( 89 ) |
|  | Female | 63 ( 25 ) | 22 ( 21 ) | 31 ( 38 ) | 5 ( 26 ) | 3 ( 11 ) | 2 ( 11 ) |
| Age | Mean | 60 | 55 | 65 | 61 | 58 | 66 |
| ClinicalStage | I | 122 ( 48 ) | 44 ( 42 ) | 41 ( 50 ) | 10 ( 53 ) | 14 ( 50 ) | 13 ( 68 ) |
|  | II | 95 ( 37 ) | 40 ( 38 ) | 31 ( 38 ) | 9 ( 47 ) | 9 ( 32 ) | 6 ( 32 ) |
|  | III | 17 ( 7 ) | 10 ( 9 ) | 6 ( 7 ) | 0 ( 0 ) | 1 ( 4 ) | 0 ( 0 ) |
|  | IIIA | 12 ( 5 ) | 8 ( 8 ) | 2 ( 2 ) | 0 ( 0 ) | 2 ( 7 ) | 0 ( 0 ) |
|  | IIIB | 1 ( 0 ) | 1 ( 1 ) | 0 ( 0 ) | 0 ( 0 ) | 0 ( 0 ) | 0 ( 0 ) |
|  | IIIC | 4 ( 2 ) | 2 ( 2 ) | 1 ( 1 ) | 0 ( 0 ) | 1 ( 4 ) | 0 ( 0 ) |
|  | IVB | 1 ( 0 ) | 0 ( 0 ) | 0 ( 0 ) | 0 ( 0 ) | 1 ( 4 ) | 0 ( 0 ) |
|  | Unknown/Missing | 2 ( 1 ) | 1 ( 1 ) | 1 ( 1 ) | 0 ( 0 ) | 0 ( 0 ) | 0 ( 0 ) |
| Number of tumor | 1 | 185 ( 73 ) | 84 ( 79 ) | 52 ( 63 ) | 15 ( 79 ) | 22 ( 79 ) | 12 ( 63 ) |
|  | 2 | 41 ( 16 ) | 12 ( 11 ) | 19 ( 23 ) | 4 ( 21 ) | 1 ( 4 ) | 5 ( 26 ) |
|  | 3 | 13 ( 5 ) | 5 ( 5 ) | 3 ( 4 ) | 0 ( 0 ) | 3 ( 11 ) | 2 ( 11 ) |
|  | 4 | 4 ( 2 ) | 2 ( 2 ) | 2 ( 2 ) | 0 ( 0 ) | 0 ( 0 ) | 0 ( 0 ) |
|  | 5 | 2 ( 1 ) | 1 ( 1 ) | 1 ( 1 ) | 0 ( 0 ) | 0 ( 0 ) | 0 ( 0 ) |
|  | Multinodular | 9 ( 4 ) | 2 ( 2 ) | 5 ( 6 ) | 0 ( 0 ) | 2 ( 7 ) | 0 ( 0 ) |
|  | Unknown/Missing | 1 ( 0 ) | 0 ( 0 ) | 1 ( 1 ) | 0 ( 0 ) | 0 ( 0 ) | 0 ( 0 ) |
| Tumor size | <3 cm | 79 ( 31 ) | 25 ( 24 ) | 34 ( 41 ) | 7 ( 37 ) | 5 ( 18 ) | 8 ( 42 ) |
|  | >=3 cm | 171 ( 67 ) | 81 ( 76 ) | 46 ( 56 ) | 12 ( 63 ) | 22 ( 79 ) | 10 ( 53 ) |
|  | Unknown/Missing | 4 ( 2 ) | 0 ( 0 ) | 2 ( 2 ) | 0 ( 0 ) | 1 ( 4 ) | 1 ( 5 ) |
| Tumor size | <5 cm | 159 ( 63 ) | 61 ( 58 ) | 63 ( 77 ) | 13 ( 68 ) | 9 ( 32 ) | 13 ( 68 ) |
|  | >=5 cm | 91 ( 36 ) | 45 ( 42 ) | 17 ( 21 ) | 6 ( 32 ) | 18 ( 64 ) | 5 ( 26 ) |
|  | Unknown/Missing | 4 ( 2 ) | 0 ( 0 ) | 2 ( 2 ) | 0 ( 0 ) | 1 ( 4 ) | 1 ( 5 ) |
| Macrovascular invasion | Yes | 76 ( 30 ) | 43 ( 41 ) | 15 ( 18 ) | 8 ( 42 ) | 8 ( 29 ) | 2 ( 11 ) |
|  | No | 173 ( 68 ) | 60 ( 57 ) | 66 ( 80 ) | 11 ( 58 ) | 19 ( 68 ) | 17 ( 89 ) |
|  | Unknown/Missing | 5 ( 2 ) | 3 ( 3 ) | 1 ( 1 ) | 0 ( 0 ) | 1 ( 4 ) | 0 ( 0 ) |
| AST | Normal, 13-39 IU/L | 94 ( 37 ) | 45 ( 42 ) | 16 ( 20 ) | 7 ( 37 ) | 16 ( 57 ) | 10 ( 53 ) |
|  | Abnormal, ≥40 IU/L | 160 ( 63 ) | 61 ( 58 ) | 66 ( 80 ) | 12 ( 63 ) | 12 ( 43 ) | 9 ( 47 ) |
| ALT | Normal, 5-40 IU/L | 105 ( 41 ) | 49 ( 46 ) | 28 ( 34 ) | 6 ( 32 ) | 14 ( 50 ) | 8 ( 42 ) |
|  | Abnormal, ≥41 IU/L | 149 ( 59 ) | 57 ( 54 ) | 54 ( 66 ) | 13 ( 68 ) | 14 ( 50 ) | 11 ( 58 ) |
| AFP | Normal, <9 ng/mL | 59 ( 23 ) | 24 ( 23 ) | 11 ( 13 ) | 5 ( 26 ) | 10 ( 36 ) | 9 ( 47 ) |
|  | Abnormal, ≥9 ng/mL | 195 ( 77 ) | 82 ( 77 ) | 71 ( 87 ) | 14 ( 74 ) | 18 ( 64 ) | 10 ( 53 ) |
| Total bilirubin | Normal, 0.3-1.0 mg/dL | 189 ( 74 ) | 74 ( 70 ) | 65 ( 79 ) | 15 ( 79 ) | 20 ( 71 ) | 15 ( 79 ) |
|  | Abnormal, >1.0 mg/dL | 65 ( 26 ) | 32 ( 30 ) | 17 ( 21 ) | 4 ( 21 ) | 8 ( 29 ) | 4 ( 21 ) |
| Albumin | Normal, 3.5-5.7 g/dL | 103 ( 41 ) | 44 ( 42 ) | 37 ( 45 ) | 6 ( 32 ) | 10 ( 36 ) | 6 ( 32 ) |
|  | Abnormal, <3.5 g/dL | 151 ( 59 ) | 62 ( 58 ) | 45 ( 55 ) | 13 ( 68 ) | 18 ( 64 ) | 13 ( 68 ) |
| Fibrosis score | 0 | 10 ( 4 ) | 6 ( 6 ) | 0 ( 0 ) | 0 ( 0 ) | 4 ( 14 ) | 0 ( 0 ) |
|  | 1 | 41 ( 16 ) | 19 ( 18 ) | 6 ( 7 ) | 2 ( 11 ) | 9 ( 32 ) | 5 ( 26 ) |
|  | 2 | 51 ( 20 ) | 22 ( 21 ) | 18 ( 22 ) | 6 ( 32 ) | 2 ( 7 ) | 3 ( 16 ) |
|  | 3 | 46 ( 18 ) | 20 ( 19 ) | 16 ( 20 ) | 3 ( 16 ) | 1 ( 4 ) | 6 ( 32 ) |
|  | 4 | 69 ( 27 ) | 30 ( 28 ) | 28 ( 34 ) | 5 ( 26 ) | 5 ( 18 ) | 1 ( 5 ) |
|  | Unknown/Missing | 37 ( 15 ) | 9 ( 8 ) | 14 ( 17 ) | 3 ( 16 ) | 7 ( 25 ) | 4 ( 21 ) |
| Cirrhosis | Yes | 157 ( 62 ) | 60 ( 57 ) | 64 ( 78 ) | 12 ( 63 ) | 9 ( 32 ) | 12 ( 63 ) |
|  | No | 97 ( 38 ) | 46 ( 43 ) | 18 ( 22 ) | 7 ( 37 ) | 19 ( 68 ) | 7 ( 37 ) |
| Fatty liver | Yes | 120 ( 47 ) | 47 ( 44 ) | 34 ( 41 ) | 13 ( 68 ) | 13 ( 46 ) | 13 ( 68 ) |
|  | No | 4 ( 2 ) | 3 ( 3 ) | 0 ( 0 ) | 1 ( 5 ) | 0 ( 0 ) | 0 ( 0 ) |
|  | Unknown/Missing | 130 ( 51 ) | 56 ( 53 ) | 48 ( 59 ) | 5 ( 26 ) | 15 ( 54 ) | 6 ( 32 ) |
| Edmondson grade | 1 | 3 ( 1 ) | 1 ( 1 ) | 2 ( 2 ) | 0 ( 0 ) | 0 ( 0 ) | 0 ( 0 ) |
|  | 2 | 135 ( 53 ) | 53 ( 50 ) | 40 ( 49 ) | 12 ( 63 ) | 17 ( 61 ) | 13 ( 68 ) |
|  | 3 | 106 ( 42 ) | 48 ( 45 ) | 38 ( 46 ) | 5 ( 26 ) | 10 ( 36 ) | 5 ( 26 ) |
|  | 4 | 9 ( 4 ) | 4 ( 4 ) | 1 ( 1 ) | 2 ( 11 ) | 1 ( 4 ) | 1 ( 5 ) |
|  | Unknown/Missing | 1 ( 0 ) | 0 ( 0 ) | 1 ( 1 ) | 0 ( 0 ) | 0 ( 0 ) | 0 ( 0 ) |
| BMI | <18.5 | 127 ( 50 ) | 58 ( 55 ) | 42 ( 51 ) | 10 ( 53 ) | 11 ( 39 ) | 6 ( 32 ) |
|  | Normal, 18.5<=BMI<24 | 34 ( 13 ) | 14 ( 13 ) | 10 ( 12 ) | 0 ( 0 ) | 5 ( 18 ) | 5 ( 26 ) |
|  | Abnormal, >=24 | 93 ( 37 ) | 34 ( 32 ) | 30 ( 37 ) | 9 ( 47 ) | 12 ( 43 ) | 8 ( 42 ) |
| Cholesterol Total | Normal, <180 mg/dL | 241 ( 95 ) | 98 ( 92 ) | 82 ( 100 ) | 17 ( 89 ) | 25 ( 89 ) | 19 ( 100 ) |
|  | Abnormal, >=180 mg/dL | 13 ( 5 ) | 8 ( 8 ) | 0 ( 0 ) | 2 ( 11 ) | 3 ( 11 ) | 0 ( 0 ) |
| *TERT* | Wild-type | 134 (53) | 75 (71) | 27 (33) | 10 (53) | 14 (50) | 8 (42) |
|  | Mutation | 120 (47) | 31 (29) | 55 (67) | 9 (47) | 14 (50) | 11 (58) |
| Treatment | OP | 109 (43) | 53 (50) | 26 (32) | 8 (42) | 14 (50) | 8 (42) |
|  | OP/CT | 25 (10) | 8 (8) | 7 (9) | 6 (32) | 3 (11) | 1 (5) |
|  | OP/TACE | 45 (18) | 18 (17) | 19 (23) | 1 (5) | 4 (14) | 3 (16) |
|  | OP/CT/TACE | 6 (2) | 3 (3) | 2 (2) | 1 (5) | 0 (0) | 0 (0) |
|  | OP/Immune drug therapy | 12 (5) | 7 (7) | 2 (2) | 2 (11) | 0 (0) | 1 (5) |
|  | Others | 2 (1) | 1 (1) | 1 (1) | 0 (0) | 0 (0) | 0 (0) |
|  | Transplant | 36 (14) | 12 (11) | 15 (18) | 0 (0) | 6 (21) | 3 (16) |
|  | Unknown/Missing | 19 (7) | 4 (4) | 10 (12) | 1 (5) | 1 (4) | 3 (16) |

OP: Operation, CT: Chemotherapy, TACE: Trans-Arterial Chemo-Embolization

**Table S2**

**Table S2.** The details of mutations of 114 histone-related genes of 254 Taiwanese HCCs in our cohort.

| CHROM | POS | REF | ALT | case_no | Clinvar | GenomAD | TWB | dbSNP_155 | GeneName | CADD | mRNA | Protein |
| --- | --- | --- | --- | --- | --- | --- | --- | --- | --- | --- | --- | --- |
| chr17 | 50168397 | G | A | HCC_526_S | Pathogenic/Likely_pathogenic | 0.000013963 | 6.71E-04 | rs372210292 | *HILS1* | 23 |  |  |
| chr6 | 26055813 | T | A | HCC_951_S | NULL | NULL | NULL | NULL | *HIST1H1C* | 53 | c.616T>A | p.K206* |
| chr17 | 50185563 | A | T | HCC_784_S | NULL | NULL | NULL | NULL | *HILS1* | 45 |  |  |
| chr7 | 151214167 | C | A | HCC_555_S | NULL | NULL | NULL | NULL | *AC021097.2* | 43 |  |  |
| chr6 | 26103996 | A | T | HCC_919_S | NULL | NULL | NULL | NULL | *HIST1H1T* | 42 |  |  |
| chr6 | 26033214 | T | A | HCC_919_S | NULL | NULL | NULL | NULL | *HIST1H2AB* | 42 | c.355T>A | p.K119* |
| chr6 | 26103996 | A | T | HCC_583_S | NULL | NULL | NULL | NULL | *HIST1H4C* | 42 | c.121A>T | p.K17* |
| chr6 | 26020694 | C | T | HCC_933_S | NULL | 0.000160453 | 0.005365526 | rs181930473 | *HIST1H1A* | 41 |  |  |
| chr6 | 26020694 | C | T | HCC_933_S | NULL | 0.000160453 | 0.005365526 | rs181930473 | *HIST1H3A* | 41 | c.205C>T | p.Q69* |
| chr6 | 26020694 | C | T | HCC_933_S | NULL | 0.000160453 | 0.005365526 | rs181930473 | *HIST1H4A* | 41 |  |  |
| chr7 | 151210201 | T | A | HCC_921_S | NULL | NULL | NULL | NULL | *AC021097.2* | 39 | c.178T>A | p.K40* |
| chr10 | 70091799 | A | T | HCC_665_S | NULL | NULL | NULL | NULL | *H2AFY2* | 38 | c.586A>T | p.R108* |
| chr6 | 26216610 | T | A | HCC_946_S | NULL | NULL | NULL | NULL | *AL031777.2* | 37 |  |  |
| chr6 | 26216610 | T | A | HCC_946_S | NULL | NULL | NULL | NULL | *HIST1H2AE* | 37 |  |  |
| chr6 | 26216610 | T | A | HCC_946_S | NULL | NULL | NULL | NULL | *HIST1H2BG* | 37 | c.83T>A | p.K12* |
| chr3 | 129547534 | A | T | HCC_944_S | NULL | NULL | NULL | NULL | *H1FOO* | 36 | c.237A>T | p.K78* |
| chr5 | 135370110 | T | A | HCC_786_S | NULL | NULL | NULL | NULL | *H2AFY* | 36 | c.799T>A | p.R69* |
| chr1 | 149886571 | T | A | HCC_786_S | NULL | NULL | NULL | NULL | *HIST2H2AB* | 36 |  |  |
| chr1 | 149886595 | T | A | HCC_786_S | NULL | NULL | NULL | NULL | *HIST2H2AB* | 36 |  |  |
| chr1 | 149886571 | T | A | HCC_676_S | NULL | NULL | NULL | NULL | *HIST2H2AC* | 36 |  |  |
| chr1 | 149886595 | T | A | HCC_676_S | NULL | NULL | NULL | NULL | *HIST2H2AC* | 36 |  |  |
| chr1 | 149886571 | T | A | HCC_676_S | NULL | NULL | NULL | NULL | *HIST2H2BE* | 36 | c.82T>A | p.K24* |
| chr1 | 149886595 | T | A | HCC_522_S | NULL | NULL | NULL | NULL | *HIST2H2BE* | 36 | c.58T>A | p.K16* |
| chr5 | 17486671 | A | T | HCC_649_S | NULL | NULL | NULL | NULL | *AC106774.4* | 35 |  |  |
| chr2 | 26776480 | A | T | HCC_919_S | NULL | NULL | NULL | NULL | *CENPA* | 33 |  |  |
| chr17 | 50167580 | A | T | HCC_919_S | NULL | NULL | NULL | NULL | *HILS1* | 33 |  |  |
| chr6 | 26156626 | G | C | HCC_655_S | NULL | NULL | NULL | NULL | *HIST1H1E* | 32 | c.273G>C | p.R79P |
| chr6 | 26156626 | G | C | HCC_655_S | NULL | NULL | NULL | NULL | *HIST1H2BD* | 32 |  |  |
| chr1 | 149886514 | A | T | HCC_655_S | NULL | NULL | NULL | NULL | *HIST2H2AB* | 32 |  |  |
| chr1 | 149886514 | A | T | HCC_551_S | NULL | NULL | NULL | NULL | *HIST2H2AC* | 32 |  |  |
| chr1 | 149886514 | A | T | HCC_551_S | NULL | NULL | NULL | NULL | *HIST2H2BE* | 32 | c.139A>T | p.Y43N |
| chr1 | 143905819 | G | A | HCC_874_S | NULL | 4.99871E-05 | 0.003353454 | rs587605296 | *AC239798.4* | 31 |  |  |
| chr6 | 26156625 | C | A | HCC_874_S | NULL | 6.97623E-06 | NULL | rs1481520703 | *HIST1H1E* | 31 | c.272C>A | p.R79S |
| chr6 | 26156625 | C | A | HCC_751_S | NULL | 6.97623E-06 | NULL | rs1481520703 | *HIST1H2BD* | 31 |  |  |
| chr6 | 26188790 | C | A | HCC_776_S | NULL | NULL | NULL | NULL | *HIST1H2BE* | 31 |  |  |
| chr6 | 26188790 | C | A | HCC_776_S | NULL | NULL | NULL | NULL | *HIST1H4D* | 31 | c.287C>A | p.R96L |
| chr17 | 50167690 | T | A | HCC_628_S | NULL | NULL | NULL | NULL | *HILS1* | 30 |  |  |
| chr6 | 26156719 | A | T | HCC_772_S | NULL | NULL | NULL | NULL | *HIST1H1E* | 30 | c.366A>T | p.K110M |
| chr6 | 26156719 | A | T | HCC_772_S | NULL | NULL | NULL | NULL | *HIST1H2BD* | 30 |  |  |

**Table S3**

**Table S3.** The detail of mutations of 74 HCC-related long non-coding RNA genes of 254 Taiwanese HCCs in our cohort.

| CHROM | POS | REF | ALT | case_no | Clinvar | GenomAD | TWB | dbSNP_155 | GeneName | CADD |
| --- | --- | --- | --- | --- | --- | --- | --- | --- | --- | --- |
| chr11 | 9785177 | G | A | HCC_612_S | NULL | NULL | NULL | rs750756174 | *SBF2-AS1* | 42 |
| chr6 | 1.65E+08 | G | C | HCC_925_S | NULL | NULL | NULL | NULL | *LINC473* | 40 |
| chr16 | 54933463 | A | T | HCC_531_S | NULL | NULL | NULL | NULL | *CRNDE* | 39 |
| chr6 | 1.66E+08 | T | A | HCC_869_S | NULL | NULL | NULL | NULL | *LINC473* | 38 |
| chr16 | 54932538 | T | A | HCC_1007_S | NULL | NULL | NULL | NULL | *CRNDE* | 37 |
| chr8 | 1.27E+08 | G | T | HCC_741_S | NULL | 2.09E-05 | 0.001341 | rs536151237 | *PCAT1* | 36 |
| chr14 | 20699756 | A | T | HCC_688_S | NULL | NULL | NULL | NULL | *Lnc-EGFR* | 35 |
| chr5 | 93585484 | A | T | HCC_784_S | NULL | NULL | NULL | NULL | *NR2F1-AS1* | 35 |
| chr2 | 1.76E+08 | C | T | HCC_555_S | NULL | NULL | NULL | NULL | *HAGLR* | 34 |
| chr1 | 32280077 | A | T | HCC_503_S | NULL | NULL | NULL | NULL | *Lnc-Tim3* | 34 |
| chr16 | 67621440 | A | T | HCC_669_S | NULL | NULL | NULL | NULL | *ICR* | 33 |
| chr6 | 1.65E+08 | T | C | HCC_770_S | NULL | NULL | NULL | NULL | *LINC473* | 33 |
| chr1 | 3732796 | G | A | HCC_509_S | NULL | 3.49E-05 | NULL | rs779902863 | *TP73-AS1* | 33 |
| chr5 | 93585360 | A | T | HCC_938_S | NULL | NULL | NULL | NULL | *NR2F1-AS1* | 33 |
| chr2 | 1.76E+08 | C | A | HCC_1009_S, HCC_561_S, HCC_960_S | NULL | 4.19E-05 | 0.005366 | rs764238870 | *HAGLR* | 33 |
| chr7 | 97007830 | A | T | HCC_656_S | NULL | NULL | NULL | NULL | *DLX6-AS1* | 33 |
| chr6 | 1.65E+08 | T | C | HCC_872_S | NULL | NULL | NULL | NULL | *LINC473* | 32 |
| chr1 | 1.51E+08 | A | T | HCC_877_S | NULL | NULL | NULL | NULL | *FALEC* | 32 |
| chr6 | 1.65E+08 | T | C | HCC_924_S | NULL | NULL | NULL | NULL | *LINC473* | 32 |
| chr2 | 1.77E+08 | T | C | HCC_713_S | Uncertain_significance | NULL | NULL | NULL | *NRAL* | 32 |
| chr16 | 54931340 | T | C | HCC_596_S | NULL | NULL | NULL | NULL | *CRNDE* | 32 |
| chr11 | 9780500 | C | T | HCC_552_S | Uncertain_significance | 2.09E-05 | NULL | rs369992460 | *SBF2-AS1* | 32 |
| chr8 | 1.27E+08 | G | T | HCC_626_S | NULL | 6.98E-06 | 0.000671 | rs1410347595 | *PCAT1* | 31 |
| chr2 | 1.76E+08 | A | T | HCC_584_S | NULL | NULL | NULL | NULL | *HAGLR* | 31 |

**Table S4**

**Table S4.** The details of mutations of 36 non-coding driver genes of 254 Taiwanese HCCs in our cohort.

| CHR | POS | REF | ALT | case_no | Clinvar | GenomAD | TWB | dbSNP_155 | GeneName | CADD |
| --- | --- | --- | --- | --- | --- | --- | --- | --- | --- | --- |
| chr19 | 55358317 | T | A | HCC_1008_S | NULL | 3.49E-05 | 0.004024 | rs188366684 | *COX6B2* | 33 |
| chr9 | 35658019 | A | ACGTCCTCAGCTT | HCC_924_S | Likely_pathogenic | 6.98E-06 | NULL | rs1554651400 | *RMRP* | 0.486 |
| chr9 | 35658020 | C | CGTCCTCAGCTTCACAGAGTA | HCC_675_S | Pathogenic/Likely_pathogenic | 7.68E-05 | NULL | rs1554651411 | *RMRP* | 0.409 |
| chr19 | 55352661 | AG | A | HCC_940_S | NULL | NULL | NULL | NULL | *COX6B2* | - |
| chr11 | 65418792 | AG | A | HCC_691_S | NULL | NULL | NULL | NULL | *NEAT1* | - |
| chr11 | 65419609 | G | GA | HCC_767_S | NULL | NULL | NULL | NULL | *NEAT1* | - |
| chr11 | 65422673 | GTC | G | HCC_516_S | NULL | NULL | NULL | NULL | *NEAT1* | - |
| chr11 | 65425449 | TG | T | HCC_894_S | NULL | NULL | NULL | NULL | *NEAT1* | - |
| chr11 | 65426675 | AGATAGAACTG | A | HCC_891_S | NULL | NULL | NULL | NULL | *NEAT1* | - |
| chr11 | 65427756 | CTCT | C | HCC_623_S | NULL | NULL | NULL | NULL | *NEAT1* | - |
| chr11 | 65428267 | A | AG | HCC_880_S | NULL | NULL | NULL | NULL | *NEAT1* | - |
| chr11 | 65428613 | G | GAAATGGC | HCC_896_S | NULL | NULL | NULL | NULL | *NEAT1* | - |
| chr11 | 65429005 | ATT | A | HCC_523_S | NULL | NULL | NULL | NULL | *NEAT1* | - |
| chr11 | 65429041 | AATG | A | HCC_588_S | NULL | NULL | NULL | NULL | *NEAT1* | - |
| chr11 | 65429481 | GGTGTGTGTGTGTGTGCGTGT | G | HCC_970_S | NULL | NULL | NULL | NULL | *NEAT1* | - |
| chr11 | 65430642 | T | TA | HCC_913_S | NULL | NULL | NULL | NULL | *NEAT1* | - |
| chr11 | 65430902 | CATAT | C | HCC_881_S | NULL | NULL | NULL | NULL | *NEAT1* | - |
| chr11 | 65431089 | TGTGA | T | HCC_762_S | NULL | NULL | NULL | NULL | *NEAT1* | - |
| chr11 | 65431251 | ATT | A | HCC_878_S | NULL | NULL | NULL | NULL | *NEAT1* | - |
| chr11 | 65431278 | TC | T | HCC_921_S | NULL | NULL | NULL | NULL | *NEAT1* | - |
| chr11 | 65431458 | T | TG | HCC_997_S | NULL | NULL | NULL | NULL | *NEAT1* | - |
| chr11 | 65431928 | CT | C | HCC_577_S | NULL | NULL | NULL | NULL | *NEAT1* | - |
| chr11 | 65431930 | AATTT | A | HCC_577_S | NULL | NULL | NULL | NULL | *NEAT1* | - |
| chr11 | 65432337 | GCCACTTATGATTGTAAACCT | G | HCC_653_S | NULL | NULL | NULL | NULL | *NEAT1* | - |
| chr11 | 65432646 | TATA | T | HCC_649_S | NULL | NULL | NULL | NULL | *NEAT1* | - |
| chr11 | 65432684 | GT | G | HCC_976_S | NULL | NULL | NULL | NULL | *NEAT1* | - |
| chr11 | 65432771 | AT | A | HCC_923_S | NULL | NULL | NULL | NULL | *NEAT1* | - |
| chr11 | 65432904 | CATAAGTTAGCTCTCACTT | C | HCC_559_S | NULL | NULL | NULL | NULL | *NEAT1* | - |
| chr11 | 65433297 | TAAAATG | T | HCC_934_S | NULL | NULL | NULL | NULL | *NEAT1* | - |
| chr11 | 65433307 | TGG | T | HCC_934_S | NULL | NULL | NULL | NULL | *NEAT1* | - |
| chr11 | 65433310 | AAT | A | HCC_934_S | NULL | NULL | NULL | NULL | *NEAT1* | - |
| chr11 | 65433375 | TAAGGCAAGAATATTCTCTCTGTTGGAACTCA | T | HCC_710_S | NULL | NULL | NULL | NULL | *NEAT1* | - |
| chr11 | 65433687 | ACTT | A | HCC_623_S | NULL | NULL | NULL | NULL | *NEAT1* | - |
| chr11 | 65433809 | GTGAAGTCTGAGATTTTACTACACCT | G | HCC_755_S | NULL | NULL | NULL | NULL | *NEAT1* | - |
| chr11 | 65434104 | T | TC | HCC_926_S | NULL | NULL | NULL | NULL | *NEAT1* | - |
| chr11 | 65434155 | AGTTT | A | HCC_981_S | NULL | NULL | NULL | NULL | *NEAT1* | - |
| chr11 | 65434447 | TACATTATTGATTACTTTTTTTCATTTA | T | HCC_894_S | NULL | NULL | NULL | NULL | *NEAT1* | - |
| chr11 | 65434620 | A | ATG | HCC_951_S | NULL | NULL | NULL | NULL | *NEAT1* | - |
| chr11 | 65434928 | AGCATTCATGGGCTTAAT | A | HCC_782_S | NULL | NULL | NULL | NULL | *NEAT1* | - |
| chr11 | 65435210 | G | GAAT | HCC_599_S | NULL | NULL | NULL | NULL | *NEAT1* | - |
| chr11 | 65436547 | TCA | T | HCC_884_S | NULL | NULL | NULL | NULL | *NEAT1* | - |
| chr11 | 65436762 | ATTAAGTTGTTTCAGC | A | HCC_939_S | NULL | NULL | NULL | NULL | *NEAT1* | - |
| chr11 | 65436852 | TGA | T | HCC_713_S | NULL | NULL | NULL | NULL | *NEAT1* | - |
| chr11 | 65437077 | GATTA | G | HCC_872_S | NULL | NULL | NULL | NULL | *NEAT1* | - |
| chr11 | 65437079 | TTAA | T | HCC_786_S, HCC_777_S | NULL | NULL | NULL | NULL | *NEAT1* | - |
| chr11 | 65437180 | AGTTTATATATATATG | A | HCC_569_S | NULL | NULL | NULL | NULL | *NEAT1* | - |
| chr11 | 65437250 | T | TAC | HCC_985_S | NULL | NULL | NULL | NULL | *NEAT1* | - |
| chr11 | 65438026 | TC | T | HCC_869_S | NULL | NULL | NULL | NULL | *NEAT1* | - |
| chr11 | 65440118 | AAAT | A | HCC_727_S | NULL | NULL | NULL | NULL | *NEAT1* | - |
| chr11 | 65440195 | TTTTATCATCTGATGATGAACTTAATGTTTCCATTTG | T | HCC_938_S | NULL | NULL | NULL | NULL | *NEAT1* | - |
| chr11 | 65440490 | CA | C | HCC_984_S | NULL | NULL | NULL | NULL | *NEAT1* | - |
| chr11 | 65441060 | TAA | T | HCC_569_S | NULL | NULL | NULL | NULL | *NEAT1* | - |
| chr11 | 65441100 | T | TAA | HCC_507_S | NULL | NULL | NULL | NULL | *NEAT1* | - |
| chr11 | 65443951 | G | GT | HCC_972_S | NULL | NULL | NULL | NULL | *NEAT1* | - |
| chr11 | 65445536 | C | CA | HCC_471_S | NULL | NULL | NULL | NULL | *NEAT1* | - |
| chr11 | 65445770 | T | TG | HCC_872_S | NULL | NULL | NULL | NULL | *NEAT1* | - |
| chr11 | 65446796 | GTC | G | HCC_505_S | NULL | NULL | NULL | NULL | *NEAT1* | - |
| chr11 | 65447822 | T | TCTCA | HCC_969_S | NULL | NULL | NULL | NULL | *NEAT1* | - |
| chr11 | 65449233 | G | GT | HCC_555_S | NULL | NULL | NULL | NULL | *NEAT1* | - |
| chr11 | 65498221 | T | TG | HCC_686_S | NULL | NULL | NULL | rs749464304 | *MALAT1* | - |
| chr11 | 65501744 | TATTC | T | HCC_675_S | NULL | NULL | NULL | NULL | *MALAT1* | - |
| chr11 | 65504921 | TGTGGGGATTGGGAACCACTA | T | HCC_726_S | NULL | NULL | NULL | NULL | *MALAT1* | - |
| chr11 | 65505063 | TATGGGACA | T | HCC_556_S | NULL | NULL | NULL | NULL | *MALAT1* | - |
| chr11 | 65506510 | G | GA | HCC_878_S | NULL | NULL | 0.000671 | rs1355113792 | *MALAT1* | - |
| chr11 | 65506660 | T | TA | HCC_599_S | NULL | NULL | NULL | NULL | *MALAT1* | - |

**Table S5**

**Table S5.** The details of genes with differential expression and survival of 254 Taiwanese HCCs. (A) protein-coding genes. (B) non-coding genes.

(A)

| **ensembl_gene_id** | **Gene_name** | **chr.** | **band** | **baseMean** | **log2FoldChange** | **lfcSE** | **padj** | **TPM_mean** | **sur_pvalue** | **High** | **Low** | **good_sur** |
| --- | --- | --- | --- | --- | --- | --- | --- | --- | --- | --- | --- | --- |
| ENSG00000100101 | *Z83844.1* | 22 | q13.1 | 1.507746473 | -2.606250067 | 1.512674 | 0.002503 | 0.006070866 | 0.015899777 | 16 | 238 | High |
| ENSG00000103056 | *SMPD3* | 16 | q22.1 | 106.2407722 | -3.37346077 | 0.320067 | 2.47E-25 | 0.383275591 | 0.019339631 | 68 | 186 | High |
| ENSG00000103710 | *RASL12* | 15 | q22.31 | 62.01948181 | 1.51462524 | 0.207641 | 2.58E-13 | 0.867822835 | 0.03138274 | 83 | 171 | High |
| ENSG00000105173 | *CCNE1* | 19 | q12 | 104.4919934 | 2.428897525 | 0.263795 | 6.54E-20 | 3.953291339 | 0.01728307 | 42 | 212 | High |
| ENSG00000105963 | *ADAP1* | 7 | p22.3 | 112.3428 | -1.523039194 | 0.351167 | 3.88E-06 | 1.897102362 | 0.008392013 | 51 | 203 | High |
| ENSG00000107281 | *NPDC1* | 9 | q34.3 | 269.5051385 | -1.697409636 | 0.380773 | 1.59E-06 | 4.228429134 | 0.042585571 | 85 | 169 | High |
| ENSG00000118137 | *APOA1* | 11 | q23.3 | 84349.83241 | -1.999358086 | 0.32651 | 2.51E-10 | 2015.380638 | 0.014196567 | 104 | 150 | High |
| ENSG00000126705 | *AHDC1* | 1 | p35.3 | 1220.228653 | -1.52906906 | 0.282811 | 2.40E-08 | 6.086370079 | 0.047575176 | 99 | 155 | High |
| ENSG00000130943 | *PKDREJ* | 22 | q13.31 | 13.5040136 | 1.949769585 | 0.366797 | 2.08E-08 | 0.05142126 | 0.047541572 | 66 | 188 | High |
| ENSG00000141449 | *GREB1L* | 18 | q11.1 | 1421.823975 | 1.519365132 | 0.204386 | 9.49E-14 | 9.770279528 | 0.006617562 | 99 | 155 | High |
| ENSG00000146192 | *FGD2* | 6 | p21.2 | 576.5033433 | -1.609635008 | 0.221393 | 2.38E-13 | 6.152677165 | 0.038182893 | 88 | 166 | High |
| ENSG00000146809 | *ASB15* | 7 | q31.32 | 3.20503635 | 2.637833487 | 0.776704 | 4.70E-05 | 0.058035433 | 0.042163382 | 39 | 215 | High |
| ENSG00000148671 | *ADIRF* | 10 | q23.2 | 279.3005236 | -1.793110158 | 0.405835 | 1.65E-06 | 11.03909843 | 0.023858048 | 56 | 198 | High |
| ENSG00000148795 | *CYP17A1* | 10 | q24.32 | 1129.054568 | 4.401650178 | 0.46287 | 9.33E-22 | 24.97533071 | 0.008488754 | 44 | 210 | High |
| ENSG00000149926 | *TLCD3B* | 16 | p11.2 | 13.49393826 | 1.692354976 | 0.409123 | 6.26E-06 | 0.310098425 | 0.020489794 | 62 | 192 | High |
| ENSG00000161243 | *FBXO27* | 19 | q13.2 | 455.9862922 | 1.507030067 | 0.262299 | 4.12E-09 | 10.95554724 | 0.006343572 | 66 | 188 | High |
| ENSG00000163792 | *TCF23* | 2 | p23.3 | 4.630360346 | 1.641645469 | 0.463172 | 6.61E-05 | 0.027586614 | 0.00441028 | 74 | 180 | High |
| ENSG00000179772 | *FOXS1* | 20 | q11.21 | 11.33192469 | 1.552904734 | 0.460464 | 0.000144 | 0.243212598 | 0.036966195 | 97 | 157 | High |
| ENSG00000183186 | *C2CD4C* | 19 | p13.3 | 15.20393715 | -1.926927555 | 0.468495 | 5.15E-06 | 0.094114173 | 0.01197674 | 80 | 174 | High |
| ENSG00000186766 | *FOXI2* | 10 | q26.2 | 1.872327298 | 1.52233721 | 0.673153 | 0.003938 | 0.017814961 | 0.015939978 | 74 | 180 | High |
| ENSG00000196787 | *H2AC11* | 6 | p22.1 | 677.4785904 | 1.815826643 | 0.295888 | 2.49E-10 | 7.76011811 | 0.047739948 | 87 | 167 | High |
| ENSG00000197408 | *CYP2B6* | 19 | q13.2 | 14030.80091 | -2.452277069 | 0.350894 | 1.55E-12 | 84.26816142 | 0.021932619 | 82 | 172 | High |
| ENSG00000204653 | *ASPDH* | 19 | q13.33 | 731.4559946 | -1.865663746 | 0.42334 | 1.60E-06 | 15.89166142 | 0.019223485 | 92 | 162 | High |
| ENSG00000205116 | *TMEM88B* | 1 | p36.33 | 6.369562512 | 2.060715861 | 1.142354 | 0.004476 | 0.395251969 | 0.000590043 | 44 | 210 | High |
| ENSG00000221818 | *EBF2* | 8 | p21.2 | 66.09701178 | 3.146202197 | 0.272154 | 6.41E-30 | 0.40092126 | 0.022268852 | 89 | 165 | High |
| ENSG00000244234 | *GMCL2* | 5 | q35.3 | 10.05666262 | 3.623266715 | 0.628012 | 1.54E-09 | 0.302464567 | 0.03396424 | 21 | 233 | High |
| ENSG00000244242 | *IFITM10* | 11 | p15.5 | 223.0901005 | -2.163719708 | 0.377629 | 2.35E-09 | 2.013547244 | 0.011860101 | 55 | 199 | High |
| ENSG00000274286 | *ADRA2B* | 2 | q11.2 | 87.95984754 | -2.444475752 | 0.271292 | 3.63E-19 | 0.423700787 | 0.019206114 | 93 | 161 | High |
| ENSG00000005073 | *HOXA11* | 7 | p15.2 | 13.29814817 | 5.34937529 | 0.820507 | 6.32E-12 | 0.210622047 | 3.70E-05 | 38 | 216 | Low |
| ENSG00000007350 | *TKTL1* | X | q28 | 26.53005576 | 2.834515009 | 0.781419 | 2.04E-05 | 2.863515748 | 0.012506346 | 11 | 243 | Low |
| ENSG00000007372 | *PAX6* | 11 | p13 | 32.15359233 | 5.143558988 | 0.763212 | 1.51E-12 | 0.45992126 | 0.026774369 | 36 | 218 | Low |
| ENSG00000043355 | *ZIC2* | 13 | q32.3 | 223.2373191 | 5.06973402 | 0.421482 | 3.15E-33 | 3.692377953 | 0.027700827 | 78 | 176 | Low |
| ENSG00000051341 | *POLQ* | 3 | q13.33 | 496.3520577 | 2.875671138 | 0.24281 | 2.89E-31 | 1.876094488 | 0.019371894 | 86 | 168 | Low |
| ENSG00000066405 | *CLDN18* | 3 | q22.3 | 9.952591991 | 4.102977575 | 1.055983 | 3.61E-06 | 0.795858268 | 0.003191612 | 8 | 246 | Low |
| ENSG00000073756 | *PTGS2* | 1 | q31.1 | 130.1070657 | -2.004117882 | 0.348755 | 2.10E-09 | 0.723338583 | 0.011449574 | 53 | 201 | Low |
| ENSG00000074410 | *CA12* | 15 | q22.2 | 491.1663169 | 2.566152981 | 0.44611 | 2.51E-09 | 4.922716535 | 0.046636274 | 47 | 207 | Low |
| ENSG00000079112 | *CDH17* | 8 | q22.1 | 31.62469517 | 2.590872645 | 0.79065 | 6.92E-05 | 1.291720472 | 2.21E-05 | 15 | 239 | Low |
| ENSG00000090382 | *LYZ* | 12 | q15 | 10614.06098 | 2.930777262 | 0.385054 | 2.75E-14 | 220.3297913 | 0.044803189 | 52 | 202 | Low |
| ENSG00000090402 | *SI* | 3 | q26.1 | 4.553531748 | 3.158891477 | 1.284652 | 0.000355 | 0.252866142 | 0.024987451 | 10 | 244 | Low |
| ENSG00000091651 | *ORC6* | 16 | q11.2 | 173.887856 | 1.757344995 | 0.232162 | 2.22E-14 | 3.636224409 | 0.014237692 | 80 | 174 | Low |
| ENSG00000100362 | *PVALB* | 22 | q12.3 | 1.409622476 | -3.013398824 | 0.608191 | 1.27E-07 | 0.052377953 | 0.029946443 | 47 | 207 | Low |
| ENSG00000100604 | *CHGA* | 14 | q32.12 | 3.091395783 | 1.992652104 | 0.888301 | 0.001956 | 0.219720472 | 0.029259558 | 22 | 232 | Low |
| ENSG00000103313 | *MEFV* | 16 | p13.3 | 71.08435325 | -1.6047396 | 0.209556 | 1.51E-14 | 0.51984252 | 0.03918162 | 74 | 180 | Low |
| ENSG00000105509 | *HAS1* | 19 | q13.41 | 5.015168554 | -2.410855117 | 0.750719 | 9.40E-05 | 0.061358268 | 0.020883582 | 42 | 212 | Low |
| ENSG00000105523 | *FAM83E* | 19 | q13.33 | 4.200212672 | -3.305328897 | 0.529155 | 1.52E-10 | 0.057015748 | 0.047176893 | 56 | 198 | Low |
| ENSG00000105825 | *TFPI2* | 7 | q21.3 | 88.33070676 | -1.614545848 | 0.36661 | 2.35E-06 | 1.226232283 | 0.021773723 | 35 | 219 | Low |
| ENSG00000106366 | *SERPINE1* | 7 | q22.1 | 8518.367766 | -2.121605029 | 0.294341 | 2.79E-13 | 47.16865748 | 0.049081611 | 85 | 169 | Low |
| ENSG00000110881 | *ASIC1* | 12 | q13.12 | 84.72415482 | 2.594642648 | 0.376859 | 3.36E-12 | 0.913472441 | 0.026256563 | 68 | 186 | Low |
| ENSG00000112852 | *PCDHB2* | 5 | q31.3 | 73.54496553 | 2.035111247 | 0.442979 | 6.33E-07 | 0.609169291 | 0.000715949 | 51 | 203 | Low |
| ENSG00000113205 | *PCDHB3* | 5 | q31.3 | 80.35535176 | 1.661921692 | 0.408144 | 8.61E-06 | 0.834846457 | 0.02074369 | 42 | 212 | Low |
| ENSG00000113327 | *GABRG2* | 5 | q34 | 11.00487878 | 4.385463638 | 0.9108 | 7.76E-08 | 0.219712598 | 0.044613179 | 31 | 223 | Low |
| ENSG00000115507 | *OTX1* | 2 | p15 | 45.8803584 | 2.669604033 | 0.40207 | 1.63E-11 | 0.659476378 | 0.011957084 | 64 | 190 | Low |
| ENSG00000118785 | *SPP1* | 4 | q22.1 | 8102.712078 | 3.001107442 | 0.439739 | 5.36E-12 | 195.0535118 | 0.049704685 | 63 | 191 | Low |
| ENSG00000119535 | *CSF3R* | 1 | p34.3 | 360.8830104 | -1.530194818 | 0.276964 | 1.28E-08 | 3.563417323 | 0.002388749 | 66 | 188 | Low |
| ENSG00000120322 | *PCDHB8* | 5 | q31.3 | 40.70060432 | 1.63943785 | 0.467835 | 7.65E-05 | 0.498685039 | 0.000708615 | 42 | 212 | Low |
| ENSG00000123485 | *HJURP* | 2 | q37.1 | 323.5309734 | 3.376167111 | 0.239865 | 1.75E-43 | 4.408728346 | 0.028247316 | 85 | 169 | Low |
| ENSG00000125820 | *NKX2-2* | 20 | p11.22 | 5.233367909 | 2.514705895 | 1.446511 | 0.002738 | 0.234240157 | 0.000762709 | 17 | 237 | Low |
| ENSG00000126778 | *SIX1* | 14 | q23.1 | 46.46071472 | 2.945218107 | 0.476843 | 2.65E-10 | 0.493141732 | 0.015593673 | 62 | 192 | Low |
| ENSG00000126787 | *DLGAP5* | 14 | q22.3 | 290.8892967 | 3.285787975 | 0.260673 | 2.97E-35 | 3.929728346 | 0.038114213 | 78 | 176 | Low |
| ENSG00000126838 | *PZP* | 12 | p13.31 | 546.8401164 | -1.876527357 | 0.549867 | 7.39E-05 | 2.495783465 | 0.006405158 | 26 | 228 | Low |
| ENSG00000128683 | *GAD1* | 2 | q31.1 | 40.81307852 | 2.108224429 | 0.497241 | 2.77E-06 | 0.644787402 | 0.016177854 | 48 | 206 | Low |
| ENSG00000133710 | *SPINK5* | 5 | q32 | 59.82317872 | 3.630579349 | 0.459877 | 1.98E-15 | 0.622114173 | 0.038714761 | 64 | 190 | Low |
| ENSG00000135144 | *DTX1* | 12 | q24.13 | 1170.427387 | -2.673271426 | 0.376449 | 8.57E-13 | 7.395086614 | 0.027471841 | 68 | 186 | Low |
| ENSG00000136231 | *IGF2BP3* | 7 | p15.3 | 836.6433424 | 4.90429912 | 0.389193 | 3.37E-36 | 8.655799213 | 0.004541269 | 78 | 176 | Low |
| ENSG00000139800 | *ZIC5* | 13 | q32.3 | 132.2744494 | 5.680379096 | 0.502492 | 1.86E-29 | 1.223448819 | 0.020259764 | 63 | 191 | Low |
| ENSG00000142405 | *NLRP12* | 19 | q13.42 | 12.23397852 | -2.012996328 | 0.304466 | 1.30E-11 | 0.065318898 | 0.005288387 | 83 | 171 | Low |
| ENSG00000145386 | *CCNA2* | 4 | q27 | 551.1013194 | 2.926787455 | 0.28072 | 1.03E-24 | 6.284 | 0.047790293 | 66 | 188 | Low |
| ENSG00000147206 | *NXF3* | X | q22.1 | 26.88057995 | -2.930677647 | 0.457686 | 7.21E-11 | 0.462251969 | 0.024794799 | 33 | 221 | Low |
| ENSG00000147509 | *RGS20* | 8 | q11.23 | 2.626039571 | 2.316566462 | 0.829658 | 0.000337 | 0.115224409 | 7.63E-06 | 47 | 207 | Low |
| ENSG00000149948 | *HMGA2* | 12 | q14.3 | 78.0560869 | 4.239574374 | 0.714637 | 3.09E-10 | 2.29234252 | 0.003552479 | 24 | 230 | Low |
| ENSG00000154025 | *SLC5A10* | 17 | p11.2 | 14.03207265 | 1.524902057 | 0.471487 | 0.000243 | 0.383751969 | 0.039047141 | 33 | 221 | Low |
| ENSG00000159184 | *HOXB13* | 17 | q21.32 | 7.413585171 | 3.290851675 | 1.186972 | 0.000156 | 0.370366142 | 0.001494618 | 18 | 236 | Low |
| ENSG00000160932 | *LY6E* | 8 | q24.3 | 2925.799924 | -2.549914271 | 0.361069 | 1.03E-12 | 52.64826772 | 0.029532497 | 78 | 176 | Low |
| ENSG00000162415 | *ZSWIM5* | 1 | p34.1 | 504.7192346 | 2.378923151 | 0.276688 | 1.05E-17 | 2.685110236 | 0.011426248 | 99 | 155 | Low |
| ENSG00000163092 | *XIRP2* | 2 | q24.3 | 3.683917201 | 1.760160663 | 0.925611 | 0.005808 | 0.032291339 | 0.00684016 | 24 | 230 | Low |
| ENSG00000163464 | *CXCR1* | 2 | q35 | 17.59795704 | -1.615869424 | 0.34351 | 6.18E-07 | 0.133661417 | 0.000180146 | 71 | 183 | Low |
| ENSG00000163577 | *EIF5A2* | 3 | q26.2 | 493.905445 | 1.802620545 | 0.309103 | 1.45E-09 | 2.983141732 | 0.039160958 | 82 | 172 | Low |
| ENSG00000163736 | *PPBP* | 4 | q13.3 | 17.98493728 | -2.366137815 | 0.43153 | 9.33E-09 | 0.233700787 | 0.020948946 | 51 | 203 | Low |
| ENSG00000164841 | *TMEM74* | 8 | q23.1 | 176.1711883 | 3.03007649 | 0.313395 | 1.40E-21 | 0.803448819 | 0.044263531 | 74 | 180 | Low |
| ENSG00000165449 | *SLC16A9* | 10 | q21.2 | 319.9048355 | 2.250711184 | 0.515052 | 1.58E-06 | 2.957401575 | 0.033601675 | 50 | 204 | Low |
| ENSG00000166845 | *C18orf54* | 18 | q21.2 | 155.023222 | 1.513508684 | 0.218185 | 2.91E-12 | 1.018244094 | 0.014431173 | 77 | 177 | Low |
| ENSG00000167757 | *KLK11* | 19 | q13.41 | 2.814546358 | -3.257769504 | 1.059968 | 7.56E-05 | 0.035141732 | 0.023843612 | 23 | 231 | Low |
| ENSG00000168995 | *SIGLEC7* | 19 | q13.41 | 64.8530715 | -1.531457269 | 0.205987 | 9.11E-14 | 1.016834646 | 0.033923316 | 84 | 170 | Low |
| ENSG00000170417 | *TMEM182* | 2 | q12.1 | 210.728198 | 1.58160012 | 0.279812 | 5.64E-09 | 2.805929134 | 0.021895069 | 61 | 193 | Low |
| ENSG00000170703 | *TTLL6* | 17 | q21.32 | 31.84138536 | 2.001179573 | 0.433532 | 5.83E-07 | 0.418976378 | 0.038687409 | 37 | 217 | Low |
| ENSG00000170956 | *CEACAM3* | 19 | q13.2 | 4.584687984 | -1.635002198 | 0.387432 | 4.93E-06 | 0.106192913 | 0.001615715 | 74 | 180 | Low |
| ENSG00000171033 | *PKIA* | 8 | q21.13 | 57.73019375 | 1.961485661 | 0.484224 | 6.42E-06 | 0.725216535 | 0.001011193 | 40 | 214 | Low |
| ENSG00000171049 | *FPR2* | 19 | q13.41 | 26.10694268 | -1.608267243 | 0.323276 | 1.76E-07 | 0.299740157 | 0.048689012 | 70 | 184 | Low |
| ENSG00000171431 | *KRT20* | 17 | q21.2 | 22.64320223 | 5.303271258 | 0.96497 | 2.12E-09 | 1.80696063 | 0.030083694 | 17 | 237 | Low |
| ENSG00000172350 | *ABCG4* | 11 | q23.3 | 5.436714826 | -1.766471878 | 0.304848 | 1.85E-09 | 0.041590551 | 0.036051131 | 77 | 177 | Low |
| ENSG00000172986 | *GXYLT2* | 3 | p13 | 135.6836483 | 1.935996662 | 0.419064 | 5.94E-07 | 1.402531496 | 0.047675001 | 60 | 194 | Low |
| ENSG00000173391 | *OLR1* | 12 | p13.2 | 91.5345435 | 1.757189357 | 0.484311 | 4.03E-05 | 1.444732283 | 0.011264149 | 50 | 204 | Low |
| ENSG00000178460 | *MCMDC2* | 8 | q13.1 | 136.4295649 | 2.197876903 | 0.212037 | 1.06E-24 | 1.477354331 | 0.003752706 | 89 | 165 | Low |
| ENSG00000180318 | *ALX1* | 12 | q21.31 | 4.593943272 | 2.393196667 | 1.488729 | 0.00387 | 0.287291339 | 5.37E-06 | 25 | 229 | Low |
| ENSG00000181085 | *MAPK15* | 8 | q24.3 | 8.20013436 | -1.876178535 | 0.571462 | 0.000115 | 0.478913386 | 0.010273225 | 33 | 221 | Low |
| ENSG00000181617 | *FDCSP* | 4 | q13.3 | 29.57812302 | 1.698636054 | 0.818836 | 0.004435 | 17.43990945 | 0.000615891 | 12 | 242 | Low |
| ENSG00000181690 | *PLAG1* | 8 | q12.1 | 375.2180021 | 2.091682137 | 0.302972 | 2.07E-12 | 2.13130315 | 0.024023174 | 79 | 175 | Low |
| ENSG00000181751 | *MACIR* | 5 | q21.1 | 150.0615026 | 1.769475218 | 0.297592 | 7.85E-10 | 1.690940945 | 0.018860974 | 74 | 180 | Low |
| ENSG00000184363 | *PKP3* | 11 | p15.5 | 34.05834938 | -1.665052222 | 0.727384 | 0.002829 | 0.497177165 | 0.004015198 | 25 | 229 | Low |
| ENSG00000184956 | *MUC6* | 11 | p15.5 | 532.8301678 | -3.119315864 | 0.799483 | 7.13E-06 | 2.465629921 | 0.019984373 | 14 | 240 | Low |
| ENSG00000185002 | *RFX6* | 6 | q22.1 | 6.958780306 | 3.568002034 | 0.8561 | 2.05E-06 | 0.19323622 | 0.04829418 | 19 | 235 | Low |
| ENSG00000185046 | *ANKS1B* | 12 | q23.1 | 504.9232251 | 1.537868411 | 0.405295 | 3.29E-05 | 8.406374016 | 0.019525465 | 62 | 192 | Low |
| ENSG00000187537 | *POTEG* | 14 | q11.2 | 12.00220732 | 4.997766574 | 1.158711 | 4.89E-07 | 0.445330709 | 0.045848031 | 26 | 228 | Low |
| ENSG00000187908 | *DMBT1* | 10 | q26.13 | 30.39484685 | 1.515772877 | 0.599545 | 0.002028 | 0.907724409 | 0.005534753 | 18 | 236 | Low |
| ENSG00000189108 | *IL1RAPL2* | X | q22.3 | 46.5425118 | -2.090433286 | 0.446185 | 4.17E-07 | 0.319625984 | 0.026981863 | 66 | 188 | Low |
| ENSG00000196091 | *MYBPC1* | 12 | q23.2 | 11.51562336 | 2.247431782 | 0.637641 | 3.77E-05 | 3.086677165 | 5.36E-07 | 7 | 247 | Low |
| ENSG00000197123 | *ZNF679* | 7 | q11.21 | 5.229479686 | 3.413975233 | 1.330387 | 0.000225 | 0.200700787 | 0.024611842 | 31 | 223 | Low |
| ENSG00000198062 | *POTEH* | 22 | q11.1 | 9.593192616 | 2.996875675 | 1.040773 | 0.000153 | 0.370751969 | 0.009266821 | 25 | 229 | Low |
| ENSG00000198648 | *STK39* | 2 | q24.3 | 426.6781117 | 1.573813342 | 0.250139 | 1.48E-10 | 4.264440945 | 0.034909895 | 84 | 170 | Low |
| ENSG00000198758 | *EPS8L3* | 1 | p13.3 | 271.4129557 | 3.00642211 | 0.434503 | 2.94E-12 | 7.939964567 | 0.008714072 | 68 | 186 | Low |
| ENSG00000203877 | *RIPPLY2* | 6 | q14.2 | 3.625867419 | 2.965329115 | 1.187727 | 0.000391 | 0.170141732 | 0.022504802 | 28 | 226 | Low |
| ENSG00000204614 | *TRIM40* | 6 | p22.1 | 61.62231847 | -1.528005579 | 0.553379 | 0.001038 | 0.884811024 | 0.003345882 | 38 | 216 | Low |
| ENSG00000206047 | *DEFA1* | 8 | p23.1 | 5.707446564 | -2.75095322 | 0.721099 | 1.15E-05 | 0.219165354 | 0.000549624 | 46 | 208 | Low |
| ENSG00000206262 | *FOXL2NB* | 3 | q22.3 | 1.89157305 | 1.691320956 | 0.858309 | 0.005635 | 0.020755906 | 0.008648037 | 46 | 208 | Low |
| ENSG00000206557 | *TRIM71* | 3 | p22.3 | 1032.312012 | 5.445452748 | 0.564239 | 2.33E-22 | 4.051007874 | 0.017648295 | 58 | 196 | Low |
| ENSG00000221986 | *MYBPHL* | 1 | p13.3 | 2.440212363 | 1.743187834 | 0.892973 | 0.005389 | 0.123574803 | 0.015566349 | 41 | 213 | Low |
| ENSG00000235162 | *C12orf75* | 12 | q23.3 | 275.9318846 | 2.239183472 | 0.319387 | 1.13E-12 | 11.59794094 | 0.023127001 | 61 | 193 | Low |
| ENSG00000253293 | *HOXA10* | 7 | p15.2 | 123.8477472 | 4.569631428 | 0.562573 | 9.53E-17 | 2.269543307 | 0.023156775 | 64 | 190 | Low |
| ENSG00000257008 | *GPR142* | 17 | q25.1 | 1.19771666 | -2.069204955 | 0.644974 | 0.000121 | 0.024377953 | 0.017982439 | 46 | 208 | Low |
| ENSG00000257743 | *MGAM2* | 7 | q34 | 399.1492159 | 3.229068481 | 0.529431 | 3.67E-10 | 1.63234252 | 0.023180951 | 38 | 216 | Low |
| ENSG00000263513 | *FAM72C* | 1 | q21.1 | 66.5528482 | 3.767251403 | 0.352448 | 3.04E-26 | 1.044759843 | 0.034554319 | 62 | 192 | Low |
| ENSG00000285566 | *AL445238.1* | 13 | q21.31 | 3.888879855 | 3.693660276 | 0.825579 | 5.83E-07 | 0.118984252 | 0.00153035 | 48 | 206 | Low |
| ENSG00000287856 | *AL445524.2* | 1 | q42.2 | 26.33697715 | 23.72406476 | 1.927498 | 1.47E-32 | 0.459988189 | 0.017027538 | 29 | 225 | Low |

(B) *: novel transcripts

| ensembl_gene_id | Gene_name | chr. | band | baseMean | log2FoldChange | lfcSE | padj | TPM_mean | sur_pvalue | High | Low | good_sur |
| --- | --- | --- | --- | --- | --- | --- | --- | --- | --- | --- | --- | --- |
| ENSG00000189366 | *ALG1L* | 3 | q21.2 | 61.68134355 | 3.330603161 | 0.337122 | 1.61E-22 | 2.648547244 | 0.033119072 | 84 | 170 | High |
| ENSG00000205890 | **AC108134.1* | 16 | p13.3 | 1.385201781 | -3.593164527 | 0.82434 | 9.91E-07 | 0.004267717 | 0.031936477 | 36 | 218 | High |
| ENSG00000224004 | *ATP5F1CP1* | 14 | q22.2 | 1.980980017 | 2.363611016 | 0.967547 | 0.000794 | 0.10257874 | 0.046831499 | 34 | 220 | High |
| ENSG00000225522 | **AL592114.2* | 1 | q32.1 | 5.093624472 | 1.900951578 | 0.702634 | 0.000658 | 0.46792126 | 0.002405435 | 43 | 211 | High |
| ENSG00000226580 | *RPL39P40* | 21 | q21.1 | 2.768806604 | 1.659260513 | 0.573377 | 0.000544 | 2.801216535 | 0.015227941 | 72 | 182 | High |
| ENSG00000226622 | **AC092155.1* | 2 | p15 | 7.551861071 | -1.929616002 | 0.432739 | 1.21E-06 | 0.102968504 | 0.042162377 | 56 | 198 | High |
| ENSG00000228314 | *CYP4F29P* | 21 | q11.2 | 9.120525983 | -1.550773309 | 0.489832 | 0.000287 | 0.224992126 | 0.008485841 | 54 | 200 | High |
| ENSG00000230918 | *DPP4-DT* | 2 | q24.2 | 9.752923435 | 1.607078395 | 0.382524 | 5.64E-06 | 0.424559055 | 0.045441141 | 65 | 189 | High |
| ENSG00000233485 | *FHAD1-AS1* | 1 | p36.21 | 4.377462952 | 1.616776586 | 0.649049 | 0.001837 | 0.098145669 | 0.030092709 | 27 | 227 | High |
| ENSG00000235774 | **AC023347.1* | 2 | q14.3 | 2.808063102 | 2.857565732 | 1.029062 | 0.000223 | 0.143192913 | 0.044230354 | 57 | 197 | High |
| ENSG00000241158 | *ADAMTS9-AS1* | 3 | p14.1 | 7.849807694 | -1.846574555 | 0.375828 | 1.65E-07 | 0.154822835 | 0.042418127 | 56 | 198 | High |
| ENSG00000247735 | *AC120114.1* | 16 | p11.2 | 34.86906901 | 1.545282171 | 0.197558 | 5.19E-15 | 0.565192913 | 0.005797991 | 86 | 168 | High |
| ENSG00000248431 | **AC021134.1* | 4 | q32.2 | 19.15867855 | 23.21792242 | 1.041044 | 9.95E-107 | 1.215913386 | 0.019502858 | 31 | 223 | High |
| ENSG00000248774 | **AC097534.1* | 4 | q34.1 | 2.95508576 | 1.829583662 | 0.53353 | 7.39E-05 | 0.239200787 | 0.021880891 | 79 | 175 | High |
| ENSG00000248886 | *UGT2A3P7* | 4 | q13.3 | 12.85487697 | 1.954612822 | 0.400499 | 1.78E-07 | 0.623708661 | 0.004161897 | 79 | 175 | High |
| ENSG00000250358 | *LINC02200* | 5 | q22.2 | 12.80378035 | 4.207757651 | 0.928519 | 2.98E-07 | 0.45361811 | 0.009210374 | 38 | 216 | High |
| ENSG00000253931 | *AC105118.1* | 8 | q24.3 | 5.120400001 | 3.868679015 | 0.941719 | 1.98E-06 | 2.464933071 | 0.03244947 | 15 | 239 | High |
| ENSG00000254739 | **AC137894.1* | 11 | p15.5 | 6.733287943 | -2.236299928 | 0.401285 | 5.57E-09 | 0.294110236 | 0.008507505 | 75 | 179 | High |
| ENSG00000255121 | *CCDC84-DT* | 11 | q23.3 | 59.03422731 | 1.666600927 | 0.191733 | 4.01E-18 | 1.707799213 | 0.022853261 | 76 | 178 | High |
| ENSG00000259416 | *LINC02883* | 15 | q25.3 | 28.7293589 | -1.706023146 | 0.287841 | 9.53E-10 | 0.839503937 | 0.028965031 | 80 | 174 | High |
| ENSG00000260874 | **AC106820.5* | 16 | p13.3 | 7.555099361 | 2.655980259 | 0.375227 | 9.91E-13 | 0.413704724 | 0.002988339 | 92 | 162 | High |
| ENSG00000260992 | *DOCK9-DT* | 13 | q32.3 | 6.060216317 | 2.294967036 | 0.366501 | 1.27E-10 | 0.270633858 | 0.04251149 | 90 | 164 | High |
| ENSG00000261172 | **AC133919.2* | 16 | q24.3 | 23.04304827 | 4.237012449 | 0.65079 | 1.03E-11 | 1.634043307 | 0.014098365 | 69 | 185 | High |
| ENSG00000261481 | *AC022167.4* | 16 | p13.2 | 2.306112337 | -2.558411922 | 0.663492 | 1.07E-05 | 0.074925197 | 0.018006881 | 54 | 200 | High |
| ENSG00000273828 | **AL133227.1* | 20 | q13.12 | 7.60648637 | 1.539168677 | 0.359194 | 4.65E-06 | 0.057614173 | 0.049236975 | 74 | 180 | High |
| ENSG00000280187 | *AC022107.1* | 5 | q13.2 | 61.05439533 | 1.820556235 | 0.178534 | 4.37E-24 | 0.543283465 | 0.03081382 | 106 | 148 | High |
| ENSG00000280767 | **AL732314.4* | X | p22.33 | 1.376110753 | -1.515231856 | 0.471796 | 0.000268 | 0.078555118 | 0.037891626 | 66 | 188 | High |
| ENSG00000281189 | *GHET1* | 7 | q36.1 | 14.80125605 | 2.391680255 | 0.283988 | 4.48E-17 | 0.23134252 | 0.042782101 | 95 | 159 | High |
| ENSG00000286271 | **AC008945.2* | 5 | p12 | 23.17177036 | 3.011974261 | 0.438832 | 4.20E-12 | 0.490496063 | 0.04681313 | 75 | 179 | High |
| ENSG00000287024 | **AL022162.1* | X | q26.1 | 6.345731178 | 1.565561984 | 0.427929 | 5.13E-05 | 0.913295276 | 0.026999484 | 88 | 166 | High |
| ENSG00000287712 | **AC005865.2* | 12 | p13.32 | 4.236407148 | -2.523317673 | 0.556704 | 8.01E-07 | 0.07007874 | 0.04685042 | 56 | 198 | High |
| ENSG00000287729 | **AF241728.2* | X | p11.4 | 5.605540356 | 2.331730832 | 0.42058 | 6.74E-09 | 0.147527559 | 0.015111108 | 79 | 175 | High |
| ENSG00000288095 | **AC005618.4* | 5 | q31.3 | 9.409460149 | 2.10100477 | 0.356103 | 9.06E-10 | 0.210826772 | 0.029797891 | 93 | 161 | High |
| ENSG00000250420 | *AACSP1* | 5 | q35.3 | 7.595484049 | 4.370286239 | 1.094881 | 2.10E-06 | 0.222259843 | 0.017118248 | 21 | 233 | Low |
| ENSG00000266261 | **AC005324.5* | 17 | p12 | 1.997500803 | 2.074386304 | 1.110626 | 0.003906 | 0.137653543 | 0.021623376 | 38 | 216 | Low |
| ENSG00000232006 | **AC005537.1* | 7 | p14.1 | 3.178933178 | 1.544100225 | 0.840045 | 0.009596 | 0.12111811 | 0.006700103 | 43 | 211 | Low |
| ENSG00000287390 | **AC006525.1* | 5 | q23.3 | 16.36523223 | 5.272728226 | 0.683703 | 1.95E-15 | 0.209472441 | 0.001400534 | 59 | 195 | Low |
| ENSG00000263680 | **AC007639.1* | 17 | q24.3 | 9.29121946 | 3.288047832 | 0.679285 | 1.80E-07 | 0.384492126 | 0.007404604 | 62 | 192 | Low |
| ENSG00000233491 | **AC008163.1* | 7 | q21.11 | 17.37186056 | 2.226730925 | 0.668034 | 7.18E-05 | 0.38861811 | 0.003855885 | 29 | 225 | Low |
| ENSG00000249738 | **AC008691.1* | 5 | q33.3 | 8.465965851 | 1.67084664 | 0.498783 | 0.000123 | 0.179484252 | 0.029095274 | 54 | 200 | Low |
| ENSG00000272554 | **AC012087.2* | 3 | p24.2 | 4.878606876 | 2.668394437 | 0.60675 | 1.38E-06 | 0.232271654 | 0.020142302 | 76 | 178 | Low |
| ENSG00000234327 | *AC012146.1* | 17 | p13.2 | 39.6396005 | 1.633537035 | 0.208848 | 4.21E-15 | 2.451295276 | 0.012386672 | 75 | 179 | Low |
| ENSG00000259772 | *AC012236.1* | 15 | q13.3 | 33.98982361 | 1.924942474 | 0.450755 | 2.71E-06 | 1.600641732 | 0.048553084 | 63 | 191 | Low |
| ENSG00000260597 | **AC012531.1* | 12 | q13.13 | 6.875888773 | 2.173293295 | 0.976407 | 0.00163 | 0.095334646 | 0.000868014 | 36 | 218 | Low |
| ENSG00000226468 | *AC018641.1* | 7 | p14.3 | 3.195022131 | 1.708278958 | 0.844026 | 0.00488 | 0.174102362 | 0.037277047 | 45 | 209 | Low |
| ENSG00000260648 | **AC020658.3* | 15 | q15.1 | 2.725770363 | 2.435474808 | 0.601733 | 5.48E-06 | 0.020594488 | 0.001285753 | 75 | 179 | Low |
| ENSG00000245482 | **AC046130.1* | 12 | p11.1 | 22.20332971 | 5.475415706 | 0.913469 | 1.50E-10 | 0.547885827 | 0.046880877 | 35 | 219 | Low |
| ENSG00000287045 | **AC072028.1* | 3 | q23 | 4.942621251 | 2.273388441 | 0.675333 | 6.28E-05 | 0.039948819 | 0.000724293 | 53 | 201 | Low |
| ENSG00000251550 | *AC091133.5* | 17 | q21.32 | 3.381788108 | 3.003326613 | 0.887791 | 3.90E-05 | 0.739232283 | 0.03439718 | 49 | 205 | Low |
| ENSG00000273679 | *AC100757.1* | 15 | q11.2 | 3.672869225 | 1.752754476 | 0.455193 | 1.76E-05 | 0.06292126 | 0.044188452 | 80 | 174 | Low |
| ENSG00000249803 | **AC112178.1* | 5 | q31.1 | 22.53325462 | 5.131559554 | 0.894529 | 5.84E-10 | 0.726129921 | 0.003675544 | 54 | 200 | Low |
| ENSG00000250049 | **AC114316.1* | 5 | q14.3 | 45.18664857 | 4.027599913 | 0.45536 | 5.35E-19 | 0.842519685 | 0.039460582 | 76 | 178 | Low |
| ENSG00000250612 | *AC114786.2* | 4 | q13.2 | 28.19627057 | 2.302534253 | 0.562772 | 4.75E-06 | 0.739787402 | 0.048622413 | 43 | 211 | Low |
| ENSG00000287862 | **AC114971.1* | 5 | q14.3 | 11.48612107 | 1.742650667 | 0.686664 | 0.00131 | 0.100047244 | 0.024510245 | 44 | 210 | Low |
| ENSG00000288029 | **AC115282.2* | 3 | p21.1 | 3.703837732 | 1.751680702 | 0.965251 | 0.006987 | 0.05376378 | 0.029944522 | 26 | 228 | Low |
| ENSG00000249413 | **AC116049.1* | 4 | q13.2 | 16.50971133 | 4.73128236 | 0.516183 | 1.49E-20 | 0.757106299 | 0.022071465 | 95 | 159 | Low |
| ENSG00000261069 | **AC124312.3* | 15 | q11.2 | 553.9042955 | 1.660704609 | 0.545334 | 0.000339 | 15.11248031 | 0.004924315 | 54 | 200 | Low |
| ENSG00000274840 | *AC132807.2* | 3 | p24.3 | 5.352009551 | 1.926642183 | 0.873963 | 0.002336 | 1.013850394 | 0.049868336 | 58 | 196 | Low |
| ENSG00000261020 | **AC132825.3* | 17 | p11.2 | 2.675100117 | 2.748543295 | 1.040913 | 0.000342 | 0.035129921 | 0.005294603 | 34 | 220 | Low |
| ENSG00000251468 | *AC135352.1* | 8 | p22 | 29.64203985 | -1.735527217 | 0.386954 | 1.33E-06 | 0.444862205 | 0.046829272 | 77 | 177 | Low |
| ENSG00000272108 | **AC244517.1* | 5 | q31.3 | 20.86602689 | 2.09571149 | 0.490067 | 2.40E-06 | 0.371779528 | 0.004552501 | 40 | 214 | Low |
| ENSG00000272942 | **AL022324.3* | 22 | q11.23 | 2.124498677 | 2.464225949 | 0.877444 | 0.000286 | 0.113598425 | 0.014177917 | 55 | 199 | Low |
| ENSG00000285407 | **AL033530.1* | 1 | p31.2 | 12.0934033 | 3.658003336 | 0.81116 | 5.24E-07 | 0.383224409 | 0.007979952 | 50 | 204 | Low |
| ENSG00000233358 | **AL035401.1* | 6 | p22.3 | 3.123245612 | 3.365448248 | 0.746972 | 6.74E-07 | 0.139370079 | 0.030572823 | 62 | 192 | Low |
| ENSG00000230973 | *AL049734.2* | X | q26.2 | 1.798914815 | 1.989447478 | 0.947029 | 0.002719 | 0.123814961 | 0.010267005 | 48 | 206 | Low |
| ENSG00000236389 | **AL121970.1* | 6 | q23.3 | 26.59203227 | 4.531311327 | 0.640755 | 2.05E-13 | 1.344885827 | 0.031194493 | 38 | 216 | Low |
| ENSG00000287923 | **AL138701.2* | 13 | q33.2 | 8.309542886 | 3.147976572 | 1.195492 | 0.000244 | 0.211125984 | 0.009270213 | 25 | 229 | Low |
| ENSG00000230234 | **AL162582.1* | 6 | q25.3 | 6.146123527 | 2.944805161 | 0.825275 | 2.31E-05 | 0.562228346 | 0.009375454 | 55 | 199 | Low |
| ENSG00000258793 | **AL355102.4* | 14 | q32.2 | 31.26921884 | 1.753375516 | 0.436168 | 9.13E-06 | 1.514003937 | 0.018490456 | 68 | 186 | Low |
| ENSG00000230533 | *AL356234.2* | 6 | q23.3 | 8.209364515 | 2.356882386 | 0.571608 | 4.16E-06 | 0.358267717 | 0.029834708 | 66 | 188 | Low |
| ENSG00000286060 | **AL365256.1* | X | q26.2 | 2.889701485 | 2.865090732 | 0.886443 | 6.62E-05 | 0.035779528 | 0.048681932 | 60 | 194 | Low |
| ENSG00000272954 | **AP000553.2* | 22 | q11.21 | 3.100770408 | 1.562609422 | 0.531241 | 0.000567 | 0.383122047 | 0.042795497 | 73 | 181 | Low |
| ENSG00000279793 | **AP000676.3* | 11 | q14.2 | 4.229858727 | 1.893646814 | 0.924757 | 0.003489 | 0.350858268 | 0.032987442 | 45 | 209 | Low |
| ENSG00000230789 | *ARHGAP26-IT1* | 5 | q31.3 | 2.756792256 | -1.699556295 | 0.341403 | 1.46E-07 | 0.23115748 | 0.006599674 | 82 | 172 | Low |
| ENSG00000170629 | *DPY19L2P2* | 7 | q22.1 | 68.67200209 | 1.898213849 | 0.339403 | 5.00E-09 | 0.731720472 | 0.048916749 | 50 | 204 | Low |
| ENSG00000183929 | *DUSP5P1* | 1 | q42.13 | 15.91539967 | 4.830746286 | 0.612654 | 5.11E-16 | 0.629625984 | 0.04017267 | 50 | 204 | Low |
| ENSG00000236081 | *ELFN1-AS1* | 7 | p22.3 | 4.580877113 | 1.964544171 | 0.886959 | 0.002169 | 0.333720472 | 0.019816311 | 43 | 211 | Low |
| ENSG00000180178 | *FAR2P1* | 2 | q21.1 | 64.21767021 | 6.841496454 | 0.93439 | 7.90E-14 | 2.235216535 | 0.049310212 | 37 | 217 | Low |
| ENSG00000123201 | *GUCY1B2* | 13 | q14.3 | 40.76386904 | 2.847429229 | 0.516597 | 9.24E-09 | 0.699444882 | 0.019537768 | 56 | 198 | Low |
| ENSG00000281344 | *HELLPAR* | 12 | q23.2 | 3621.294746 | 1.849646334 | 0.28429 | 2.60E-11 | 0.511255906 | 0.031211249 | 60 | 194 | Low |
| ENSG00000240990 | *HOXA11-AS* | 7 | p15.2 | 5.781497371 | 3.5516445 | 0.968356 | 1.11E-05 | 0.240944882 | 0.018497598 | 41 | 213 | Low |
| ENSG00000224853 | *LINC00393* | 13 | q22.1 | 1.662745538 | 1.804031615 | 0.951816 | 0.005495 | 0.588385827 | 0.006269375 | 7 | 247 | Low |
| ENSG00000250682 | *LINC00491* | 5 | q21.1 | 33.87952408 | 3.939446785 | 0.642241 | 1.42E-10 | 0.672712598 | 0.013558568 | 55 | 199 | Low |
| ENSG00000231532 | *LINC01249* | 2 | p25.2 | 2.295189244 | 2.043705628 | 0.974963 | 0.002555 | 0.068452756 | 0.006193358 | 46 | 208 | Low |
| ENSG00000225328 | *LINC01594* | 2 | q12.3 | 2.58282734 | 2.148480241 | 0.742165 | 0.000293 | 0.147625984 | 0.006503673 | 36 | 218 | Low |
| ENSG00000229243 | *LINC01981* | 3 | p24.1 | 2.821495416 | 1.973860046 | 1.00915 | 0.003778 | 0.039535433 | 0.012031446 | 45 | 209 | Low |
| ENSG00000229155 | *LINC02038* | 3 | q29 | 13.21957816 | 1.83684065 | 0.472434 | 1.37E-05 | 0.681692913 | 0.024112522 | 57 | 197 | Low |
| ENSG00000259485 | *LINC02253* | 15 | q26.2 | 5.184939084 | 3.66019058 | 1.20104 | 5.90E-05 | 0.216952756 | 0.006671765 | 27 | 227 | Low |
| ENSG00000203688 | *LINC02487* | 6 | q27 | 27.20853968 | 1.787136993 | 0.3856 | 6.38E-07 | 0.356708661 | 0.002192989 | 64 | 190 | Low |
| ENSG00000260265 | *LINC02562* | 4 | q13.3 | 8.049305571 | 2.421251264 | 0.922436 | 0.00048 | 0.411015748 | 0.003615403 | 59 | 195 | Low |
| ENSG00000254480 | *LINC02749* | 11 | p15.4 | 12.92832522 | 2.109178767 | 0.659894 | 0.000121 | 1.092972441 | 0.001358612 | 41 | 213 | Low |
| ENSG00000215424 | *MCM3AP-AS1* | 21 | q22.3 | 212.126593 | 2.707278605 | 0.238476 | 6.23E-29 | 2.736314961 | 0.047336661 | 87 | 167 | Low |
| ENSG00000251230 | *MIR3945HG* | 4 | q35.1 | 2.206149722 | -1.594140736 | 0.420996 | 2.99E-05 | 0.096610236 | 0.036649371 | 58 | 196 | Low |
| ENSG00000279864 | *NCOR1P4* | 21 | p11.2 | 3.195213839 | 2.274295587 | 1.344317 | 0.004019 | 0.327003937 | 0.043361417 | 20 | 234 | Low |
| ENSG00000181355 | *OFCC1* | 6 | p24.3 | 10.61780715 | 3.170195222 | 1.393006 | 0.000515 | 0.53980315 | 0.014513578 | 28 | 226 | Low |
| ENSG00000219926 | *OR7E104P* | 13 | q21.31 | 15.84125203 | 5.400037958 | 0.683691 | 5.22E-16 | 0.594314961 | 0.015435239 | 66 | 188 | Low |
| ENSG00000215418 | *PEX12P1* | 13 | q31.3 | 8.169170691 | 2.685056038 | 0.884196 | 0.00013 | 0.411338583 | 0.016380937 | 41 | 213 | Low |
| ENSG00000199197 | *RN7SKP72* | 18 | p11.32 | 2.995801625 | 1.748469259 | 0.900143 | 0.005453 | 0.661377953 | 0.037337226 | 43 | 211 | Low |
| ENSG00000240964 | *RN7SL751P* | 3 | p12.3 | 11.37436502 | 2.333085139 | 0.458994 | 6.73E-08 | 2.762114173 | 0.025712618 | 69 | 185 | Low |
| ENSG00000199347 | *RNU5E-1* | 1 | p36.22 | 4.560972489 | 2.897928196 | 0.711012 | 4.38E-06 | 7.898917323 | 0.001611243 | 66 | 188 | Low |
| ENSG00000199377 | *RNU5F-1* | 1 | p34.1 | 3.087900452 | 2.877498905 | 0.887375 | 6.37E-05 | 10.84712205 | 0.00113652 | 41 | 213 | Low |
| ENSG00000270722 | *RNVU1-31* | 1 | q21.1 | 127.5002018 | 2.251564757 | 0.351244 | 5.04E-11 | 98.18461024 | 0.015324055 | 64 | 190 | Low |
| ENSG00000243742 | *RPLP0P2* | 11 | q12.2 | 7.615509265 | 2.566890098 | 0.593561 | 1.86E-06 | 0.204531496 | 0.017632255 | 34 | 220 | Low |
| ENSG00000237506 | *RPSAP15* | X | q21.31 | 38.41287273 | 1.753788569 | 0.231526 | 2.14E-14 | 1.553224409 | 0.0077271 | 91 | 163 | Low |
| ENSG00000214244 | *SETP21* | 5 | q12.1 | 6.036118098 | 1.999486276 | 0.773278 | 0.000813 | 0.245913386 | 0.033280225 | 43 | 211 | Low |
| ENSG00000232727 | *YWHAEP1* | 7 | q11.21 | 5.806391201 | 3.391290974 | 0.711806 | 2.32E-07 | 0.287913386 | 0.04020207 | 47 | 207 | Low |

**Table S6**

**Table S6.** Association between expression of 229 genes and clinical characteristics in HCC patients stratified by (A) etiological factors and (B) gender.

(A)

| Gene_name |  | Level | Overall | Non-viral infection | Viral infection | p-value |
| --- | --- | --- | --- | --- | --- | --- |
| *HOXA11* | ENSG00000005073 (%) | High | 38 (15.0) | 3 ( 9.7) | 35 (15.7) | 0.541 |
|  |  | Low | 216 (85.0) | 28 ( 90.3) | 188 (84.3) |  |
| *TKTL1* | ENSG00000007350 (%) | High | 11 ( 4.3) | 0 ( 0.0) | 11 ( 4.9) | 0.428 |
|  |  | Low | 243 (95.7) | 31 (100.0) | 212 (95.1) |  |
| *PAX6* | ENSG00000007372 (%) | High | 36 (14.2) | 4 ( 12.9) | 32 (14.3) | 1 |
|  |  | Low | 218 (85.8) | 27 ( 87.1) | 191 (85.7) |  |
| *ZIC2* | ENSG00000043355 (%) | High | 78 (30.7) | 5 ( 16.1) | 73 (32.7) | 0.095 |
|  |  | Low | 176 (69.3) | 26 ( 83.9) | 150 (67.3) |  |
| *POLQ* | ENSG00000051341 (%) | High | 86 (33.9) | 6 ( 19.4) | 80 (35.9) | 0.106 |
|  |  | Low | 168 (66.1) | 25 ( 80.6) | 143 (64.1) |  |
| *CLDN18* | ENSG00000066405 (%) | High | 8 ( 3.1) | 0 ( 0.0) | 8 ( 3.6) | 0.601 |
|  |  | Low | 246 (96.9) | 31 (100.0) | 215 (96.4) |  |
| *PTGS2* | ENSG00000073756 (%) | High | 53 (20.9) | 5 ( 16.1) | 48 (21.5) | 0.648 |
|  |  | Low | 201 (79.1) | 26 ( 83.9) | 175 (78.5) |  |
| *CA12* | ENSG00000074410 (%) | High | 48 (18.9) | 6 ( 19.4) | 42 (18.8) | 1 |
|  |  | Low | 206 (81.1) | 25 ( 80.6) | 181 (81.2) |  |
| *CDH17* | ENSG00000079112 (%) | High | 15 ( 5.9) | 2 ( 6.5) | 13 ( 5.8) | 1 |
|  |  | Low | 239 (94.1) | 29 ( 93.5) | 210 (94.2) |  |
| *LYZ* | ENSG00000090382 (%) | High | 52 (20.5) | 4 ( 12.9) | 48 (21.5) | 0.38 |
|  |  | Low | 202 (79.5) | 27 ( 87.1) | 175 (78.5) |  |
| *SI* | ENSG00000090402 (%) | High | 10 ( 3.9) | 0 ( 0.0) | 10 ( 4.5) | 0.478 |
|  |  | Low | 244 (96.1) | 31 (100.0) | 213 (95.5) |  |
| *ORC6* | ENSG00000091651 (%) | High | 80 (31.5) | 7 ( 22.6) | 73 (32.7) | 0.35 |
|  |  | Low | 174 (68.5) | 24 ( 77.4) | 150 (67.3) |  |
| *Z83844.1* | ENSG00000100101 (%) | High | 16 ( 6.3) | 0 ( 0.0) | 16 ( 7.2) | 0.252 |
|  |  | Low | 238 (93.7) | 31 (100.0) | 207 (92.8) |  |
| *PVALB* | ENSG00000100362 (%) | High | 47 (18.5) | 6 ( 19.4) | 41 (18.4) | 1 |
|  |  | Low | 207 (81.5) | 25 ( 80.6) | 182 (81.6) |  |
| *CHGA* | ENSG00000100604 (%) | High | 22 ( 8.7) | 2 ( 6.5) | 20 ( 9.0) | 0.9 |
|  |  | Low | 232 (91.3) | 29 ( 93.5) | 203 (91.0) |  |
| *SMPD3* | ENSG00000103056 (%) | High | 68 (26.8) | 12 ( 38.7) | 56 (25.1) | 0.166 |
|  |  | Low | 186 (73.2) | 19 ( 61.3) | 167 (74.9) |  |
| *MEFV* | ENSG00000103313 (%) | High | 75 (29.5) | 8 ( 25.8) | 67 (30.0) | 0.784 |
|  |  | Low | 179 (70.5) | 23 ( 74.2) | 156 (70.0) |  |
| *RASL12* | ENSG00000103710 (%) | High | 82 (32.3) | 11 ( 35.5) | 71 (31.8) | 0.84 |
|  |  | Low | 172 (67.7) | 20 ( 64.5) | 152 (68.2) |  |
| *CCNE1* | ENSG00000105173 (%) | High | 42 (16.5) | 5 ( 16.1) | 37 (16.6) | 1 |
|  |  | Low | 212 (83.5) | 26 ( 83.9) | 186 (83.4) |  |
| *HAS1* | ENSG00000105509 (%) | High | 42 (16.5) | 3 ( 9.7) | 39 (17.5) | 0.401 |
|  |  | Low | 212 (83.5) | 28 ( 90.3) | 184 (82.5) |  |
| *FAM83E* | ENSG00000105523 (%) | High | 56 (22.0) | 5 ( 16.1) | 51 (22.9) | 0.537 |
|  |  | Low | 198 (78.0) | 26 ( 83.9) | 172 (77.1) |  |
| *TFPI2* | ENSG00000105825 (%) | High | 36 (14.2) | 0 ( 0.0) | 36 (16.1) | 0.032 |
|  |  | Low | 218 (85.8) | 31 (100.0) | 187 (83.9) |  |
| *ADAP1* | ENSG00000105963 (%) | High | 51 (20.1) | 4 ( 12.9) | 47 (21.1) | 0.409 |
|  |  | Low | 203 (79.9) | 27 ( 87.1) | 176 (78.9) |  |
| *SERPINE1* | ENSG00000106366 (%) | High | 85 (33.5) | 8 ( 25.8) | 77 (34.5) | 0.446 |
|  |  | Low | 169 (66.5) | 23 ( 74.2) | 146 (65.5) |  |
| *NPDC1* | ENSG00000107281 (%) | High | 85 (33.5) | 12 ( 38.7) | 73 (32.7) | 0.647 |
|  |  | Low | 169 (66.5) | 19 ( 61.3) | 150 (67.3) |  |
| *ASIC1* | ENSG00000110881 (%) | High | 68 (26.8) | 8 ( 25.8) | 60 (26.9) | 1 |
|  |  | Low | 186 (73.2) | 23 ( 74.2) | 163 (73.1) |  |
| *PCDHB2* | ENSG00000112852 (%) | High | 51 (20.1) | 3 ( 9.7) | 48 (21.5) | 0.192 |
|  |  | Low | 203 (79.9) | 28 ( 90.3) | 175 (78.5) |  |
| *PCDHB3* | ENSG00000113205 (%) | High | 42 (16.5) | 4 ( 12.9) | 38 (17.0) | 0.747 |
|  |  | Low | 212 (83.5) | 27 ( 87.1) | 185 (83.0) |  |
| *GABRG2* | ENSG00000113327 (%) | High | 31 (12.2) | 1 ( 3.2) | 30 (13.5) | 0.181 |
|  |  | Low | 223 (87.8) | 30 ( 96.8) | 193 (86.5) |  |
| *OTX1* | ENSG00000115507 (%) | High | 64 (25.2) | 6 ( 19.4) | 58 (26.0) | 0.563 |
|  |  | Low | 190 (74.8) | 25 ( 80.6) | 165 (74.0) |  |
| *APOA1* | ENSG00000118137 (%) | High | 104 (40.9) | 11 ( 35.5) | 93 (41.7) | 0.642 |
|  |  | Low | 150 (59.1) | 20 ( 64.5) | 130 (58.3) |  |
| *SPP1* | ENSG00000118785 (%) | High | 63 (24.8) | 7 ( 22.6) | 56 (25.1) | 0.933 |
|  |  | Low | 191 (75.2) | 24 ( 77.4) | 167 (74.9) |  |
| *CSF3R* | ENSG00000119535 (%) | High | 66 (26.0) | 7 ( 22.6) | 59 (26.5) | 0.808 |
|  |  | Low | 188 (74.0) | 24 ( 77.4) | 164 (73.5) |  |
| *PCDHB8* | ENSG00000120322 (%) | High | 42 (16.5) | 6 ( 19.4) | 36 (16.1) | 0.847 |
|  |  | Low | 212 (83.5) | 25 ( 80.6) | 187 (83.9) |  |
| *GUCY1B2* | ENSG00000123201 (%) | High | 56 (22.0) | 3 ( 9.7) | 53 (23.8) | 0.123 |
|  |  | Low | 198 (78.0) | 28 ( 90.3) | 170 (76.2) |  |
| *HJURP* | ENSG00000123485 (%) | High | 85 (33.5) | 6 ( 19.4) | 79 (35.4) | 0.116 |
|  |  | Low | 169 (66.5) | 25 ( 80.6) | 144 (64.6) |  |
| *NKX2-2* | ENSG00000125820 (%) | High | 17 ( 6.7) | 2 ( 6.5) | 15 ( 6.7) | 1 |
|  |  | Low | 237 (93.3) | 29 ( 93.5) | 208 (93.3) |  |
| *AHDC1* | ENSG00000126705 (%) | High | 99 (39.0) | 16 ( 51.6) | 83 (37.2) | 0.179 |
|  |  | Low | 155 (61.0) | 15 ( 48.4) | 140 (62.8) |  |
| *SIX1* | ENSG00000126778 (%) | High | 62 (24.4) | 9 ( 29.0) | 53 (23.8) | 0.677 |
|  |  | Low | 192 (75.6) | 22 ( 71.0) | 170 (76.2) |  |
| *DLGAP5* | ENSG00000126787 (%) | High | 78 (30.7) | 8 ( 25.8) | 70 (31.4) | 0.672 |
|  |  | Low | 176 (69.3) | 23 ( 74.2) | 153 (68.6) |  |
| *PZP* | ENSG00000126838 (%) | High | 26 (10.2) | 2 ( 6.5) | 24 (10.8) | 0.67 |
|  |  | Low | 228 (89.8) | 29 ( 93.5) | 199 (89.2) |  |
| *GAD1* | ENSG00000128683 (%) | High | 48 (18.9) | 3 ( 9.7) | 45 (20.2) | 0.248 |
|  |  | Low | 206 (81.1) | 28 ( 90.3) | 178 (79.8) |  |
| *PKDREJ* | ENSG00000130943 (%) | High | 66 (26.0) | 7 ( 22.6) | 59 (26.5) | 0.808 |
|  |  | Low | 188 (74.0) | 24 ( 77.4) | 164 (73.5) |  |
| *SPINK5* | ENSG00000133710 (%) | High | 64 (25.2) | 9 ( 29.0) | 55 (24.7) | 0.761 |
|  |  | Low | 190 (74.8) | 22 ( 71.0) | 168 (75.3) |  |
| *DTX1* | ENSG00000135144 (%) | High | 69 (27.2) | 7 ( 22.6) | 62 (27.8) | 0.691 |
|  |  | Low | 185 (72.8) | 24 ( 77.4) | 161 (72.2) |  |
| *IGF2BP3* | ENSG00000136231 (%) | High | 78 (30.7) | 3 ( 9.7) | 75 (33.6) | 0.012 |
|  |  | Low | 176 (69.3) | 28 ( 90.3) | 148 (66.4) |  |
| *ZIC5* | ENSG00000139800 (%) | High | 63 (24.8) | 5 ( 16.1) | 58 (26.0) | 0.331 |
|  |  | Low | 191 (75.2) | 26 ( 83.9) | 165 (74.0) |  |
| *GREB1L* | ENSG00000141449 (%) | High | 99 (39.0) | 9 ( 29.0) | 90 (40.4) | 0.31 |
|  |  | Low | 155 (61.0) | 22 ( 71.0) | 133 (59.6) |  |
| *NLRP12* | ENSG00000142405 (%) | High | 83 (32.7) | 8 ( 25.8) | 75 (33.6) | 0.505 |
|  |  | Low | 171 (67.3) | 23 ( 74.2) | 148 (66.4) |  |
| *CCNA2* | ENSG00000145386 (%) | High | 66 (26.0) | 4 ( 12.9) | 62 (27.8) | 0.12 |
|  |  | Low | 188 (74.0) | 27 ( 87.1) | 161 (72.2) |  |
| *FGD2* | ENSG00000146192 (%) | High | 88 (34.6) | 13 ( 41.9) | 75 (33.6) | 0.478 |
|  |  | Low | 166 (65.4) | 18 ( 58.1) | 148 (66.4) |  |
| *ASB15* | ENSG00000146809 (%) | High | 39 (15.4) | 9 ( 29.0) | 30 (13.5) | 0.047 |
|  |  | Low | 215 (84.6) | 22 ( 71.0) | 193 (86.5) |  |
| *NXF3* | ENSG00000147206 (%) | High | 33 (13.0) | 2 ( 6.5) | 31 (13.9) | 0.384 |
|  |  | Low | 221 (87.0) | 29 ( 93.5) | 192 (86.1) |  |
| *RGS20* | ENSG00000147509 (%) | High | 47 (18.5) | 2 ( 6.5) | 45 (20.2) | 0.11 |
|  |  | Low | 207 (81.5) | 29 ( 93.5) | 178 (79.8) |  |
| *ADIRF* | ENSG00000148671 (%) | High | 56 (22.0) | 11 ( 35.5) | 45 (20.2) | 0.09 |
|  |  | Low | 198 (78.0) | 20 ( 64.5) | 178 (79.8) |  |
| *CYP17A1* | ENSG00000148795 (%) | High | 44 (17.3) | 10 ( 32.3) | 34 (15.2) | 0.036 |
|  |  | Low | 210 (82.7) | 21 ( 67.7) | 189 (84.8) |  |
| *TLCD3B* | ENSG00000149926 (%) | High | 61 (24.0) | 7 ( 22.6) | 54 (24.2) | 1 |
|  |  | Low | 193 (76.0) | 24 ( 77.4) | 169 (75.8) |  |
| *HMGA2* | ENSG00000149948 (%) | High | 24 ( 9.4) | 1 ( 3.2) | 23 (10.3) | 0.349 |
|  |  | Low | 230 (90.6) | 30 ( 96.8) | 200 (89.7) |  |
| *SLC5A10* | ENSG00000154025 (%) | High | 33 (13.0) | 9 ( 29.0) | 24 (10.8) | 0.011 |
|  |  | Low | 221 (87.0) | 22 ( 71.0) | 199 (89.2) |  |
| *HOXB13* | ENSG00000159184 (%) | High | 18 ( 7.1) | 2 ( 6.5) | 16 ( 7.2) | 1 |
|  |  | Low | 236 (92.9) | 29 ( 93.5) | 207 (92.8) |  |
| *LY6E* | ENSG00000160932 (%) | High | 78 (30.7) | 13 ( 41.9) | 65 (29.1) | 0.216 |
|  |  | Low | 176 (69.3) | 18 ( 58.1) | 158 (70.9) |  |
| *FBXO27* | ENSG00000161243 (%) | High | 66 (26.0) | 8 ( 25.8) | 58 (26.0) | 1 |
|  |  | Low | 188 (74.0) | 23 ( 74.2) | 165 (74.0) |  |
| *ZSWIM5* | ENSG00000162415 (%) | High | 99 (39.0) | 12 ( 38.7) | 87 (39.0) | 1 |
|  |  | Low | 155 (61.0) | 19 ( 61.3) | 136 (61.0) |  |
| *XIRP2* | ENSG00000163092 (%) | High | 24 ( 9.4) | 1 ( 3.2) | 23 (10.3) | 0.349 |
|  |  | Low | 230 (90.6) | 30 ( 96.8) | 200 (89.7) |  |
| *CXCR1* | ENSG00000163464 (%) | High | 71 (28.0) | 8 ( 25.8) | 63 (28.3) | 0.944 |
|  |  | Low | 183 (72.0) | 23 ( 74.2) | 160 (71.7) |  |
| *EIF5A2* | ENSG00000163577 (%) | High | 82 (32.3) | 11 ( 35.5) | 71 (31.8) | 0.84 |
|  |  | Low | 172 (67.7) | 20 ( 64.5) | 152 (68.2) |  |
| *PPBP* | ENSG00000163736 (%) | High | 50 (19.7) | 7 ( 22.6) | 43 (19.3) | 0.848 |
|  |  | Low | 204 (80.3) | 24 ( 77.4) | 180 (80.7) |  |
| *TCF23* | ENSG00000163792 (%) | High | 74 (29.1) | 11 ( 35.5) | 63 (28.3) | 0.536 |
|  |  | Low | 180 (70.9) | 20 ( 64.5) | 160 (71.7) |  |
| *TMEM74* | ENSG00000164841 (%) | High | 73 (28.7) | 7 ( 22.6) | 66 (29.6) | 0.551 |
|  |  | Low | 181 (71.3) | 24 ( 77.4) | 157 (70.4) |  |
| *SLC16A9* | ENSG00000165449 (%) | High | 50 (19.7) | 4 ( 12.9) | 46 (20.6) | 0.44 |
|  |  | Low | 204 (80.3) | 27 ( 87.1) | 177 (79.4) |  |
| *C18orf54* | ENSG00000166845 (%) | High | 77 (30.3) | 6 ( 19.4) | 71 (31.8) | 0.227 |
|  |  | Low | 177 (69.7) | 25 ( 80.6) | 152 (68.2) |  |
| *KLK11* | ENSG00000167757 (%) | High | 23 ( 9.1) | 5 ( 16.1) | 18 ( 8.1) | 0.258 |
|  |  | Low | 231 (90.9) | 26 ( 83.9) | 205 (91.9) |  |
| *SIGLEC7* | ENSG00000168995 (%) | High | 84 (33.1) | 10 ( 32.3) | 74 (33.2) | 1 |
|  |  | Low | 170 (66.9) | 21 ( 67.7) | 149 (66.8) |  |
| *TMEM182* | ENSG00000170417 (%) | High | 60 (23.6) | 7 ( 22.6) | 53 (23.8) | 1 |
|  |  | Low | 194 (76.4) | 24 ( 77.4) | 170 (76.2) |  |
| *DPY19L2P2* | ENSG00000170629 (%) | High | 50 (19.7) | 7 ( 22.6) | 43 (19.3) | 0.848 |
|  |  | Low | 204 (80.3) | 24 ( 77.4) | 180 (80.7) |  |
| *TTLL6* | ENSG00000170703 (%) | High | 37 (14.6) | 2 ( 6.5) | 35 (15.7) | 0.273 |
|  |  | Low | 217 (85.4) | 29 ( 93.5) | 188 (84.3) |  |
| *CEACAM3* | ENSG00000170956 (%) | High | 74 (29.1) | 6 ( 19.4) | 68 (30.5) | 0.286 |
|  |  | Low | 180 (70.9) | 25 ( 80.6) | 155 (69.5) |  |
| *PKIA* | ENSG00000171033 (%) | High | 40 (15.7) | 4 ( 12.9) | 36 (16.1) | 0.841 |
|  |  | Low | 214 (84.3) | 27 ( 87.1) | 187 (83.9) |  |
| *FPR2* | ENSG00000171049 (%) | High | 70 (27.6) | 11 ( 35.5) | 59 (26.5) | 0.401 |
|  |  | Low | 184 (72.4) | 20 ( 64.5) | 164 (73.5) |  |
| *KRT20* | ENSG00000171431 (%) | High | 17 ( 6.7) | 0 ( 0.0) | 17 ( 7.6) | 0.227 |
|  |  | Low | 237 (93.3) | 31 (100.0) | 206 (92.4) |  |
| *ABCG4* | ENSG00000172350 (%) | High | 77 (30.3) | 6 ( 19.4) | 71 (31.8) | 0.227 |
|  |  | Low | 177 (69.7) | 25 ( 80.6) | 152 (68.2) |  |
| *GXYLT2* | ENSG00000172986 (%) | High | 60 (23.6) | 4 ( 12.9) | 56 (25.1) | 0.203 |
|  |  | Low | 194 (76.4) | 27 ( 87.1) | 167 (74.9) |  |
| *OLR1* | ENSG00000173391 (%) | High | 50 (19.7) | 6 ( 19.4) | 44 (19.7) | 1 |
|  |  | Low | 204 (80.3) | 25 ( 80.6) | 179 (80.3) |  |
| *MCMDC2* | ENSG00000178460 (%) | High | 88 (34.6) | 11 ( 35.5) | 77 (34.5) | 1 |
|  |  | Low | 166 (65.4) | 20 ( 64.5) | 146 (65.5) |  |
| *FOXS1* | ENSG00000179772 (%) | High | 96 (37.8) | 11 ( 35.5) | 85 (38.1) | 0.932 |
|  |  | Low | 158 (62.2) | 20 ( 64.5) | 138 (61.9) |  |
| *FAR2P1* | ENSG00000180178 (%) | High | 37 (14.6) | 3 ( 9.7) | 34 (15.2) | 0.581 |
|  |  | Low | 217 (85.4) | 28 ( 90.3) | 189 (84.8) |  |
| *ALX1* | ENSG00000180318 (%) | High | 25 ( 9.8) | 1 ( 3.2) | 24 (10.8) | 0.318 |
|  |  | Low | 229 (90.2) | 30 ( 96.8) | 199 (89.2) |  |
| *MAPK15* | ENSG00000181085 (%) | High | 33 (13.0) | 4 ( 12.9) | 29 (13.0) | 1 |
|  |  | Low | 221 (87.0) | 27 ( 87.1) | 194 (87.0) |  |
| *OFCC1* | ENSG00000181355 (%) | High | 28 (11.0) | 2 ( 6.5) | 26 (11.7) | 0.574 |
|  |  | Low | 226 (89.0) | 29 ( 93.5) | 197 (88.3) |  |
| *FDCSP* | ENSG00000181617 (%) | High | 12 ( 4.7) | 2 ( 6.5) | 10 ( 4.5) | 0.974 |
|  |  | Low | 242 (95.3) | 29 ( 93.5) | 213 (95.5) |  |
| *PLAG1* | ENSG00000181690 (%) | High | 79 (31.1) | 10 ( 32.3) | 69 (30.9) | 1 |
|  |  | Low | 175 (68.9) | 21 ( 67.7) | 154 (69.1) |  |
| *MACIR* | ENSG00000181751 (%) | High | 74 (29.1) | 11 ( 35.5) | 63 (28.3) | 0.536 |
|  |  | Low | 180 (70.9) | 20 ( 64.5) | 160 (71.7) |  |
| *C2CD4C* | ENSG00000183186 (%) | High | 80 (31.5) | 12 ( 38.7) | 68 (30.5) | 0.474 |
|  |  | Low | 174 (68.5) | 19 ( 61.3) | 155 (69.5) |  |
| *DUSP5P1* | ENSG00000183929 (%) | High | 50 (19.7) | 8 ( 25.8) | 42 (18.8) | 0.5 |
|  |  | Low | 204 (80.3) | 23 ( 74.2) | 181 (81.2) |  |
| *PKP3* | ENSG00000184363 (%) | High | 25 ( 9.8) | 3 ( 9.7) | 22 ( 9.9) | 1 |
|  |  | Low | 229 (90.2) | 28 ( 90.3) | 201 (90.1) |  |
| *MUC6* | ENSG00000184956 (%) | High | 14 ( 5.5) | 1 ( 3.2) | 13 ( 5.8) | 0.861 |
|  |  | Low | 240 (94.5) | 30 ( 96.8) | 210 (94.2) |  |
| *RFX6* | ENSG00000185002 (%) | High | 19 ( 7.5) | 1 ( 3.2) | 18 ( 8.1) | 0.551 |
|  |  | Low | 235 (92.5) | 30 ( 96.8) | 205 (91.9) |  |
| *ANKS1B* | ENSG00000185046 (%) | High | 62 (24.4) | 2 ( 6.5) | 60 (26.9) | 0.024 |
|  |  | Low | 192 (75.6) | 29 ( 93.5) | 163 (73.1) |  |
| *FOXI2* | ENSG00000186766 (%) | High | 74 (29.1) | 10 ( 32.3) | 64 (28.7) | 0.843 |
|  |  | Low | 180 (70.9) | 21 ( 67.7) | 159 (71.3) |  |
| *POTEG* | ENSG00000187537 (%) | High | 26 (10.2) | 1 ( 3.2) | 25 (11.2) | 0.29 |
|  |  | Low | 228 (89.8) | 30 ( 96.8) | 198 (88.8) |  |
| *DMBT1* | ENSG00000187908 (%) | High | 18 ( 7.1) | 1 ( 3.2) | 17 ( 7.6) | 0.603 |
|  |  | Low | 236 (92.9) | 30 ( 96.8) | 206 (92.4) |  |
| *IL1RAPL2* | ENSG00000189108 (%) | High | 66 (26.0) | 11 ( 35.5) | 55 (24.7) | 0.285 |
|  |  | Low | 188 (74.0) | 20 ( 64.5) | 168 (75.3) |  |
| *ALG1L* | ENSG00000189366 (%) | High | 84 (33.1) | 9 ( 29.0) | 75 (33.6) | 0.759 |
|  |  | Low | 170 (66.9) | 22 ( 71.0) | 148 (66.4) |  |
| *MYBPC1* | ENSG00000196091 (%) | High | 7 ( 2.8) | 1 ( 3.2) | 6 ( 2.7) | 1 |
|  |  | Low | 247 (97.2) | 30 ( 96.8) | 217 (97.3) |  |
| *H2AC11* | ENSG00000196787 (%) | High | 87 (34.3) | 12 ( 38.7) | 75 (33.6) | 0.722 |
|  |  | Low | 167 (65.7) | 19 ( 61.3) | 148 (66.4) |  |
| *ZNF679* | ENSG00000197123 (%) | High | 31 (12.2) | 1 ( 3.2) | 30 (13.5) | 0.181 |
|  |  | Low | 223 (87.8) | 30 ( 96.8) | 193 (86.5) |  |
| *CYP2B6* | ENSG00000197408 (%) | High | 82 (32.3) | 8 ( 25.8) | 74 (33.2) | 0.536 |
|  |  | Low | 172 (67.7) | 23 ( 74.2) | 149 (66.8) |  |
| *POTEH* | ENSG00000198062 (%) | High | 25 ( 9.8) | 1 ( 3.2) | 24 (10.8) | 0.318 |
|  |  | Low | 229 (90.2) | 30 ( 96.8) | 199 (89.2) |  |
| *STK39* | ENSG00000198648 (%) | High | 84 (33.1) | 10 ( 32.3) | 74 (33.2) | 1 |
|  |  | Low | 170 (66.9) | 21 ( 67.7) | 149 (66.8) |  |
| *EPS8L3* | ENSG00000198758 (%) | High | 68 (26.8) | 10 ( 32.3) | 58 (26.0) | 0.603 |
|  |  | Low | 186 (73.2) | 21 ( 67.7) | 165 (74.0) |  |
| *RN7SKP72* | ENSG00000199197 (%) | High | 43 (16.9) | 2 ( 6.5) | 41 (18.4) | 0.16 |
|  |  | Low | 211 (83.1) | 29 ( 93.5) | 182 (81.6) |  |
| *RNU5E-1* | ENSG00000199347 (%) | High | 66 (26.0) | 6 ( 19.4) | 60 (26.9) | 0.497 |
|  |  | Low | 188 (74.0) | 25 ( 80.6) | 163 (73.1) |  |
| *RNU5F-1* | ENSG00000199377 (%) | High | 41 (16.1) | 3 ( 9.7) | 38 (17.0) | 0.433 |
|  |  | Low | 213 (83.9) | 28 ( 90.3) | 185 (83.0) |  |
| *LINC02487* | ENSG00000203688 (%) | High | 64 (25.2) | 6 ( 19.4) | 58 (26.0) | 0.563 |
|  |  | Low | 190 (74.8) | 25 ( 80.6) | 165 (74.0) |  |
| *RIPPLY2* | ENSG00000203877 (%) | High | 28 (11.0) | 2 ( 6.5) | 26 (11.7) | 0.574 |
|  |  | Low | 226 (89.0) | 29 ( 93.5) | 197 (88.3) |  |
| *TRIM40* | ENSG00000204614 (%) | High | 38 (15.0) | 5 ( 16.1) | 33 (14.8) | 1 |
|  |  | Low | 216 (85.0) | 26 ( 83.9) | 190 (85.2) |  |
| *ASPDH* | ENSG00000204653 (%) | High | 93 (36.6) | 13 ( 41.9) | 80 (35.9) | 0.647 |
|  |  | Low | 161 (63.4) | 18 ( 58.1) | 143 (64.1) |  |
| *TMEM88B* | ENSG00000205116 (%) | High | 44 (17.3) | 6 ( 19.4) | 38 (17.0) | 0.948 |
|  |  | Low | 210 (82.7) | 25 ( 80.6) | 185 (83.0) |  |
| *AC108134.1* | ENSG00000205890 (%) | High | 36 (14.2) | 6 ( 19.4) | 30 (13.5) | 0.543 |
|  |  | Low | 218 (85.8) | 25 ( 80.6) | 193 (86.5) |  |
| *DEFA1* | ENSG00000206047 (%) | High | 46 (18.1) | 3 ( 9.7) | 43 (19.3) | 0.293 |
|  |  | Low | 208 (81.9) | 28 ( 90.3) | 180 (80.7) |  |
| *FOXL2NB* | ENSG00000206262 (%) | High | 46 (18.1) | 5 ( 16.1) | 41 (18.4) | 0.955 |
|  |  | Low | 208 (81.9) | 26 ( 83.9) | 182 (81.6) |  |
| *TRIM71* | ENSG00000206557 (%) | High | 58 (22.8) | 5 ( 16.1) | 53 (23.8) | 0.471 |
|  |  | Low | 196 (77.2) | 26 ( 83.9) | 170 (76.2) |  |
| *SETP21* | ENSG00000214244 (%) | High | 43 (16.9) | 5 ( 16.1) | 38 (17.0) | 1 |
|  |  | Low | 211 (83.1) | 26 ( 83.9) | 185 (83.0) |  |
| *PEX12P1* | ENSG00000215418 (%) | High | 41 (16.1) | 5 ( 16.1) | 36 (16.1) | 1 |
|  |  | Low | 213 (83.9) | 26 ( 83.9) | 187 (83.9) |  |
| *MCM3AP-AS1* | ENSG00000215424 (%) | High | 87 (34.3) | 9 ( 29.0) | 78 (35.0) | 0.652 |
|  |  | Low | 167 (65.7) | 22 ( 71.0) | 145 (65.0) |  |
| *OR7E104P* | ENSG00000219926 (%) | High | 66 (26.0) | 6 ( 19.4) | 60 (26.9) | 0.497 |
|  |  | Low | 188 (74.0) | 25 ( 80.6) | 163 (73.1) |  |
| *EBF2* | ENSG00000221818 (%) | High | 88 (34.6) | 11 ( 35.5) | 77 (34.5) | 1 |
|  |  | Low | 166 (65.4) | 20 ( 64.5) | 146 (65.5) |  |
| *MYBPHL* | ENSG00000221986 (%) | High | 41 (16.1) | 2 ( 6.5) | 39 (17.5) | 0.192 |
|  |  | Low | 213 (83.9) | 29 ( 93.5) | 184 (82.5) |  |
| *ATP5F1CP1* | ENSG00000224004 (%) | High | 34 (13.4) | 3 ( 9.7) | 31 (13.9) | 0.715 |
|  |  | Low | 220 (86.6) | 28 ( 90.3) | 192 (86.1) |  |
| *LINC00393* | ENSG00000224853 (%) | High | 7 ( 2.8) | 1 ( 3.2) | 6 ( 2.7) | 1 |
|  |  | Low | 247 (97.2) | 30 ( 96.8) | 217 (97.3) |  |
| *LINC01594* | ENSG00000225328 (%) | High | 36 (14.2) | 5 ( 16.1) | 31 (13.9) | 0.953 |
|  |  | Low | 218 (85.8) | 26 ( 83.9) | 192 (86.1) |  |
| *AL592114.2* | ENSG00000225522 (%) | High | 43 (16.9) | 5 ( 16.1) | 38 (17.0) | 1 |
|  |  | Low | 211 (83.1) | 26 ( 83.9) | 185 (83.0) |  |
| *AC018641.1* | ENSG00000226468 (%) | High | 45 (17.7) | 3 ( 9.7) | 42 (18.8) | 0.317 |
|  |  | Low | 209 (82.3) | 28 ( 90.3) | 181 (81.2) |  |
| *RPL39P40* | ENSG00000226580 (%) | High | 72 (28.3) | 8 ( 25.8) | 64 (28.7) | 0.903 |
|  |  | Low | 182 (71.7) | 23 ( 74.2) | 159 (71.3) |  |
| *AC092155.1* | ENSG00000226622 (%) | High | 56 (22.0) | 8 ( 25.8) | 48 (21.5) | 0.758 |
|  |  | Low | 198 (78.0) | 23 ( 74.2) | 175 (78.5) |  |
| *CYP4F29P* | ENSG00000228314 (%) | High | 54 (21.3) | 6 ( 19.4) | 48 (21.5) | 0.966 |
|  |  | Low | 200 (78.7) | 25 ( 80.6) | 175 (78.5) |  |
| *LINC02038* | ENSG00000229155 (%) | High | 58 (22.8) | 6 ( 19.4) | 52 (23.3) | 0.792 |
|  |  | Low | 196 (77.2) | 25 ( 80.6) | 171 (76.7) |  |
| *LINC01981* | ENSG00000229243 (%) | High | 45 (17.7) | 2 ( 6.5) | 43 (19.3) | 0.133 |
|  |  | Low | 209 (82.3) | 29 ( 93.5) | 180 (80.7) |  |
| *AL162582.1* | ENSG00000230234 (%) | High | 55 (21.7) | 11 ( 35.5) | 44 (19.7) | 0.078 |
|  |  | Low | 199 (78.3) | 20 ( 64.5) | 179 (80.3) |  |
| *AL356234.2* | ENSG00000230533 (%) | High | 67 (26.4) | 6 ( 19.4) | 61 (27.4) | 0.466 |
|  |  | Low | 187 (73.6) | 25 ( 80.6) | 162 (72.6) |  |
| *ARHGAP26-IT1* | ENSG00000230789 (%) | High | 83 (32.7) | 7 ( 22.6) | 76 (34.1) | 0.282 |
|  |  | Low | 171 (67.3) | 24 ( 77.4) | 147 (65.9) |  |
| *DPP4-DT* | ENSG00000230918 (%) | High | 64 (25.2) | 6 ( 19.4) | 58 (26.0) | 0.563 |
|  |  | Low | 190 (74.8) | 25 ( 80.6) | 165 (74.0) |  |
| *AL049734.2* | ENSG00000230973 (%) | High | 48 (18.9) | 3 ( 9.7) | 45 (20.2) | 0.248 |
|  |  | Low | 206 (81.1) | 28 ( 90.3) | 178 (79.8) |  |
| *LINC01249* | ENSG00000231532 (%) | High | 46 (18.1) | 3 ( 9.7) | 43 (19.3) | 0.293 |
|  |  | Low | 208 (81.9) | 28 ( 90.3) | 180 (80.7) |  |
| *AC005537.1* | ENSG00000232006 (%) | High | 43 (16.9) | 3 ( 9.7) | 40 (17.9) | 0.372 |
|  |  | Low | 211 (83.1) | 28 ( 90.3) | 183 (82.1) |  |
| *YWHAEP1* | ENSG00000232727 (%) | High | 47 (18.5) | 3 ( 9.7) | 44 (19.7) | 0.27 |
|  |  | Low | 207 (81.5) | 28 ( 90.3) | 179 (80.3) |  |
| *AL035401.1* | ENSG00000233358 (%) | High | 62 (24.4) | 6 ( 19.4) | 56 (25.1) | 0.634 |
|  |  | Low | 192 (75.6) | 25 ( 80.6) | 167 (74.9) |  |
| *FHAD1-AS1* | ENSG00000233485 (%) | High | 27 (10.6) | 4 ( 12.9) | 23 (10.3) | 0.899 |
|  |  | Low | 227 (89.4) | 27 ( 87.1) | 200 (89.7) |  |
| *AC008163.1* | ENSG00000233491 (%) | High | 29 (11.4) | 3 ( 9.7) | 26 (11.7) | 0.981 |
|  |  | Low | 225 (88.6) | 28 ( 90.3) | 197 (88.3) |  |
| *AC012146.1* | ENSG00000234327 (%) | High | 75 (29.5) | 12 ( 38.7) | 63 (28.3) | 0.324 |
|  |  | Low | 179 (70.5) | 19 ( 61.3) | 160 (71.7) |  |
| *C12orf75* | ENSG00000235162 (%) | High | 61 (24.0) | 4 ( 12.9) | 57 (25.6) | 0.186 |
|  |  | Low | 193 (76.0) | 27 ( 87.1) | 166 (74.4) |  |
| *AC023347.1* | ENSG00000235774 (%) | High | 57 (22.4) | 5 ( 16.1) | 52 (23.3) | 0.503 |
|  |  | Low | 197 (77.6) | 26 ( 83.9) | 171 (76.7) |  |
| *ELFN1-AS1* | ENSG00000236081 (%) | High | 43 (16.9) | 4 ( 12.9) | 39 (17.5) | 0.702 |
|  |  | Low | 211 (83.1) | 27 ( 87.1) | 184 (82.5) |  |
| *AL121970.1* | ENSG00000236389 (%) | High | 38 (15.0) | 4 ( 12.9) | 34 (15.2) | 0.941 |
|  |  | Low | 216 (85.0) | 27 ( 87.1) | 189 (84.8) |  |
| *RPSAP15* | ENSG00000237506 (%) | High | 91 (35.8) | 9 ( 29.0) | 82 (36.8) | 0.521 |
|  |  | Low | 163 (64.2) | 22 ( 71.0) | 141 (63.2) |  |
| *RN7SL751P* | ENSG00000240964 (%) | High | 69 (27.2) | 5 ( 16.1) | 64 (28.7) | 0.208 |
|  |  | Low | 185 (72.8) | 26 ( 83.9) | 159 (71.3) |  |
| *HOXA11-AS* | ENSG00000240990 (%) | High | 41 (16.1) | 3 ( 9.7) | 38 (17.0) | 0.433 |
|  |  | Low | 213 (83.9) | 28 ( 90.3) | 185 (83.0) |  |
| *ADAMTS9-AS1* | ENSG00000241158 (%) | High | 55 (21.7) | 5 ( 16.1) | 50 (22.4) | 0.573 |
|  |  | Low | 199 (78.3) | 26 ( 83.9) | 173 (77.6) |  |
| *RPLP0P2* | ENSG00000243742 (%) | High | 34 (13.4) | 3 ( 9.7) | 31 (13.9) | 0.715 |
|  |  | Low | 220 (86.6) | 28 ( 90.3) | 192 (86.1) |  |
| *GMCL2* | ENSG00000244234 (%) | High | 21 ( 8.3) | 2 ( 6.5) | 19 ( 8.5) | 0.965 |
|  |  | Low | 233 (91.7) | 29 ( 93.5) | 204 (91.5) |  |
| *IFITM10* | ENSG00000244242 (%) | High | 55 (21.7) | 6 ( 19.4) | 49 (22.0) | 0.921 |
|  |  | Low | 199 (78.3) | 25 ( 80.6) | 174 (78.0) |  |
| *AC046130.1* | ENSG00000245482 (%) | High | 35 (13.8) | 5 ( 16.1) | 30 (13.5) | 0.899 |
|  |  | Low | 219 (86.2) | 26 ( 83.9) | 193 (86.5) |  |
| *AC120114.1* | ENSG00000247735 (%) | High | 86 (33.9) | 13 ( 41.9) | 73 (32.7) | 0.417 |
|  |  | Low | 168 (66.1) | 18 ( 58.1) | 150 (67.3) |  |
| *AC021134.1* | ENSG00000248431 (%) | High | 31 (12.2) | 3 ( 9.7) | 28 (12.6) | 0.868 |
|  |  | Low | 223 (87.8) | 28 ( 90.3) | 195 (87.4) |  |
| *AC097534.1* | ENSG00000248774 (%) | High | 79 (31.1) | 9 ( 29.0) | 70 (31.4) | 0.953 |
|  |  | Low | 175 (68.9) | 22 ( 71.0) | 153 (68.6) |  |
| *UGT2A3P7* | ENSG00000248886 (%) | High | 79 (31.1) | 8 ( 25.8) | 71 (31.8) | 0.636 |
|  |  | Low | 175 (68.9) | 23 ( 74.2) | 152 (68.2) |  |
| *AC116049.1* | ENSG00000249413 (%) | High | 95 (37.4) | 7 ( 22.6) | 88 (39.5) | 0.105 |
|  |  | Low | 159 (62.6) | 24 ( 77.4) | 135 (60.5) |  |
| *AC008691.1* | ENSG00000249738 (%) | High | 54 (21.3) | 5 ( 16.1) | 49 (22.0) | 0.609 |
|  |  | Low | 200 (78.7) | 26 ( 83.9) | 174 (78.0) |  |
| *AC112178.1* | ENSG00000249803 (%) | High | 54 (21.3) | 3 ( 9.7) | 51 (22.9) | 0.148 |
|  |  | Low | 200 (78.7) | 28 ( 90.3) | 172 (77.1) |  |
| *AC114316.1* | ENSG00000250049 (%) | High | 77 (30.3) | 7 ( 22.6) | 70 (31.4) | 0.429 |
|  |  | Low | 177 (69.7) | 24 ( 77.4) | 153 (68.6) |  |
| *LINC02200* | ENSG00000250358 (%) | High | 38 (15.0) | 4 ( 12.9) | 34 (15.2) | 0.941 |
|  |  | Low | 216 (85.0) | 27 ( 87.1) | 189 (84.8) |  |
| *AACSP1* | ENSG00000250420 (%) | High | 21 ( 8.3) | 0 ( 0.0) | 21 ( 9.4) | 0.151 |
|  |  | Low | 233 (91.7) | 31 (100.0) | 202 (90.6) |  |
| *AC114786.2* | ENSG00000250612 (%) | High | 43 (16.9) | 4 ( 12.9) | 39 (17.5) | 0.702 |
|  |  | Low | 211 (83.1) | 27 ( 87.1) | 184 (82.5) |  |
| *LINC00491* | ENSG00000250682 (%) | High | 55 (21.7) | 3 ( 9.7) | 52 (23.3) | 0.135 |
|  |  | Low | 199 (78.3) | 28 ( 90.3) | 171 (76.7) |  |
| *MIR3945HG* | ENSG00000251230 (%) | High | 58 (22.8) | 8 ( 25.8) | 50 (22.4) | 0.847 |
|  |  | Low | 196 (77.2) | 23 ( 74.2) | 173 (77.6) |  |
| *AC135352.1* | ENSG00000251468 (%) | High | 77 (30.3) | 12 ( 38.7) | 65 (29.1) | 0.381 |
|  |  | Low | 177 (69.7) | 19 ( 61.3) | 158 (70.9) |  |
| *AC091133.5* | ENSG00000251550 (%) | High | 49 (19.3) | 2 ( 6.5) | 47 (21.1) | 0.091 |
|  |  | Low | 205 (80.7) | 29 ( 93.5) | 176 (78.9) |  |
| *HOXA10* | ENSG00000253293 (%) | High | 64 (25.2) | 6 ( 19.4) | 58 (26.0) | 0.563 |
|  |  | Low | 190 (74.8) | 25 ( 80.6) | 165 (74.0) |  |
| *AC105118.1* | ENSG00000253931 (%) | High | 15 ( 5.9) | 2 ( 6.5) | 13 ( 5.8) | 1 |
|  |  | Low | 239 (94.1) | 29 ( 93.5) | 210 (94.2) |  |
| *LINC02749* | ENSG00000254480 (%) | High | 41 (16.1) | 6 ( 19.4) | 35 (15.7) | 0.796 |
|  |  | Low | 213 (83.9) | 25 ( 80.6) | 188 (84.3) |  |
| *AC137894.1* | ENSG00000254739 (%) | High | 75 (29.5) | 11 ( 35.5) | 64 (28.7) | 0.572 |
|  |  | Low | 179 (70.5) | 20 ( 64.5) | 159 (71.3) |  |
| *CCDC84-DT* | ENSG00000255121 (%) | High | 76 (29.9) | 5 ( 16.1) | 71 (31.8) | 0.114 |
|  |  | Low | 178 (70.1) | 26 ( 83.9) | 152 (68.2) |  |
| *GPR142* | ENSG00000257008 (%) | High | 46 (18.1) | 4 ( 12.9) | 42 (18.8) | 0.579 |
|  |  | Low | 208 (81.9) | 27 ( 87.1) | 181 (81.2) |  |
| *MGAM2* | ENSG00000257743 (%) | High | 38 (15.0) | 4 ( 12.9) | 34 (15.2) | 0.941 |
|  |  | Low | 216 (85.0) | 27 ( 87.1) | 189 (84.8) |  |
| *AL355102.4* | ENSG00000258793 (%) | High | 67 (26.4) | 4 ( 12.9) | 63 (28.3) | 0.11 |
|  |  | Low | 187 (73.6) | 27 ( 87.1) | 160 (71.7) |  |
| *LINC02883* | ENSG00000259416 (%) | High | 80 (31.5) | 9 ( 29.0) | 71 (31.8) | 0.913 |
|  |  | Low | 174 (68.5) | 22 ( 71.0) | 152 (68.2) |  |
| *LINC02253* | ENSG00000259485 (%) | High | 27 (10.6) | 3 ( 9.7) | 24 (10.8) | 1 |
|  |  | Low | 227 (89.4) | 28 ( 90.3) | 199 (89.2) |  |
| *AC012236.1* | ENSG00000259772 (%) | High | 63 (24.8) | 10 ( 32.3) | 53 (23.8) | 0.422 |
|  |  | Low | 191 (75.2) | 21 ( 67.7) | 170 (76.2) |  |
| *LINC02562* | ENSG00000260265 (%) | High | 59 (23.2) | 4 ( 12.9) | 55 (24.7) | 0.22 |
|  |  | Low | 195 (76.8) | 27 ( 87.1) | 168 (75.3) |  |
| *AC012531.1* | ENSG00000260597 (%) | High | 36 (14.2) | 4 ( 12.9) | 32 (14.3) | 1 |
|  |  | Low | 218 (85.8) | 27 ( 87.1) | 191 (85.7) |  |
| *AC020658.3* | ENSG00000260648 (%) | High | 75 (29.5) | 9 ( 29.0) | 66 (29.6) | 1 |
|  |  | Low | 179 (70.5) | 22 ( 71.0) | 157 (70.4) |  |
| *AC106820.5* | ENSG00000260874 (%) | High | 92 (36.2) | 8 ( 25.8) | 84 (37.7) | 0.277 |
|  |  | Low | 162 (63.8) | 23 ( 74.2) | 139 (62.3) |  |
| *DOCK9-DT* | ENSG00000260992 (%) | High | 90 (35.4) | 13 ( 41.9) | 77 (34.5) | 0.544 |
|  |  | Low | 164 (64.6) | 18 ( 58.1) | 146 (65.5) |  |
| *AC132825.3* | ENSG00000261020 (%) | High | 34 (13.4) | 2 ( 6.5) | 32 (14.3) | 0.353 |
|  |  | Low | 220 (86.6) | 29 ( 93.5) | 191 (85.7) |  |
| *AC124312.3* | ENSG00000261069 (%) | High | 54 (21.3) | 4 ( 12.9) | 50 (22.4) | 0.327 |
|  |  | Low | 200 (78.7) | 27 ( 87.1) | 173 (77.6) |  |
| *AC133919.2* | ENSG00000261172 (%) | High | 69 (27.2) | 11 ( 35.5) | 58 (26.0) | 0.37 |
|  |  | Low | 185 (72.8) | 20 ( 64.5) | 165 (74.0) |  |
| *AC022167.4* | ENSG00000261481 (%) | High | 54 (21.3) | 11 ( 35.5) | 43 (19.3) | 0.067 |
|  |  | Low | 200 (78.7) | 20 ( 64.5) | 180 (80.7) |  |
| *FAM72C* | ENSG00000263513 (%) | High | 62 (24.4) | 5 ( 16.1) | 57 (25.6) | 0.356 |
|  |  | Low | 192 (75.6) | 26 ( 83.9) | 166 (74.4) |  |
| *AC007639.1* | ENSG00000263680 (%) | High | 62 (24.4) | 7 ( 22.6) | 55 (24.7) | 0.976 |
|  |  | Low | 192 (75.6) | 24 ( 77.4) | 168 (75.3) |  |
| *AC005324.5* | ENSG00000266261 (%) | High | 38 (15.0) | 5 ( 16.1) | 33 (14.8) | 1 |
|  |  | Low | 216 (85.0) | 26 ( 83.9) | 190 (85.2) |  |
| *RNVU1-31* | ENSG00000270722 (%) | High | 64 (25.2) | 8 ( 25.8) | 56 (25.1) | 1 |
|  |  | Low | 190 (74.8) | 23 ( 74.2) | 167 (74.9) |  |
| *AC244517.1* | ENSG00000272108 (%) | High | 40 (15.7) | 5 ( 16.1) | 35 (15.7) | 1 |
|  |  | Low | 214 (84.3) | 26 ( 83.9) | 188 (84.3) |  |
| *AC012087.2* | ENSG00000272554 (%) | High | 76 (29.9) | 7 ( 22.6) | 69 (30.9) | 0.457 |
|  |  | Low | 178 (70.1) | 24 ( 77.4) | 154 (69.1) |  |
| *AL022324.3* | ENSG00000272942 (%) | High | 55 (21.7) | 2 ( 6.5) | 53 (23.8) | 0.05 |
|  |  | Low | 199 (78.3) | 29 ( 93.5) | 170 (76.2) |  |
| *AP000553.2* | ENSG00000272954 (%) | High | 73 (28.7) | 6 ( 19.4) | 67 (30.0) | 0.307 |
|  |  | Low | 181 (71.3) | 25 ( 80.6) | 156 (70.0) |  |
| *AC100757.1* | ENSG00000273679 (%) | High | 81 (31.9) | 8 ( 25.8) | 73 (32.7) | 0.569 |
|  |  | Low | 173 (68.1) | 23 ( 74.2) | 150 (67.3) |  |
| *AL133227.1* | ENSG00000273828 (%) | High | 74 (29.1) | 7 ( 22.6) | 67 (30.0) | 0.518 |
|  |  | Low | 180 (70.9) | 24 ( 77.4) | 156 (70.0) |  |
| *ADRA2B* | ENSG00000274286 (%) | High | 93 (36.6) | 17 ( 54.8) | 76 (34.1) | 0.04 |
|  |  | Low | 161 (63.4) | 14 ( 45.2) | 147 (65.9) |  |
| *AC132807.2* | ENSG00000274840 (%) | High | 58 (22.8) | 1 ( 3.2) | 57 (25.6) | 0.011 |
|  |  | Low | 196 (77.2) | 30 ( 96.8) | 166 (74.4) |  |
| *AP000676.3* | ENSG00000279793 (%) | High | 45 (17.7) | 7 ( 22.6) | 38 (17.0) | 0.613 |
|  |  | Low | 209 (82.3) | 24 ( 77.4) | 185 (83.0) |  |
| *NCOR1P4* | ENSG00000279864 (%) | High | 20 ( 7.9) | 3 ( 9.7) | 17 ( 7.6) | 0.966 |
|  |  | Low | 234 (92.1) | 28 ( 90.3) | 206 (92.4) |  |
| *AC022107.1* | ENSG00000280187 (%) | High | 105 (41.3) | 16 ( 51.6) | 89 (39.9) | 0.296 |
|  |  | Low | 149 (58.7) | 15 ( 48.4) | 134 (60.1) |  |
| *AL732314.4* | ENSG00000280767 (%) | High | 66 (26.0) | 11 ( 35.5) | 55 (24.7) | 0.285 |
|  |  | Low | 188 (74.0) | 20 ( 64.5) | 168 (75.3) |  |
| *GHET1* | ENSG00000281189 (%) | High | 95 (37.4) | 9 ( 29.0) | 86 (38.6) | 0.407 |
|  |  | Low | 159 (62.6) | 22 ( 71.0) | 137 (61.4) |  |
| *HELLPAR* | ENSG00000281344 (%) | High | 60 (23.6) | 9 ( 29.0) | 51 (22.9) | 0.595 |
|  |  | Low | 194 (76.4) | 22 ( 71.0) | 172 (77.1) |  |
| *AL033530.1* | ENSG00000285407 (%) | High | 50 (19.7) | 5 ( 16.1) | 45 (20.2) | 0.772 |
|  |  | Low | 204 (80.3) | 26 ( 83.9) | 178 (79.8) |  |
| *AL445238.1* | ENSG00000285566 (%) | High | 48 (18.9) | 5 ( 16.1) | 43 (19.3) | 0.861 |
|  |  | Low | 206 (81.1) | 26 ( 83.9) | 180 (80.7) |  |
| *AL365256.1* | ENSG00000286060 (%) | High | 60 (23.6) | 4 ( 12.9) | 56 (25.1) | 0.203 |
|  |  | Low | 194 (76.4) | 27 ( 87.1) | 167 (74.9) |  |
| *AC008945.2* | ENSG00000286271 (%) | High | 75 (29.5) | 9 ( 29.0) | 66 (29.6) | 1 |
|  |  | Low | 179 (70.5) | 22 ( 71.0) | 157 (70.4) |  |
| *AL022162.1* | ENSG00000287024 (%) | High | 88 (34.6) | 11 ( 35.5) | 77 (34.5) | 1 |
|  |  | Low | 166 (65.4) | 20 ( 64.5) | 146 (65.5) |  |
| *AC072028.1* | ENSG00000287045 (%) | High | 53 (20.9) | 3 ( 9.7) | 50 (22.4) | 0.161 |
|  |  | Low | 201 (79.1) | 28 ( 90.3) | 173 (77.6) |  |
| *AC006525.1* | ENSG00000287390 (%) | High | 59 (23.2) | 6 ( 19.4) | 53 (23.8) | 0.75 |
|  |  | Low | 195 (76.8) | 25 ( 80.6) | 170 (76.2) |  |
| *AC005865.2* | ENSG00000287712 (%) | High | 56 (22.0) | 7 ( 22.6) | 49 (22.0) | 1 |
|  |  | Low | 198 (78.0) | 24 ( 77.4) | 174 (78.0) |  |
| *AF241728.2* | ENSG00000287729 (%) | High | 78 (30.7) | 7 ( 22.6) | 71 (31.8) | 0.401 |
|  |  | Low | 176 (69.3) | 24 ( 77.4) | 152 (68.2) |  |
| *AL445524.2* | ENSG00000287856 (%) | High | 29 (11.4) | 1 ( 3.2) | 28 (12.6) | 0.219 |
|  |  | Low | 225 (88.6) | 30 ( 96.8) | 195 (87.4) |  |
| *AC114971.1* | ENSG00000287862 (%) | High | 44 (17.3) | 4 ( 12.9) | 40 (17.9) | 0.659 |
|  |  | Low | 210 (82.7) | 27 ( 87.1) | 183 (82.1) |  |
| *AL138701.2* | ENSG00000287923 (%) | High | 25 ( 9.8) | 1 ( 3.2) | 24 (10.8) | 0.318 |
|  |  | Low | 229 (90.2) | 30 ( 96.8) | 199 (89.2) |  |
| *AC115282.2* | ENSG00000288029 (%) | High | 26 (10.2) | 3 ( 9.7) | 23 (10.3) | 1 |
|  |  | Low | 228 (89.8) | 28 ( 90.3) | 200 (89.7) |  |
| *AC005618.4* | ENSG00000288095 (%) | High | 93 (36.6) | 9 ( 29.0) | 84 (37.7) | 0.462 |
|  |  | Low | 161 (63.4) | 22 ( 71.0) | 139 (62.3) |  |

(B)

| Gene_name |  | Level | Overall | Female | Male | p-value |
| --- | --- | --- | --- | --- | --- | --- |
| *HOXA11* | ENSG00000005073 (%) | High | 38 (15.0) | 13 (20.6) | 25 (13.1) | 0.21 |
|  |  | Low | 216 (85.0) | 50 (79.4) | 166 (86.9) |  |
| *TKTL1* | ENSG00000007350 (%) | High | 11 ( 4.3) | 4 ( 6.3) | 7 ( 3.7) | 0.582 |
|  |  | Low | 243 (95.7) | 59 (93.7) | 184 (96.3) |  |
| *PAX6* | ENSG00000007372 (%) | High | 36 (14.2) | 12 (19.0) | 24 (12.6) | 0.284 |
|  |  | Low | 218 (85.8) | 51 (81.0) | 167 (87.4) |  |
| *ZIC2* | ENSG00000043355 (%) | High | 78 (30.7) | 24 (38.1) | 54 (28.3) | 0.191 |
|  |  | Low | 176 (69.3) | 39 (61.9) | 137 (71.7) |  |
| *POLQ* | ENSG00000051341 (%) | High | 86 (33.9) | 29 (46.0) | 57 (29.8) | 0.028 |
|  |  | Low | 168 (66.1) | 34 (54.0) | 134 (70.2) |  |
| *CLDN18* | ENSG00000066405 (%) | High | 8 ( 3.1) | 4 ( 6.3) | 4 ( 2.1) | 0.207 |
|  |  | Low | 246 (96.9) | 59 (93.7) | 187 (97.9) |  |
| *PTGS2* | ENSG00000073756 (%) | High | 53 (20.9) | 17 (27.0) | 36 (18.8) | 0.23 |
|  |  | Low | 201 (79.1) | 46 (73.0) | 155 (81.2) |  |
| *CA12* | ENSG00000074410 (%) | High | 48 (18.9) | 12 (19.0) | 36 (18.8) | 1 |
|  |  | Low | 206 (81.1) | 51 (81.0) | 155 (81.2) |  |
| *CDH17* | ENSG00000079112 (%) | High | 15 ( 5.9) | 3 ( 4.8) | 12 ( 6.3) | 0.892 |
|  |  | Low | 239 (94.1) | 60 (95.2) | 179 (93.7) |  |
| *LYZ* | ENSG00000090382 (%) | High | 52 (20.5) | 10 (15.9) | 42 (22.0) | 0.388 |
|  |  | Low | 202 (79.5) | 53 (84.1) | 149 (78.0) |  |
| *SI* | ENSG00000090402 (%) | High | 10 ( 3.9) | 3 ( 4.8) | 7 ( 3.7) | 0.988 |
|  |  | Low | 244 (96.1) | 60 (95.2) | 184 (96.3) |  |
| *ORC6* | ENSG00000091651 (%) | High | 80 (31.5) | 28 (44.4) | 52 (27.2) | 0.017 |
|  |  | Low | 174 (68.5) | 35 (55.6) | 139 (72.8) |  |
| *Z83844.1* | ENSG00000100101 (%) | High | 16 ( 6.3) | 4 ( 6.3) | 12 ( 6.3) | 1 |
|  |  | Low | 238 (93.7) | 59 (93.7) | 179 (93.7) |  |
| *PVALB* | ENSG00000100362 (%) | High | 47 (18.5) | 13 (20.6) | 34 (17.8) | 0.753 |
|  |  | Low | 207 (81.5) | 50 (79.4) | 157 (82.2) |  |
| *CHGA* | ENSG00000100604 (%) | High | 22 ( 8.7) | 10 (15.9) | 12 ( 6.3) | 0.037 |
|  |  | Low | 232 (91.3) | 53 (84.1) | 179 (93.7) |  |
| *SMPD3* | ENSG00000103056 (%) | High | 68 (26.8) | 18 (28.6) | 50 (26.2) | 0.835 |
|  |  | Low | 186 (73.2) | 45 (71.4) | 141 (73.8) |  |
| *MEFV* | ENSG00000103313 (%) | High | 75 (29.5) | 24 (38.1) | 51 (26.7) | 0.119 |
|  |  | Low | 179 (70.5) | 39 (61.9) | 140 (73.3) |  |
| *RASL12* | ENSG00000103710 (%) | High | 82 (32.3) | 18 (28.6) | 64 (33.5) | 0.568 |
|  |  | Low | 172 (67.7) | 45 (71.4) | 127 (66.5) |  |
| *CCNE1* | ENSG00000105173 (%) | High | 42 (16.5) | 12 (19.0) | 30 (15.7) | 0.672 |
|  |  | Low | 212 (83.5) | 51 (81.0) | 161 (84.3) |  |
| *HAS1* | ENSG00000105509 (%) | High | 42 (16.5) | 14 (22.2) | 28 (14.7) | 0.228 |
|  |  | Low | 212 (83.5) | 49 (77.8) | 163 (85.3) |  |
| *FAM83E* | ENSG00000105523 (%) | High | 56 (22.0) | 16 (25.4) | 40 (20.9) | 0.573 |
|  |  | Low | 198 (78.0) | 47 (74.6) | 151 (79.1) |  |
| *TFPI2* | ENSG00000105825 (%) | High | 36 (14.2) | 16 (25.4) | 20 (10.5) | 0.006 |
|  |  | Low | 218 (85.8) | 47 (74.6) | 171 (89.5) |  |
| *ADAP1* | ENSG00000105963 (%) | High | 51 (20.1) | 14 (22.2) | 37 (19.4) | 0.758 |
|  |  | Low | 203 (79.9) | 49 (77.8) | 154 (80.6) |  |
| *SERPINE1* | ENSG00000106366 (%) | High | 85 (33.5) | 22 (34.9) | 63 (33.0) | 0.898 |
|  |  | Low | 169 (66.5) | 41 (65.1) | 128 (67.0) |  |
| *NPDC1* | ENSG00000107281 (%) | High | 85 (33.5) | 24 (38.1) | 61 (31.9) | 0.457 |
|  |  | Low | 169 (66.5) | 39 (61.9) | 130 (68.1) |  |
| *ASIC1* | ENSG00000110881 (%) | High | 68 (26.8) | 17 (27.0) | 51 (26.7) | 1 |
|  |  | Low | 186 (73.2) | 46 (73.0) | 140 (73.3) |  |
| *PCDHB2* | ENSG00000112852 (%) | High | 51 (20.1) | 22 (34.9) | 29 (15.2) | 0.001 |
|  |  | Low | 203 (79.9) | 41 (65.1) | 162 (84.8) |  |
| *PCDHB3* | ENSG00000113205 (%) | High | 42 (16.5) | 19 (30.2) | 23 (12.0) | 0.002 |
|  |  | Low | 212 (83.5) | 44 (69.8) | 168 (88.0) |  |
| *GABRG2* | ENSG00000113327 (%) | High | 31 (12.2) | 13 (20.6) | 18 ( 9.4) | 0.033 |
|  |  | Low | 223 (87.8) | 50 (79.4) | 173 (90.6) |  |
| *OTX1* | ENSG00000115507 (%) | High | 64 (25.2) | 26 (41.3) | 38 (19.9) | 0.001 |
|  |  | Low | 190 (74.8) | 37 (58.7) | 153 (80.1) |  |
| *APOA1* | ENSG00000118137 (%) | High | 104 (40.9) | 27 (42.9) | 77 (40.3) | 0.835 |
|  |  | Low | 150 (59.1) | 36 (57.1) | 114 (59.7) |  |
| *SPP1* | ENSG00000118785 (%) | High | 63 (24.8) | 16 (25.4) | 47 (24.6) | 1 |
|  |  | Low | 191 (75.2) | 47 (74.6) | 144 (75.4) |  |
| *CSF3R* | ENSG00000119535 (%) | High | 66 (26.0) | 25 (39.7) | 41 (21.5) | 0.007 |
|  |  | Low | 188 (74.0) | 38 (60.3) | 150 (78.5) |  |
| *PCDHB8* | ENSG00000120322 (%) | High | 42 (16.5) | 15 (23.8) | 27 (14.1) | 0.11 |
|  |  | Low | 212 (83.5) | 48 (76.2) | 164 (85.9) |  |
| *GUCY1B2* | ENSG00000123201 (%) | High | 56 (22.0) | 20 (31.7) | 36 (18.8) | 0.049 |
|  |  | Low | 198 (78.0) | 43 (68.3) | 155 (81.2) |  |
| *HJURP* | ENSG00000123485 (%) | High | 85 (33.5) | 31 (49.2) | 54 (28.3) | 0.004 |
|  |  | Low | 169 (66.5) | 32 (50.8) | 137 (71.7) |  |
| *NKX2-2* | ENSG00000125820 (%) | High | 17 ( 6.7) | 4 ( 6.3) | 13 ( 6.8) | 1 |
|  |  | Low | 237 (93.3) | 59 (93.7) | 178 (93.2) |  |
| *AHDC1* | ENSG00000126705 (%) | High | 99 (39.0) | 27 (42.9) | 72 (37.7) | 0.562 |
|  |  | Low | 155 (61.0) | 36 (57.1) | 119 (62.3) |  |
| *SIX1* | ENSG00000126778 (%) | High | 62 (24.4) | 19 (30.2) | 43 (22.5) | 0.291 |
|  |  | Low | 192 (75.6) | 44 (69.8) | 148 (77.5) |  |
| *DLGAP5* | ENSG00000126787 (%) | High | 78 (30.7) | 25 (39.7) | 53 (27.7) | 0.105 |
|  |  | Low | 176 (69.3) | 38 (60.3) | 138 (72.3) |  |
| *PZP* | ENSG00000126838 (%) | High | 26 (10.2) | 10 (15.9) | 16 ( 8.4) | 0.144 |
|  |  | Low | 228 (89.8) | 53 (84.1) | 175 (91.6) |  |
| *GAD1* | ENSG00000128683 (%) | High | 48 (18.9) | 16 (25.4) | 32 (16.8) | 0.182 |
|  |  | Low | 206 (81.1) | 47 (74.6) | 159 (83.2) |  |
| *PKDREJ* | ENSG00000130943 (%) | High | 66 (26.0) | 15 (23.8) | 51 (26.7) | 0.773 |
|  |  | Low | 188 (74.0) | 48 (76.2) | 140 (73.3) |  |
| *SPINK5* | ENSG00000133710 (%) | High | 64 (25.2) | 22 (34.9) | 42 (22.0) | 0.06 |
|  |  | Low | 190 (74.8) | 41 (65.1) | 149 (78.0) |  |
| *DTX1* | ENSG00000135144 (%) | High | 69 (27.2) | 19 (30.2) | 50 (26.2) | 0.651 |
|  |  | Low | 185 (72.8) | 44 (69.8) | 141 (73.8) |  |
| *IGF2BP3* | ENSG00000136231 (%) | High | 78 (30.7) | 32 (50.8) | 46 (24.1) | <0.001 |
|  |  | Low | 176 (69.3) | 31 (49.2) | 145 (75.9) |  |
| *ZIC5* | ENSG00000139800 (%) | High | 63 (24.8) | 23 (36.5) | 40 (20.9) | 0.021 |
|  |  | Low | 191 (75.2) | 40 (63.5) | 151 (79.1) |  |
| *GREB1L* | ENSG00000141449 (%) | High | 99 (39.0) | 25 (39.7) | 74 (38.7) | 1 |
|  |  | Low | 155 (61.0) | 38 (60.3) | 117 (61.3) |  |
| *NLRP12* | ENSG00000142405 (%) | High | 83 (32.7) | 19 (30.2) | 64 (33.5) | 0.736 |
|  |  | Low | 171 (67.3) | 44 (69.8) | 127 (66.5) |  |
| *CCNA2* | ENSG00000145386 (%) | High | 66 (26.0) | 21 (33.3) | 45 (23.6) | 0.171 |
|  |  | Low | 188 (74.0) | 42 (66.7) | 146 (76.4) |  |
| *FGD2* | ENSG00000146192 (%) | High | 88 (34.6) | 23 (36.5) | 65 (34.0) | 0.837 |
|  |  | Low | 166 (65.4) | 40 (63.5) | 126 (66.0) |  |
| *ASB15* | ENSG00000146809 (%) | High | 39 (15.4) | 8 (12.7) | 31 (16.2) | 0.636 |
|  |  | Low | 215 (84.6) | 55 (87.3) | 160 (83.8) |  |
| *NXF3* | ENSG00000147206 (%) | High | 33 (13.0) | 11 (17.5) | 22 (11.5) | 0.317 |
|  |  | Low | 221 (87.0) | 52 (82.5) | 169 (88.5) |  |
| *RGS20* | ENSG00000147509 (%) | High | 47 (18.5) | 14 (22.2) | 33 (17.3) | 0.491 |
|  |  | Low | 207 (81.5) | 49 (77.8) | 158 (82.7) |  |
| *ADIRF* | ENSG00000148671 (%) | High | 56 (22.0) | 7 (11.1) | 49 (25.7) | 0.025 |
|  |  | Low | 198 (78.0) | 56 (88.9) | 142 (74.3) |  |
| *CYP17A1* | ENSG00000148795 (%) | High | 44 (17.3) | 12 (19.0) | 32 (16.8) | 0.822 |
|  |  | Low | 210 (82.7) | 51 (81.0) | 159 (83.2) |  |
| *TLCD3B* | ENSG00000149926 (%) | High | 61 (24.0) | 14 (22.2) | 47 (24.6) | 0.83 |
|  |  | Low | 193 (76.0) | 49 (77.8) | 144 (75.4) |  |
| *HMGA2* | ENSG00000149948 (%) | High | 24 ( 9.4) | 11 (17.5) | 13 ( 6.8) | 0.024 |
|  |  | Low | 230 (90.6) | 52 (82.5) | 178 (93.2) |  |
| *SLC5A10* | ENSG00000154025 (%) | High | 33 (13.0) | 8 (12.7) | 25 (13.1) | 1 |
|  |  | Low | 221 (87.0) | 55 (87.3) | 166 (86.9) |  |
| *HOXB13* | ENSG00000159184 (%) | High | 18 ( 7.1) | 10 (15.9) | 8 ( 4.2) | 0.004 |
|  |  | Low | 236 (92.9) | 53 (84.1) | 183 (95.8) |  |
| *LY6E* | ENSG00000160932 (%) | High | 78 (30.7) | 22 (34.9) | 56 (29.3) | 0.498 |
|  |  | Low | 176 (69.3) | 41 (65.1) | 135 (70.7) |  |
| *FBXO27* | ENSG00000161243 (%) | High | 66 (26.0) | 18 (28.6) | 48 (25.1) | 0.708 |
|  |  | Low | 188 (74.0) | 45 (71.4) | 143 (74.9) |  |
| *ZSWIM5* | ENSG00000162415 (%) | High | 99 (39.0) | 31 (49.2) | 68 (35.6) | 0.077 |
|  |  | Low | 155 (61.0) | 32 (50.8) | 123 (64.4) |  |
| *XIRP2* | ENSG00000163092 (%) | High | 24 ( 9.4) | 8 (12.7) | 16 ( 8.4) | 0.442 |
|  |  | Low | 230 (90.6) | 55 (87.3) | 175 (91.6) |  |
| *CXCR1* | ENSG00000163464 (%) | High | 71 (28.0) | 18 (28.6) | 53 (27.7) | 1 |
|  |  | Low | 183 (72.0) | 45 (71.4) | 138 (72.3) |  |
| *EIF5A2* | ENSG00000163577 (%) | High | 82 (32.3) | 24 (38.1) | 58 (30.4) | 0.326 |
|  |  | Low | 172 (67.7) | 39 (61.9) | 133 (69.6) |  |
| *PPBP* | ENSG00000163736 (%) | High | 50 (19.7) | 10 (15.9) | 40 (20.9) | 0.487 |
|  |  | Low | 204 (80.3) | 53 (84.1) | 151 (79.1) |  |
| *TCF23* | ENSG00000163792 (%) | High | 74 (29.1) | 19 (30.2) | 55 (28.8) | 0.963 |
|  |  | Low | 180 (70.9) | 44 (69.8) | 136 (71.2) |  |
| *TMEM74* | ENSG00000164841 (%) | High | 73 (28.7) | 22 (34.9) | 51 (26.7) | 0.276 |
|  |  | Low | 181 (71.3) | 41 (65.1) | 140 (73.3) |  |
| *SLC16A9* | ENSG00000165449 (%) | High | 50 (19.7) | 14 (22.2) | 36 (18.8) | 0.688 |
|  |  | Low | 204 (80.3) | 49 (77.8) | 155 (81.2) |  |
| *C18orf54* | ENSG00000166845 (%) | High | 77 (30.3) | 26 (41.3) | 51 (26.7) | 0.043 |
|  |  | Low | 177 (69.7) | 37 (58.7) | 140 (73.3) |  |
| *KLK11* | ENSG00000167757 (%) | High | 23 ( 9.1) | 9 (14.3) | 14 ( 7.3) | 0.157 |
|  |  | Low | 231 (90.9) | 54 (85.7) | 177 (92.7) |  |
| *SIGLEC7* | ENSG00000168995 (%) | High | 84 (33.1) | 25 (39.7) | 59 (30.9) | 0.258 |
|  |  | Low | 170 (66.9) | 38 (60.3) | 132 (69.1) |  |
| *TMEM182* | ENSG00000170417 (%) | High | 60 (23.6) | 16 (25.4) | 44 (23.0) | 0.833 |
|  |  | Low | 194 (76.4) | 47 (74.6) | 147 (77.0) |  |
| *DPY19L2P2* | ENSG00000170629 (%) | High | 50 (19.7) | 15 (23.8) | 35 (18.3) | 0.443 |
|  |  | Low | 204 (80.3) | 48 (76.2) | 156 (81.7) |  |
| *TTLL6* | ENSG00000170703 (%) | High | 37 (14.6) | 13 (20.6) | 24 (12.6) | 0.171 |
|  |  | Low | 217 (85.4) | 50 (79.4) | 167 (87.4) |  |
| *CEACAM3* | ENSG00000170956 (%) | High | 74 (29.1) | 23 (36.5) | 51 (26.7) | 0.185 |
|  |  | Low | 180 (70.9) | 40 (63.5) | 140 (73.3) |  |
| *PKIA* | ENSG00000171033 (%) | High | 40 (15.7) | 20 (31.7) | 20 (10.5) | <0.001 |
|  |  | Low | 214 (84.3) | 43 (68.3) | 171 (89.5) |  |
| *FPR2* | ENSG00000171049 (%) | High | 70 (27.6) | 19 (30.2) | 51 (26.7) | 0.711 |
|  |  | Low | 184 (72.4) | 44 (69.8) | 140 (73.3) |  |
| *KRT20* | ENSG00000171431 (%) | High | 17 ( 6.7) | 5 ( 7.9) | 12 ( 6.3) | 0.869 |
|  |  | Low | 237 (93.3) | 58 (92.1) | 179 (93.7) |  |
| *ABCG4* | ENSG00000172350 (%) | High | 77 (30.3) | 22 (34.9) | 55 (28.8) | 0.448 |
|  |  | Low | 177 (69.7) | 41 (65.1) | 136 (71.2) |  |
| *GXYLT2* | ENSG00000172986 (%) | High | 60 (23.6) | 22 (34.9) | 38 (19.9) | 0.024 |
|  |  | Low | 194 (76.4) | 41 (65.1) | 153 (80.1) |  |
| *OLR1* | ENSG00000173391 (%) | High | 50 (19.7) | 15 (23.8) | 35 (18.3) | 0.443 |
|  |  | Low | 204 (80.3) | 48 (76.2) | 156 (81.7) |  |
| *MCMDC2* | ENSG00000178460 (%) | High | 88 (34.6) | 24 (38.1) | 64 (33.5) | 0.609 |
|  |  | Low | 166 (65.4) | 39 (61.9) | 127 (66.5) |  |
| *FOXS1* | ENSG00000179772 (%) | High | 96 (37.8) | 27 (42.9) | 69 (36.1) | 0.42 |
|  |  | Low | 158 (62.2) | 36 (57.1) | 122 (63.9) |  |
| *FAR2P1* | ENSG00000180178 (%) | High | 37 (14.6) | 17 (27.0) | 20 (10.5) | 0.003 |
|  |  | Low | 217 (85.4) | 46 (73.0) | 171 (89.5) |  |
| *ALX1* | ENSG00000180318 (%) | High | 25 ( 9.8) | 11 (17.5) | 14 ( 7.3) | 0.036 |
|  |  | Low | 229 (90.2) | 52 (82.5) | 177 (92.7) |  |
| *MAPK15* | ENSG00000181085 (%) | High | 33 (13.0) | 11 (17.5) | 22 (11.5) | 0.317 |
|  |  | Low | 221 (87.0) | 52 (82.5) | 169 (88.5) |  |
| *OFCC1* | ENSG00000181355 (%) | High | 28 (11.0) | 13 (20.6) | 15 ( 7.9) | 0.01 |
|  |  | Low | 226 (89.0) | 50 (79.4) | 176 (92.1) |  |
| *FDCSP* | ENSG00000181617 (%) | High | 12 ( 4.7) | 3 ( 4.8) | 9 ( 4.7) | 1 |
|  |  | Low | 242 (95.3) | 60 (95.2) | 182 (95.3) |  |
| *PLAG1* | ENSG00000181690 (%) | High | 79 (31.1) | 23 (36.5) | 56 (29.3) | 0.362 |
|  |  | Low | 175 (68.9) | 40 (63.5) | 135 (70.7) |  |
| *MACIR* | ENSG00000181751 (%) | High | 74 (29.1) | 22 (34.9) | 52 (27.2) | 0.314 |
|  |  | Low | 180 (70.9) | 41 (65.1) | 139 (72.8) |  |
| *C2CD4C* | ENSG00000183186 (%) | High | 80 (31.5) | 21 (33.3) | 59 (30.9) | 0.837 |
|  |  | Low | 174 (68.5) | 42 (66.7) | 132 (69.1) |  |
| *DUSP5P1* | ENSG00000183929 (%) | High | 50 (19.7) | 10 (15.9) | 40 (20.9) | 0.487 |
|  |  | Low | 204 (80.3) | 53 (84.1) | 151 (79.1) |  |
| *PKP3* | ENSG00000184363 (%) | High | 25 ( 9.8) | 9 (14.3) | 16 ( 8.4) | 0.262 |
|  |  | Low | 229 (90.2) | 54 (85.7) | 175 (91.6) |  |
| *MUC6* | ENSG00000184956 (%) | High | 14 ( 5.5) | 6 ( 9.5) | 8 ( 4.2) | 0.197 |
|  |  | Low | 240 (94.5) | 57 (90.5) | 183 (95.8) |  |
| *RFX6* | ENSG00000185002 (%) | High | 19 ( 7.5) | 11 (17.5) | 8 ( 4.2) | 0.001 |
|  |  | Low | 235 (92.5) | 52 (82.5) | 183 (95.8) |  |
| *ANKS1B* | ENSG00000185046 (%) | High | 62 (24.4) | 27 (42.9) | 35 (18.3) | <0.001 |
|  |  | Low | 192 (75.6) | 36 (57.1) | 156 (81.7) |  |
| *FOXI2* | ENSG00000186766 (%) | High | 74 (29.1) | 21 (33.3) | 53 (27.7) | 0.493 |
|  |  | Low | 180 (70.9) | 42 (66.7) | 138 (72.3) |  |
| *POTEG* | ENSG00000187537 (%) | High | 26 (10.2) | 12 (19.0) | 14 ( 7.3) | 0.015 |
|  |  | Low | 228 (89.8) | 51 (81.0) | 177 (92.7) |  |
| *DMBT1* | ENSG00000187908 (%) | High | 18 ( 7.1) | 8 (12.7) | 10 ( 5.2) | 0.086 |
|  |  | Low | 236 (92.9) | 55 (87.3) | 181 (94.8) |  |
| *IL1RAPL2* | ENSG00000189108 (%) | High | 66 (26.0) | 18 (28.6) | 48 (25.1) | 0.708 |
|  |  | Low | 188 (74.0) | 45 (71.4) | 143 (74.9) |  |
| *ALG1L* | ENSG00000189366 (%) | High | 84 (33.1) | 21 (33.3) | 63 (33.0) | 1 |
|  |  | Low | 170 (66.9) | 42 (66.7) | 128 (67.0) |  |
| *MYBPC1* | ENSG00000196091 (%) | High | 7 ( 2.8) | 4 ( 6.3) | 3 ( 1.6) | 0.118 |
|  |  | Low | 247 (97.2) | 59 (93.7) | 188 (98.4) |  |
| *H2AC11* | ENSG00000196787 (%) | High | 87 (34.3) | 27 (42.9) | 60 (31.4) | 0.132 |
|  |  | Low | 167 (65.7) | 36 (57.1) | 131 (68.6) |  |
| *ZNF679* | ENSG00000197123 (%) | High | 31 (12.2) | 7 (11.1) | 24 (12.6) | 0.933 |
|  |  | Low | 223 (87.8) | 56 (88.9) | 167 (87.4) |  |
| *CYP2B6* | ENSG00000197408 (%) | High | 82 (32.3) | 21 (33.3) | 61 (31.9) | 0.96 |
|  |  | Low | 172 (67.7) | 42 (66.7) | 130 (68.1) |  |
| *POTEH* | ENSG00000198062 (%) | High | 25 ( 9.8) | 12 (19.0) | 13 ( 6.8) | 0.01 |
|  |  | Low | 229 (90.2) | 51 (81.0) | 178 (93.2) |  |
| *STK39* | ENSG00000198648 (%) | High | 84 (33.1) | 19 (30.2) | 65 (34.0) | 0.68 |
|  |  | Low | 170 (66.9) | 44 (69.8) | 126 (66.0) |  |
| *EPS8L3* | ENSG00000198758 (%) | High | 68 (26.8) | 20 (31.7) | 48 (25.1) | 0.387 |
|  |  | Low | 186 (73.2) | 43 (68.3) | 143 (74.9) |  |
| *RN7SKP72* | ENSG00000199197 (%) | High | 43 (16.9) | 14 (22.2) | 29 (15.2) | 0.272 |
|  |  | Low | 211 (83.1) | 49 (77.8) | 162 (84.8) |  |
| *RNU5E-1* | ENSG00000199347 (%) | High | 66 (26.0) | 19 (30.2) | 47 (24.6) | 0.48 |
|  |  | Low | 188 (74.0) | 44 (69.8) | 144 (75.4) |  |
| *RNU5F-1* | ENSG00000199377 (%) | High | 41 (16.1) | 14 (22.2) | 27 (14.1) | 0.188 |
|  |  | Low | 213 (83.9) | 49 (77.8) | 164 (85.9) |  |
| *LINC02487* | ENSG00000203688 (%) | High | 64 (25.2) | 21 (33.3) | 43 (22.5) | 0.122 |
|  |  | Low | 190 (74.8) | 42 (66.7) | 148 (77.5) |  |
| *RIPPLY2* | ENSG00000203877 (%) | High | 28 (11.0) | 12 (19.0) | 16 ( 8.4) | 0.035 |
|  |  | Low | 226 (89.0) | 51 (81.0) | 175 (91.6) |  |
| *TRIM40* | ENSG00000204614 (%) | High | 38 (15.0) | 9 (14.3) | 29 (15.2) | 1 |
|  |  | Low | 216 (85.0) | 54 (85.7) | 162 (84.8) |  |
| *ASPDH* | ENSG00000204653 (%) | High | 93 (36.6) | 15 (23.8) | 78 (40.8) | 0.022 |
|  |  | Low | 161 (63.4) | 48 (76.2) | 113 (59.2) |  |
| *TMEM88B* | ENSG00000205116 (%) | High | 44 (17.3) | 10 (15.9) | 34 (17.8) | 0.874 |
|  |  | Low | 210 (82.7) | 53 (84.1) | 157 (82.2) |  |
| *AC108134.1* | ENSG00000205890 (%) | High | 36 (14.2) | 8 (12.7) | 28 (14.7) | 0.858 |
|  |  | Low | 218 (85.8) | 55 (87.3) | 163 (85.3) |  |
| *DEFA1* | ENSG00000206047 (%) | High | 46 (18.1) | 13 (20.6) | 33 (17.3) | 0.681 |
|  |  | Low | 208 (81.9) | 50 (79.4) | 158 (82.7) |  |
| *FOXL2NB* | ENSG00000206262 (%) | High | 46 (18.1) | 12 (19.0) | 34 (17.8) | 0.973 |
|  |  | Low | 208 (81.9) | 51 (81.0) | 157 (82.2) |  |
| *TRIM71* | ENSG00000206557 (%) | High | 58 (22.8) | 20 (31.7) | 38 (19.9) | 0.077 |
|  |  | Low | 196 (77.2) | 43 (68.3) | 153 (80.1) |  |
| *SETP21* | ENSG00000214244 (%) | High | 43 (16.9) | 12 (19.0) | 31 (16.2) | 0.746 |
|  |  | Low | 211 (83.1) | 51 (81.0) | 160 (83.8) |  |
| *PEX12P1* | ENSG00000215418 (%) | High | 41 (16.1) | 15 (23.8) | 26 (13.6) | 0.087 |
|  |  | Low | 213 (83.9) | 48 (76.2) | 165 (86.4) |  |
| *MCM3AP-AS1* | ENSG00000215424 (%) | High | 87 (34.3) | 27 (42.9) | 60 (31.4) | 0.132 |
|  |  | Low | 167 (65.7) | 36 (57.1) | 131 (68.6) |  |
| *OR7E104P* | ENSG00000219926 (%) | High | 66 (26.0) | 22 (34.9) | 44 (23.0) | 0.089 |
|  |  | Low | 188 (74.0) | 41 (65.1) | 147 (77.0) |  |
| *EBF2* | ENSG00000221818 (%) | High | 88 (34.6) | 19 (30.2) | 69 (36.1) | 0.477 |
|  |  | Low | 166 (65.4) | 44 (69.8) | 122 (63.9) |  |
| *MYBPHL* | ENSG00000221986 (%) | High | 41 (16.1) | 14 (22.2) | 27 (14.1) | 0.188 |
|  |  | Low | 213 (83.9) | 49 (77.8) | 164 (85.9) |  |
| *ATP5F1CP1* | ENSG00000224004 (%) | High | 34 (13.4) | 9 (14.3) | 25 (13.1) | 0.977 |
|  |  | Low | 220 (86.6) | 54 (85.7) | 166 (86.9) |  |
| *LINC00393* | ENSG00000224853 (%) | High | 7 ( 2.8) | 2 ( 3.2) | 5 ( 2.6) | 1 |
|  |  | Low | 247 (97.2) | 61 (96.8) | 186 (97.4) |  |
| *LINC01594* | ENSG00000225328 (%) | High | 36 (14.2) | 11 (17.5) | 25 (13.1) | 0.513 |
|  |  | Low | 218 (85.8) | 52 (82.5) | 166 (86.9) |  |
| *AL592114.2* | ENSG00000225522 (%) | High | 43 (16.9) | 10 (15.9) | 33 (17.3) | 0.949 |
|  |  | Low | 211 (83.1) | 53 (84.1) | 158 (82.7) |  |
| *AC018641.1* | ENSG00000226468 (%) | High | 45 (17.7) | 13 (20.6) | 32 (16.8) | 0.61 |
|  |  | Low | 209 (82.3) | 50 (79.4) | 159 (83.2) |  |
| *RPL39P40* | ENSG00000226580 (%) | High | 72 (28.3) | 14 (22.2) | 58 (30.4) | 0.279 |
|  |  | Low | 182 (71.7) | 49 (77.8) | 133 (69.6) |  |
| *AC092155.1* | ENSG00000226622 (%) | High | 56 (22.0) | 13 (20.6) | 43 (22.5) | 0.891 |
|  |  | Low | 198 (78.0) | 50 (79.4) | 148 (77.5) |  |
| *CYP4F29P* | ENSG00000228314 (%) | High | 54 (21.3) | 20 (31.7) | 34 (17.8) | 0.03 |
|  |  | Low | 200 (78.7) | 43 (68.3) | 157 (82.2) |  |
| *LINC02038* | ENSG00000229155 (%) | High | 58 (22.8) | 20 (31.7) | 38 (19.9) | 0.077 |
|  |  | Low | 196 (77.2) | 43 (68.3) | 153 (80.1) |  |
| *LINC01981* | ENSG00000229243 (%) | High | 45 (17.7) | 16 (25.4) | 29 (15.2) | 0.099 |
|  |  | Low | 209 (82.3) | 47 (74.6) | 162 (84.8) |  |
| *AL162582.1* | ENSG00000230234 (%) | High | 55 (21.7) | 5 ( 7.9) | 50 (26.2) | 0.004 |
|  |  | Low | 199 (78.3) | 58 (92.1) | 141 (73.8) |  |
| *AL356234.2* | ENSG00000230533 (%) | High | 67 (26.4) | 13 (20.6) | 54 (28.3) | 0.304 |
|  |  | Low | 187 (73.6) | 50 (79.4) | 137 (71.7) |  |
| *ARHGAP26-IT1* | ENSG00000230789 (%) | High | 83 (32.7) | 26 (41.3) | 57 (29.8) | 0.128 |
|  |  | Low | 171 (67.3) | 37 (58.7) | 134 (70.2) |  |
| *DPP4-DT* | ENSG00000230918 (%) | High | 64 (25.2) | 17 (27.0) | 47 (24.6) | 0.834 |
|  |  | Low | 190 (74.8) | 46 (73.0) | 144 (75.4) |  |
| *AL049734.2* | ENSG00000230973 (%) | High | 48 (18.9) | 22 (34.9) | 26 (13.6) | <0.001 |
|  |  | Low | 206 (81.1) | 41 (65.1) | 165 (86.4) |  |
| *LINC01249* | ENSG00000231532 (%) | High | 46 (18.1) | 13 (20.6) | 33 (17.3) | 0.681 |
|  |  | Low | 208 (81.9) | 50 (79.4) | 158 (82.7) |  |
| *AC005537.1* | ENSG00000232006 (%) | High | 43 (16.9) | 14 (22.2) | 29 (15.2) | 0.272 |
|  |  | Low | 211 (83.1) | 49 (77.8) | 162 (84.8) |  |
| *YWHAEP1* | ENSG00000232727 (%) | High | 47 (18.5) | 13 (20.6) | 34 (17.8) | 0.753 |
|  |  | Low | 207 (81.5) | 50 (79.4) | 157 (82.2) |  |
| *AL035401.1* | ENSG00000233358 (%) | High | 62 (24.4) | 20 (31.7) | 42 (22.0) | 0.163 |
|  |  | Low | 192 (75.6) | 43 (68.3) | 149 (78.0) |  |
| *FHAD1-AS1* | ENSG00000233485 (%) | High | 27 (10.6) | 9 (14.3) | 18 ( 9.4) | 0.395 |
|  |  | Low | 227 (89.4) | 54 (85.7) | 173 (90.6) |  |
| *AC008163.1* | ENSG00000233491 (%) | High | 29 (11.4) | 10 (15.9) | 19 ( 9.9) | 0.292 |
|  |  | Low | 225 (88.6) | 53 (84.1) | 172 (90.1) |  |
| *AC012146.1* | ENSG00000234327 (%) | High | 75 (29.5) | 22 (34.9) | 53 (27.7) | 0.356 |
|  |  | Low | 179 (70.5) | 41 (65.1) | 138 (72.3) |  |
| *C12orf75* | ENSG00000235162 (%) | High | 61 (24.0) | 18 (28.6) | 43 (22.5) | 0.42 |
|  |  | Low | 193 (76.0) | 45 (71.4) | 148 (77.5) |  |
| *AC023347.1* | ENSG00000235774 (%) | High | 57 (22.4) | 14 (22.2) | 43 (22.5) | 1 |
|  |  | Low | 197 (77.6) | 49 (77.8) | 148 (77.5) |  |
| *ELFN1-AS1* | ENSG00000236081 (%) | High | 43 (16.9) | 18 (28.6) | 25 (13.1) | 0.008 |
|  |  | Low | 211 (83.1) | 45 (71.4) | 166 (86.9) |  |
| *AL121970.1* | ENSG00000236389 (%) | High | 38 (15.0) | 14 (22.2) | 24 (12.6) | 0.097 |
|  |  | Low | 216 (85.0) | 49 (77.8) | 167 (87.4) |  |
| *RPSAP15* | ENSG00000237506 (%) | High | 91 (35.8) | 25 (39.7) | 66 (34.6) | 0.559 |
|  |  | Low | 163 (64.2) | 38 (60.3) | 125 (65.4) |  |
| *RN7SL751P* | ENSG00000240964 (%) | High | 69 (27.2) | 22 (34.9) | 47 (24.6) | 0.152 |
|  |  | Low | 185 (72.8) | 41 (65.1) | 144 (75.4) |  |
| *HOXA11-AS* | ENSG00000240990 (%) | High | 41 (16.1) | 17 (27.0) | 24 (12.6) | 0.012 |
|  |  | Low | 213 (83.9) | 46 (73.0) | 167 (87.4) |  |
| *ADAMTS9-AS1* | ENSG00000241158 (%) | High | 55 (21.7) | 15 (23.8) | 40 (20.9) | 0.762 |
|  |  | Low | 199 (78.3) | 48 (76.2) | 151 (79.1) |  |
| *RPLP0P2* | ENSG00000243742 (%) | High | 34 (13.4) | 10 (15.9) | 24 (12.6) | 0.649 |
|  |  | Low | 220 (86.6) | 53 (84.1) | 167 (87.4) |  |
| *GMCL2* | ENSG00000244234 (%) | High | 21 ( 8.3) | 8 (12.7) | 13 ( 6.8) | 0.227 |
|  |  | Low | 233 (91.7) | 55 (87.3) | 178 (93.2) |  |
| *IFITM10* | ENSG00000244242 (%) | High | 55 (21.7) | 17 (27.0) | 38 (19.9) | 0.313 |
|  |  | Low | 199 (78.3) | 46 (73.0) | 153 (80.1) |  |
| *AC046130.1* | ENSG00000245482 (%) | High | 35 (13.8) | 5 ( 7.9) | 30 (15.7) | 0.18 |
|  |  | Low | 219 (86.2) | 58 (92.1) | 161 (84.3) |  |
| *AC120114.1* | ENSG00000247735 (%) | High | 86 (33.9) | 26 (41.3) | 60 (31.4) | 0.201 |
|  |  | Low | 168 (66.1) | 37 (58.7) | 131 (68.6) |  |
| *AC021134.1* | ENSG00000248431 (%) | High | 31 (12.2) | 11 (17.5) | 20 (10.5) | 0.212 |
|  |  | Low | 223 (87.8) | 52 (82.5) | 171 (89.5) |  |
| *AC097534.1* | ENSG00000248774 (%) | High | 79 (31.1) | 21 (33.3) | 58 (30.4) | 0.776 |
|  |  | Low | 175 (68.9) | 42 (66.7) | 133 (69.6) |  |
| *UGT2A3P7* | ENSG00000248886 (%) | High | 79 (31.1) | 21 (33.3) | 58 (30.4) | 0.776 |
|  |  | Low | 175 (68.9) | 42 (66.7) | 133 (69.6) |  |
| *AC116049.1* | ENSG00000249413 (%) | High | 95 (37.4) | 30 (47.6) | 65 (34.0) | 0.075 |
|  |  | Low | 159 (62.6) | 33 (52.4) | 126 (66.0) |  |
| *AC008691.1* | ENSG00000249738 (%) | High | 54 (21.3) | 16 (25.4) | 38 (19.9) | 0.454 |
|  |  | Low | 200 (78.7) | 47 (74.6) | 153 (80.1) |  |
| *AC112178.1* | ENSG00000249803 (%) | High | 54 (21.3) | 16 (25.4) | 38 (19.9) | 0.454 |
|  |  | Low | 200 (78.7) | 47 (74.6) | 153 (80.1) |  |
| *AC114316.1* | ENSG00000250049 (%) | High | 77 (30.3) | 22 (34.9) | 55 (28.8) | 0.448 |
|  |  | Low | 177 (69.7) | 41 (65.1) | 136 (71.2) |  |
| *LINC02200* | ENSG00000250358 (%) | High | 38 (15.0) | 10 (15.9) | 28 (14.7) | 0.976 |
|  |  | Low | 216 (85.0) | 53 (84.1) | 163 (85.3) |  |
| *AACSP1* | ENSG00000250420 (%) | High | 21 ( 8.3) | 11 (17.5) | 10 ( 5.2) | 0.005 |
|  |  | Low | 233 (91.7) | 52 (82.5) | 181 (94.8) |  |
| *AC114786.2* | ENSG00000250612 (%) | High | 43 (16.9) | 13 (20.6) | 30 (15.7) | 0.477 |
|  |  | Low | 211 (83.1) | 50 (79.4) | 161 (84.3) |  |
| *LINC00491* | ENSG00000250682 (%) | High | 55 (21.7) | 23 (36.5) | 32 (16.8) | 0.002 |
|  |  | Low | 199 (78.3) | 40 (63.5) | 159 (83.2) |  |
| *MIR3945HG* | ENSG00000251230 (%) | High | 58 (22.8) | 18 (28.6) | 40 (20.9) | 0.281 |
|  |  | Low | 196 (77.2) | 45 (71.4) | 151 (79.1) |  |
| *AC135352.1* | ENSG00000251468 (%) | High | 77 (30.3) | 23 (36.5) | 54 (28.3) | 0.282 |
|  |  | Low | 177 (69.7) | 40 (63.5) | 137 (71.7) |  |
| *AC091133.5* | ENSG00000251550 (%) | High | 49 (19.3) | 18 (28.6) | 31 (16.2) | 0.049 |
|  |  | Low | 205 (80.7) | 45 (71.4) | 160 (83.8) |  |
| *HOXA10* | ENSG00000253293 (%) | High | 64 (25.2) | 24 (38.1) | 40 (20.9) | 0.011 |
|  |  | Low | 190 (74.8) | 39 (61.9) | 151 (79.1) |  |
| *AC105118.1* | ENSG00000253931 (%) | High | 15 ( 5.9) | 3 ( 4.8) | 12 ( 6.3) | 0.892 |
|  |  | Low | 239 (94.1) | 60 (95.2) | 179 (93.7) |  |
| *LINC02749* | ENSG00000254480 (%) | High | 41 (16.1) | 7 (11.1) | 34 (17.8) | 0.292 |
|  |  | Low | 213 (83.9) | 56 (88.9) | 157 (82.2) |  |
| *AC137894.1* | ENSG00000254739 (%) | High | 75 (29.5) | 17 (27.0) | 58 (30.4) | 0.726 |
|  |  | Low | 179 (70.5) | 46 (73.0) | 133 (69.6) |  |
| *CCDC84-DT* | ENSG00000255121 (%) | High | 76 (29.9) | 22 (34.9) | 54 (28.3) | 0.401 |
|  |  | Low | 178 (70.1) | 41 (65.1) | 137 (71.7) |  |
| *GPR142* | ENSG00000257008 (%) | High | 46 (18.1) | 12 (19.0) | 34 (17.8) | 0.973 |
|  |  | Low | 208 (81.9) | 51 (81.0) | 157 (82.2) |  |
| *MGAM2* | ENSG00000257743 (%) | High | 38 (15.0) | 18 (28.6) | 20 (10.5) | 0.001 |
|  |  | Low | 216 (85.0) | 45 (71.4) | 171 (89.5) |  |
| *AL355102.4* | ENSG00000258793 (%) | High | 67 (26.4) | 22 (34.9) | 45 (23.6) | 0.108 |
|  |  | Low | 187 (73.6) | 41 (65.1) | 146 (76.4) |  |
| *LINC02883* | ENSG00000259416 (%) | High | 80 (31.5) | 18 (28.6) | 62 (32.5) | 0.675 |
|  |  | Low | 174 (68.5) | 45 (71.4) | 129 (67.5) |  |
| *LINC02253* | ENSG00000259485 (%) | High | 27 (10.6) | 10 (15.9) | 17 ( 8.9) | 0.186 |
|  |  | Low | 227 (89.4) | 53 (84.1) | 174 (91.1) |  |
| *AC012236.1* | ENSG00000259772 (%) | High | 63 (24.8) | 15 (23.8) | 48 (25.1) | 0.966 |
|  |  | Low | 191 (75.2) | 48 (76.2) | 143 (74.9) |  |
| *LINC02562* | ENSG00000260265 (%) | High | 59 (23.2) | 19 (30.2) | 40 (20.9) | 0.183 |
|  |  | Low | 195 (76.8) | 44 (69.8) | 151 (79.1) |  |
| *AC012531.1* | ENSG00000260597 (%) | High | 36 (14.2) | 11 (17.5) | 25 (13.1) | 0.513 |
|  |  | Low | 218 (85.8) | 52 (82.5) | 166 (86.9) |  |
| *AC020658.3* | ENSG00000260648 (%) | High | 75 (29.5) | 20 (31.7) | 55 (28.8) | 0.775 |
|  |  | Low | 179 (70.5) | 43 (68.3) | 136 (71.2) |  |
| *AC106820.5* | ENSG00000260874 (%) | High | 92 (36.2) | 27 (42.9) | 65 (34.0) | 0.266 |
|  |  | Low | 162 (63.8) | 36 (57.1) | 126 (66.0) |  |
| *DOCK9-DT* | ENSG00000260992 (%) | High | 90 (35.4) | 26 (41.3) | 64 (33.5) | 0.335 |
|  |  | Low | 164 (64.6) | 37 (58.7) | 127 (66.5) |  |
| *AC132825.3* | ENSG00000261020 (%) | High | 34 (13.4) | 8 (12.7) | 26 (13.6) | 1 |
|  |  | Low | 220 (86.6) | 55 (87.3) | 165 (86.4) |  |
| *AC124312.3* | ENSG00000261069 (%) | High | 54 (21.3) | 17 (27.0) | 37 (19.4) | 0.27 |
|  |  | Low | 200 (78.7) | 46 (73.0) | 154 (80.6) |  |
| *AC133919.2* | ENSG00000261172 (%) | High | 69 (27.2) | 11 (17.5) | 58 (30.4) | 0.067 |
|  |  | Low | 185 (72.8) | 52 (82.5) | 133 (69.6) |  |
| *AC022167.4* | ENSG00000261481 (%) | High | 54 (21.3) | 9 (14.3) | 45 (23.6) | 0.167 |
|  |  | Low | 200 (78.7) | 54 (85.7) | 146 (76.4) |  |
| *FAM72C* | ENSG00000263513 (%) | High | 62 (24.4) | 18 (28.6) | 44 (23.0) | 0.473 |
|  |  | Low | 192 (75.6) | 45 (71.4) | 147 (77.0) |  |
| *AC007639.1* | ENSG00000263680 (%) | High | 62 (24.4) | 12 (19.0) | 50 (26.2) | 0.33 |
|  |  | Low | 192 (75.6) | 51 (81.0) | 141 (73.8) |  |
| *AC005324.5* | ENSG00000266261 (%) | High | 38 (15.0) | 10 (15.9) | 28 (14.7) | 0.976 |
|  |  | Low | 216 (85.0) | 53 (84.1) | 163 (85.3) |  |
| *RNVU1-31* | ENSG00000270722 (%) | High | 64 (25.2) | 18 (28.6) | 46 (24.1) | 0.586 |
|  |  | Low | 190 (74.8) | 45 (71.4) | 145 (75.9) |  |
| *AC244517.1* | ENSG00000272108 (%) | High | 40 (15.7) | 19 (30.2) | 21 (11.0) | 0.001 |
|  |  | Low | 214 (84.3) | 44 (69.8) | 170 (89.0) |  |
| *AC012087.2* | ENSG00000272554 (%) | High | 76 (29.9) | 26 (41.3) | 50 (26.2) | 0.035 |
|  |  | Low | 178 (70.1) | 37 (58.7) | 141 (73.8) |  |
| *AL022324.3* | ENSG00000272942 (%) | High | 55 (21.7) | 21 (33.3) | 34 (17.8) | 0.016 |
|  |  | Low | 199 (78.3) | 42 (66.7) | 157 (82.2) |  |
| *AP000553.2* | ENSG00000272954 (%) | High | 73 (28.7) | 25 (39.7) | 48 (25.1) | 0.04 |
|  |  | Low | 181 (71.3) | 38 (60.3) | 143 (74.9) |  |
| *AC100757.1* | ENSG00000273679 (%) | High | 81 (31.9) | 29 (46.0) | 52 (27.2) | 0.009 |
|  |  | Low | 173 (68.1) | 34 (54.0) | 139 (72.8) |  |
| *AL133227.1* | ENSG00000273828 (%) | High | 74 (29.1) | 18 (28.6) | 56 (29.3) | 1 |
|  |  | Low | 180 (70.9) | 45 (71.4) | 135 (70.7) |  |
| *ADRA2B* | ENSG00000274286 (%) | High | 93 (36.6) | 21 (33.3) | 72 (37.7) | 0.637 |
|  |  | Low | 161 (63.4) | 42 (66.7) | 119 (62.3) |  |
| *AC132807.2* | ENSG00000274840 (%) | High | 58 (22.8) | 22 (34.9) | 36 (18.8) | 0.014 |
|  |  | Low | 196 (77.2) | 41 (65.1) | 155 (81.2) |  |
| *AP000676.3* | ENSG00000279793 (%) | High | 45 (17.7) | 8 (12.7) | 37 (19.4) | 0.311 |
|  |  | Low | 209 (82.3) | 55 (87.3) | 154 (80.6) |  |
| *NCOR1P4* | ENSG00000279864 (%) | High | 20 ( 7.9) | 4 ( 6.3) | 16 ( 8.4) | 0.804 |
|  |  | Low | 234 (92.1) | 59 (93.7) | 175 (91.6) |  |
| *AC022107.1* | ENSG00000280187 (%) | High | 105 (41.3) | 27 (42.9) | 78 (40.8) | 0.893 |
|  |  | Low | 149 (58.7) | 36 (57.1) | 113 (59.2) |  |
| *AL732314.4* | ENSG00000280767 (%) | High | 66 (26.0) | 17 (27.0) | 49 (25.7) | 0.966 |
|  |  | Low | 188 (74.0) | 46 (73.0) | 142 (74.3) |  |
| *GHET1* | ENSG00000281189 (%) | High | 95 (37.4) | 30 (47.6) | 65 (34.0) | 0.075 |
|  |  | Low | 159 (62.6) | 33 (52.4) | 126 (66.0) |  |
| *HELLPAR* | ENSG00000281344 (%) | High | 60 (23.6) | 12 (19.0) | 48 (25.1) | 0.415 |
|  |  | Low | 194 (76.4) | 51 (81.0) | 143 (74.9) |  |
| *AL033530.1* | ENSG00000285407 (%) | High | 50 (19.7) | 16 (25.4) | 34 (17.8) | 0.258 |
|  |  | Low | 204 (80.3) | 47 (74.6) | 157 (82.2) |  |
| *AL445238.1* | ENSG00000285566 (%) | High | 48 (18.9) | 16 (25.4) | 32 (16.8) | 0.182 |
|  |  | Low | 206 (81.1) | 47 (74.6) | 159 (83.2) |  |
| *AL365256.1* | ENSG00000286060 (%) | High | 60 (23.6) | 25 (39.7) | 35 (18.3) | 0.001 |
|  |  | Low | 194 (76.4) | 38 (60.3) | 156 (81.7) |  |
| *AC008945.2* | ENSG00000286271 (%) | High | 75 (29.5) | 18 (28.6) | 57 (29.8) | 0.974 |
|  |  | Low | 179 (70.5) | 45 (71.4) | 134 (70.2) |  |
| *AL022162.1* | ENSG00000287024 (%) | High | 88 (34.6) | 14 (22.2) | 74 (38.7) | 0.025 |
|  |  | Low | 166 (65.4) | 49 (77.8) | 117 (61.3) |  |
| *AC072028.1* | ENSG00000287045 (%) | High | 53 (20.9) | 26 (41.3) | 27 (14.1) | <0.001 |
|  |  | Low | 201 (79.1) | 37 (58.7) | 164 (85.9) |  |
| *AC006525.1* | ENSG00000287390 (%) | High | 59 (23.2) | 14 (22.2) | 45 (23.6) | 0.963 |
|  |  | Low | 195 (76.8) | 49 (77.8) | 146 (76.4) |  |
| *AC005865.2* | ENSG00000287712 (%) | High | 56 (22.0) | 13 (20.6) | 43 (22.5) | 0.891 |
|  |  | Low | 198 (78.0) | 50 (79.4) | 148 (77.5) |  |
| *AF241728.2* | ENSG00000287729 (%) | High | 78 (30.7) | 25 (39.7) | 53 (27.7) | 0.105 |
|  |  | Low | 176 (69.3) | 38 (60.3) | 138 (72.3) |  |
| *AL445524.2* | ENSG00000287856 (%) | High | 29 (11.4) | 8 (12.7) | 21 (11.0) | 0.888 |
|  |  | Low | 225 (88.6) | 55 (87.3) | 170 (89.0) |  |
| *AC114971.1* | ENSG00000287862 (%) | High | 44 (17.3) | 11 (17.5) | 33 (17.3) | 1 |
|  |  | Low | 210 (82.7) | 52 (82.5) | 158 (82.7) |  |
| *AL138701.2* | ENSG00000287923 (%) | High | 25 ( 9.8) | 8 (12.7) | 17 ( 8.9) | 0.526 |
|  |  | Low | 229 (90.2) | 55 (87.3) | 174 (91.1) |  |
| *AC115282.2* | ENSG00000288029 (%) | High | 26 (10.2) | 10 (15.9) | 16 ( 8.4) | 0.144 |
|  |  | Low | 228 (89.8) | 53 (84.1) | 175 (91.6) |  |
| *AC005618.4* | ENSG00000288095 (%) | High | 93 (36.6) | 29 (46.0) | 64 (33.5) | 0.101 |
|  |  | Low | 161 (63.4) | 34 (54.0) | 127 (66.5) |  |

**Table S7**

**Table S7.** The details of fusion genes of 254 Taiwanese HCCs in our cohort.

| **sampleID** | **#FusionGene** | **LeftBreakpoint** | **RightBreakpoint** | **Gene1_CGT** | **Gene2_CGT** |
| --- | --- | --- | --- | --- | --- |
| C1000_RNA_HCC_933_T | *A2M--PZP* | chr12:9093465:- | chr12:9182117:- |  |  |
| C1000_RNA_HCC_908_T | *ABCC3--TMEM100* | chr17:50679899:+ | chr17:55721135:- |  |  |
| C1000_RNA_HCC_957_T | *ABLIM1--ATRNL1* | chr10:114487958:- | chr10:115394659:+ |  |  |
| C1000_RNA_HCC_940_T | *ABLIM1--GFRA1* | chr10:114631902:- | chr10:116125557:- |  |  |
| C1000_RNA_HCC_940_T | *ABLIM1--NRAP* | chr10:114631902:- | chr10:113640331:- |  |  |
| C1000_RNA_HCC_498_T | *ACADS--SPPL3* | chr12:120727189:+ | chr12:120791557:- |  |  |
| C1000_RNA_HCC_913_T | *ACER3--TSKU* | chr11:76861079:+ | chr11:76795609:+ |  |  |
| C1000_RNA_HCC_669_T | *ACSM2A--ACSM2B* | chr16:20486676:+ | chr16:20555476:- |  |  |
| C1000_RNA_HCC_558_T | *ACVR1B--ACVRL1* | chr12:51960187:+ | chr12:51918985:+ | TSG |  |
| C1000_RNA_HCC_893_T | *AFMID--SYNGR2* | chr17:78191060:+ | chr17:78170817:+ |  |  |
| C1000_RNA_HCC_946_T | *AGMO--DGKB* | chr7:15431005:- | chr7:14841450:- |  |  |
| C1000_RNA_HCC_969_T | *AKR1C3--AKR1C1* | chr10:5102357:+ | chr10:4982365:+ |  |  |
| C1000_RNA_HCC_623_T | *AKR1C4--AKR1C8P* | chr10:5200348:+ | chr10:5163020:- |  |  |
| C1000_RNA_HCC_648_T | *AL355315.1;HOGA1--UBTD1* | chr10:97584914:+ | chr10:97567914:+ |  |  |
| C1000_RNA_HCC_906_T | *AL358113.1;TJP2--PIP5K1B* | chr9:69240147:+ | chr9:68991140:+ |  |  |
| C1000_RNA_HCC_479_T | *ALDH2;AC002996.1--ACAD10* | chr12:111785346:+ | chr12:111733923:+ | CG |  |
| C1000_RNA_HCC_986_T | *AP000781.2;SLC43A3--SLC43A1* | chr11:57414615:- | chr11:57501329:- |  |  |
| C1000_RNA_HCC_906_T | *APOH--NKTR* | chr17:66223698:- | chr3:42642501:+ |  | CG |
| C1000_RNA_HCC_898_T | *ARF3--PRKAG1* | chr12:48956098:- | chr12:49005852:- |  |  |
| C1000_RNA_HCC_516_T | *ARHGEF10L--HEG1* | chr1:17621941:+ | chr3:125029488:- | TSG |  |
| C1000_RNA_HCC_672_T | *ATP5F1A--DYM* | chr18:46095053:- | chr18:49044204:- |  |  |
| C1000_RNA_HCC_482_T | *B4GALT1--RYR1* | chr9:33166758:- | chr19:38577918:+ |  |  |
| C1000_RNA_HCC_482_T | *B4GALT1--RYR1* | chr9:33166758:- | chr19:38578144:+ |  |  |
| C1000_RNA_HCC_691_T | *BAZ1B--MLXIPL* | chr7:73447264:- | chr7:73599695:- |  |  |
| C1000_RNA_HCC_506_T | *BIRC6--SPAST* | chr2:32465164:+ | chr2:32087492:+ | OG |  |
| C1000_RNA_HCC_750_T | *BIRC6--TTC27* | chr2:32473239:+ | chr2:32811024:+ | OG |  |
| C1000_RNA_HCC_726_T | *BRPF3--KHDRBS2* | chr6:36225364:+ | chr6:61732764:- |  |  |
| C1000_RNA_HCC_782_T | *C19orf12--ZNF875* | chr19:29715125:- | chr19:37322185:+ |  |  |
| C1000_RNA_HCC_672_T | *C1S--GNAI2* | chr12:7060877:+ | chr3:50252100:+ |  | CG |
| C1000_RNA_HCC_975_T | *CALU--FAM71F1* | chr7:128748804:+ | chr7:128726834:+ |  |  |
| C1000_RNA_HCC_728_T | *CARD10--RBPMS* | chr22:37496449:- | chr8:30570637:+ |  |  |
| C1000_RNA_HCC_913_T | *CCND1--FGF19* | chr11:69643991:+ | chr11:69703961:- | OG |  |
| C1000_RNA_HCC_913_T | *CCND1--FGF19* | chr11:69643991:+ | chr11:69703364:- | OG |  |
| C1000_RNA_HCC_916_T | *CDK13--SUGCT* | chr7:40063100:+ | chr7:40274513:+ |  |  |
| C1000_RNA_HCC_959_T | *CECR2--BCL2L13* | chr22:17477682:+ | chr22:17726677:+ |  |  |
| C1000_RNA_HCC_986_T | *CFHR5--CFHR2* | chr1:196996201:+ | chr1:196957891:+ |  |  |
| C1000_RNA_HCC_988_T | *CFLAR--NBEAL1* | chr2:201130146:+ | chr2:203144600:+ |  |  |
| C1000_RNA_HCC_722_T | *CGNL1--TCF12* | chr15:57407102:+ | chr15:57197773:+ |  | TSG |
| C1000_RNA_HCC_479_T | *CHCHD3--IMMP2L* | chr7:133070142:- | chr7:110663721:- |  |  |
| C1000_RNA_HCC_682_T | *CHP1--INO80* | chr15:41243739:+ | chr15:41096353:- |  | CG |
| C1000_RNA_HCC_929_T | *CLNS1A--RSF1* | chr11:77611315:- | chr11:77693611:- |  |  |
| C1000_RNA_HCC_1009_T | *CNST--FMO4* | chr1:246642037:+ | chr1:171331640:+ |  |  |
| C1000_RNA_HCC_906_T | *COBLL1--SLC38A11* | chr2:164841156:- | chr2:164939556:- |  |  |
| C1000_RNA_HCC_906_T | *COBLL1--SLC38A11* | chr2:164841156:- | chr2:164945727:- |  |  |
| C1000_RNA_HCC_741_T | *COPB2--MRPS22* | chr3:139366568:- | chr3:139350179:+ |  |  |
| C1000_RNA_HCC_908_T | *COX11--VMP1* | chr17:54963306:- | chr17:59764971:+ |  |  |
| C1000_RNA_HCC_983_T | *CPB2--LCP1* | chr13:46064648:- | chr13:46144520:- |  | CG |
| C1000_RNA_HCC_906_T | *CPEB3--IDE* | chr10:92290926:- | chr10:92487318:- | TSG |  |
| C1000_RNA_HCC_986_T | *CPT1A--AHNAK* | chr11:68794804:- | chr11:62491831:- |  |  |
| C1000_RNA_HCC_986_T | *CREBRF--ERGIC1* | chr5:173056479:+ | chr5:172888699:+ |  |  |
| C1000_RNA_HCC_682_T | *CTNNA1--KDM3B* | chr5:138827718:+ | chr5:138435620:+ | CG | CG |
| C1000_RNA_HCC_648_T | *CYB561A3--CPSF7* | chr11:61350991:- | chr11:61404704:- |  |  |
| C1000_RNA_HCC_675_T | *CYP2C19--CYP2C18;AL583836.1* | chr10:94775961:+ | chr10:94733297:+ | CG |  |
| C1000_RNA_HCC_891_T | *CYP3A5--CYP3A4* | chr7:99660499:- | chr7:99770235:- |  |  |
| C1000_RNA_HCC_891_T | *CYP3A5--TRIM4* | chr7:99660499:- | chr7:99903598:- |  |  |
| C1000_RNA_HCC_986_T | *DCHS2--FGA* | chr4:154489304:- | chr4:154589562:- |  |  |
| C1000_RNA_HCC_871_T | *DDX46--SEC24A* | chr5:134796150:+ | chr5:134674615:+ |  |  |
| C1000_RNA_HCC_883_T | *DNAJB1--PRKACA* | chr19:14518139:- | chr19:14107409:- | CG | OG |
| C1000_RNA_HCC_722_T | *ECE1--HP1BP3* | chr1:21272699:- | chr1:20776750:- |  |  |
| C1000_RNA_HCC_734_T | *EDC3--CHRNA3* | chr15:74640466:- | chr15:78602264:- |  |  |
| C1000_RNA_HCC_891_T | *EDC3--DUOXA1* | chr15:74655733:- | chr15:45123043:- |  |  |
| C1000_RNA_HCC_672_T | *EIF3L--JUND* | chr22:37851490:+ | chr19:18281105:- |  |  |
| C1000_RNA_HCC_482_T | *ELOVL5--GCLC* | chr6:53348817:- | chr6:53516222:- |  |  |
| C1000_RNA_HCC_894_T | *FBXW11--STK10* | chr5:171996890:- | chr5:172093960:- |  |  |
| C1000_RNA_HCC_941_T | *FDXR--ALDH1A2* | chr17:74872036:- | chr15:58420105:- |  |  |
| C1000_RNA_HCC_569_T | *FGA--FGG* | chr4:154590634:- | chr4:154605066:- |  |  |
| C1000_RNA_HCC_682_T | *FMC1-LUC7L2;FMC1--TMEM209* | chr7:139341522:+ | chr7:130206978:- |  |  |
| C1000_RNA_HCC_668_T | *FUT10--MTMR7* | chr8:33453216:- | chr8:17349081:- |  |  |
| C1000_RNA_HCC_799_T | *G3BP1--SAR1B* | chr5:151800346:+ | chr5:134612756:- |  |  |
| C1000_RNA_HCC_886_T | *GBF1--ELOVL3* | chr10:102245781:+ | chr10:102226432:+ |  |  |
| C1000_RNA_HCC_672_T | *GIGYF2--EFHD1* | chr2:232703489:+ | chr2:232681585:+ |  |  |
| C1000_RNA_HCC_1009_T | *GOLT1A--RXRG* | chr1:204213882:- | chr1:165428966:- |  |  |
| C1000_RNA_HCC_764_T | *GRAMD4--CERK* | chr22:46668908:+ | chr22:46701710:- |  |  |
| C1000_RNA_HCC_977_T | *IL1R1--IL1R2* | chr2:102154017:+ | chr2:102008515:+ |  |  |
| C1000_RNA_HCC_905_T | *IL6R--UBAP2L* | chr1:154454581:+ | chr1:154253900:+ |  |  |
| C1000_RNA_HCC_905_T | *IL6R--UBAP2L* | chr1:154454581:+ | chr1:154254836:+ |  |  |
| C1000_RNA_HCC_905_T | *IL6R--UBAP2L* | chr1:154454581:+ | chr1:154246204:+ |  |  |
| C1000_RNA_HCC_905_T | *IL6R--UBAP2L* | chr1:154449980:+ | chr1:154254836:+ |  |  |
| C1000_RNA_HCC_914_T | *INTS4--PRUNE2* | chr11:77955942:- | chr9:76854208:- |  |  |
| C1000_RNA_HCC_886_T | *IP6K1--UBA7* | chr3:49786354:- | chr3:49813953:- |  |  |
| C1000_RNA_HCC_672_T | *IRAK2--BRK1* | chr3:10178020:+ | chr3:10125626:+ |  |  |
| C1000_RNA_HCC_672_T | *ITCH--ASIP* | chr20:34369470:+ | chr20:34260365:+ |  |  |
| C1000_RNA_HCC_672_T | *ITCH--ASIP* | chr20:34369470:+ | chr20:34238860:+ |  |  |
| C1000_RNA_HCC_975_T | *ITCH--ASIP* | chr20:34408792:+ | chr20:34260365:+ |  |  |
| C1000_RNA_HCC_986_T | *ITPK1--MIPOL1* | chr14:92946331:- | chr14:37422855:+ |  |  |
| C1000_RNA_HCC_905_T | *JUP--ACLY* | chr17:41786588:- | chr17:41912542:- |  |  |
| C1000_RNA_HCC_908_T | *KANSL1--AC005324.3;CDRT1* | chr17:46082441:- | chr17:15615809:- | TSG |  |
| C1000_RNA_HCC_957_T | *KDM4B--ZFYVE9* | chr19:5138070:+ | chr1:52263773:+ |  |  |
| C1000_RNA_HCC_594_T | *KIAA1958--PLPP7* | chr9:112487118:+ | chr9:131307923:+ |  |  |
| C1000_RNA_HCC_594_T | *KIAA1958--SNX30* | chr9:112487118:+ | chr9:112864247:+ |  |  |
| C1000_RNA_HCC_648_T | *LARP1B--MFSD8* | chr4:128082305:+ | chr4:127949847:- |  |  |
| C1000_RNA_HCC_666_T | *LASP1--NCOR1* | chr17:38870258:+ | chr17:16158873:- | CG | TSG |
| C1000_RNA_HCC_986_T | *LIMD1--AC006059.2;HIGD1A* | chr3:45596287:+ | chr3:42786162:- |  |  |
| C1000_RNA_HCC_893_T | *LINC02203--AC134980.3* | chr15:21617244:+ | chr15:22030926:+ |  |  |
| C1000_RNA_HCC_986_T | *LPIN2--MYOM1* | chr18:2960649:- | chr18:3119995:- |  |  |
| C1000_RNA_HCC_570_T | *LPP--RNF139* | chr3:188225527:+ | chr8:124485831:+ | OG |  |
| C1000_RNA_HCC_954_T | *LRP5--CHKA* | chr11:68416527:+ | chr11:68097130:- | CG |  |
| C1000_RNA_HCC_929_T | *MALRD1--PLXDC2* | chr10:19790186:+ | chr10:20001775:+ |  |  |
| C1000_RNA_HCC_482_T | *MARS1--SPRYD4* | chr12:57490644:+ | chr12:56475168:+ |  |  |
| C1000_RNA_HCC_907_T | *MAST2--TMEM69* | chr1:45829581:+ | chr1:45693204:+ |  |  |
| C1000_RNA_HCC_506_T | *MAST3--GID4* | chr19:18151308:+ | chr17:18047366:+ |  |  |
| C1000_RNA_HCC_747_T | *MBNL2--TNFSF13B* | chr13:97276409:+ | chr13:108303253:+ |  |  |
| C1000_RNA_HCC_747_T | *MBNL2--TNFSF13B* | chr13:97276409:+ | chr13:108302776:+ |  |  |
| C1000_RNA_HCC_905_T | *MED15--COMT* | chr22:20507746:+ | chr22:19961199:+ |  |  |
| C1000_RNA_HCC_744_T | *MED1--NLRP13* | chr17:39424627:- | chr19:55892121:- |  |  |
| C1000_RNA_HCC_779_T | *MPHOSPH9--PITPNM2* | chr12:123214744:- | chr12:123010077:- |  |  |
| C1000_RNA_HCC_655_T | *MYH14--LRRC4B* | chr19:50252753:+ | chr19:50548873:- |  |  |
| C1000_RNA_HCC_648_T | *NDST1--CYTH4* | chr5:150535885:+ | chr22:37294660:+ |  |  |
| C1000_RNA_HCC_551_T | *NFIC--CELF5* | chr19:3435207:+ | chr19:3250985:+ |  |  |
| C1000_RNA_HCC_682_T | *NPLOC4--CCDC57* | chr17:81596116:- | chr17:82201952:- |  |  |
| C1000_RNA_HCC_682_T | *NPLOC4--CCDC57* | chr17:81596116:- | chr17:82208048:- |  |  |
| C1000_RNA_HCC_1009_T | *NR5A2--NFASC* | chr1:200120955:+ | chr1:204920632:+ |  |  |
| C1000_RNA_HCC_906_T | *NUCKS1--RAB29* | chr1:205719527:- | chr1:205772567:- |  |  |
| C1000_RNA_HCC_568_T | *PADI1--CROCC* | chr1:17232970:+ | chr1:16965723:+ |  |  |
| C1000_RNA_HCC_884_T | *PAH--C12orf42* | chr12:102894735:- | chr12:103263371:- |  |  |
| C1000_RNA_HCC_884_T | *PAH--C12orf42* | chr12:102894735:- | chr12:103237902:- |  |  |
| C1000_RNA_HCC_666_T | *PARD3B--DNER* | chr2:204686282:+ | chr2:229591888:- |  |  |
| C1000_RNA_HCC_799_T | *PARG--BMS1* | chr10:49885203:- | chr10:42791627:+ |  |  |
| C1000_RNA_HCC_498_T | *PDXDC1--AC138969.1* | chr16:15008847:+ | chr16:16344045:+ |  |  |
| C1000_RNA_HCC_691_T | *PEX5--EMG1* | chr12:7201841:+ | chr12:6975229:+ |  |  |
| C1000_RNA_HCC_648_T | *PI4KB--SELENBP1* | chr1:151326172:- | chr1:151372705:- |  |  |
| C1000_RNA_HCC_648_T | *PI4KB--SELENBP1* | chr1:151326172:- | chr1:151369769:- |  |  |
| C1000_RNA_HCC_672_T | *PIK3R1--NKD2* | chr5:68227009:+ | chr5:1032152:+ | TSG |  |
| C1000_RNA_HCC_779_T | *PLEKHA5--ATP5IF1* | chr12:19132450:+ | chr1:28246589:+ |  |  |
| C1000_RNA_HCC_906_T | *PLPP1--SLC38A9* | chr5:55534572:- | chr5:55649314:- |  |  |
| C1000_RNA_HCC_906_T | *PLPP1--SLC38A9* | chr5:55534572:- | chr5:55645895:- |  |  |
| C1000_RNA_HCC_708_T | *PPFIA1--RBM4;RBM14-RBM4* | chr11:70272436:+ | chr11:66639700:+ |  |  |
| C1000_RNA_HCC_691_T | *PPFIBP1--STK38L* | chr12:27524365:+ | chr12:27297710:+ | CG |  |
| C1000_RNA_HCC_862_T | *PPP3CC--COP1* | chr8:22533018:+ | chr1:175989479:- |  |  |
| C1000_RNA_HCC_928_T | *PTPRF--AL451062.3;ST3GAL3* | chr1:43569778:+ | chr1:43792102:+ |  |  |
| C1000_RNA_HCC_672_T | *QKI--CDC45* | chr6:163455421:+ | chr22:19499101:+ | Both |  |
| C1000_RNA_HCC_672_T | *QKI--CDC45* | chr6:163455421:+ | chr22:19505362:+ | Both |  |
| C1000_RNA_HCC_891_T | *RABEP2--ATP2A1* | chr16:28924403:- | chr16:28888787:+ |  |  |
| C1000_RNA_HCC_793_T | *RAPGEF6;AC008695.1--DACH2* | chr5:131592383:- | chrX:86651036:+ |  |  |
| C1000_RNA_HCC_682_T | *RAPH1--CYP20A1* | chr2:203489584:- | chr2:203266514:+ |  |  |
| C1000_RNA_HCC_682_T | *RAPH1--CYP20A1* | chr2:203470239:- | chr2:203266514:+ |  |  |
| C1000_RNA_HCC_891_T | *RFC1--UGDH* | chr4:39320383:- | chr4:39514184:- | CG |  |
| C1000_RNA_HCC_891_T | *RFC1--UGDH* | chr4:39320383:- | chr4:39514180:- | CG |  |
| C1000_RNA_HCC_647_T | *RHOA--IP6K1* | chr3:49411820:- | chr3:49738422:- | Both |  |
| C1000_RNA_HCC_954_T | *RHOA--MST1* | chr3:49375434:- | chr3:49687897:- | Both |  |
| C1000_RNA_HCC_954_T | *RHOA--MST1* | chr3:49373220:- | chr3:49687897:- | Both |  |
| C1000_RNA_HCC_583_T | *RND1--ARF3;AC073610.2* | chr12:48860997:- | chr12:48939108:- |  |  |
| C1000_RNA_HCC_676_T | *RNF138--RNF125* | chr18:32092886:+ | chr18:32068298:+ |  |  |
| C1000_RNA_HCC_746_T | *RNF138--RNF125* | chr18:32092886:+ | chr18:32068298:+ |  |  |
| C1000_RNA_HCC_570_T | *RNF139--LPP* | chr8:124475290:+ | chr3:188406112:+ |  | OG |
| C1000_RNA_HCC_891_T | *RNF213--SLC26A11* | chr17:80263778:+ | chr17:80236928:+ | CG |  |
| C1000_RNA_HCC_779_T | *RNGTT--PKNOX2* | chr6:88801564:- | chr11:125331819:+ |  |  |
| C1000_RNA_HCC_722_T | *RPL12--NIBAN2* | chr9:127451281:- | chr9:127531778:- |  |  |
| C1000_RNA_HCC_912_T | *RPS6KB1;AC005702.1--NSF* | chr17:59945518:+ | chr17:46704759:+ |  |  |
| C1000_RNA_HCC_906_T | *RXRA--RAPGEF1* | chr9:134434207:+ | chr9:131650949:- | CG |  |
| C1000_RNA_HCC_986_T | *RXRA--WDR5* | chr9:134326659:+ | chr9:134155693:+ | CG |  |
| C1000_RNA_HCC_986_T | *RXRA--WDR5* | chr9:134326659:+ | chr9:134157893:+ | CG |  |
| C1000_RNA_HCC_594_T | *SEC16A--NOTCH1* | chr9:136462887:- | chr9:136544102:- |  | Both |
| C1000_RNA_HCC_612_T | *SETD5--ITPR1* | chr3:9453868:+ | chr3:4699813:+ |  |  |
| C1000_RNA_HCC_616_T | *SHC1--ENAH* | chr1:154969378:- | chr1:225508017:- |  |  |
| C1000_RNA_HCC_986_T | *SHMT1--SHROOM3* | chr17:18347496:- | chr4:76770626:+ |  |  |
| C1000_RNA_HCC_906_T | *SLC25A25--ASH1L* | chr9:128068580:+ | chr1:155439068:- |  |  |
| C1000_RNA_HCC_482_T | *SLC25A27--UBR2* | chr6:46671228:+ | chr6:42573734:+ |  |  |
| C1000_RNA_HCC_1008_T | *SLC30A7--DPYD* | chr1:100921841:+ | chr1:97883374:- |  | CG |
| C1000_RNA_HCC_906_T | *SLC35D1--C1orf141* | chr1:67042236:- | chr1:67096321:- |  |  |
| C1000_RNA_HCC_682_T | *SLC38A10--CCDC57* | chr17:81289691:- | chr17:82163357:- |  |  |
| C1000_RNA_HCC_531_T | *SLC45A2--AMACR;C1QTNF3-AMACR* | chr5:33982236:- | chr5:34005899:- |  |  |
| C1000_RNA_HCC_551_T | *SLC45A2--AMACR;C1QTNF3-AMACR* | chr5:33982236:- | chr5:34005899:- |  |  |
| C1000_RNA_HCC_559_T | *SLC45A2--AMACR;C1QTNF3-AMACR* | chr5:33982236:- | chr5:34005899:- |  |  |
| C1000_RNA_HCC_599_T | *SLC45A2--AMACR;C1QTNF3-AMACR* | chr5:33982236:- | chr5:34005899:- |  |  |
| C1000_RNA_HCC_755_T | *SLC45A2--AMACR;C1QTNF3-AMACR* | chr5:33982236:- | chr5:34005899:- |  |  |
| C1000_RNA_HCC_479_T | *SPATA6L--INSL6* | chr9:4600653:- | chr9:5164265:- |  |  |
| C1000_RNA_HCC_482_T | *SREBF2--TEF* | chr22:41833358:+ | chr22:41387351:+ |  |  |
| C1000_RNA_HCC_770_T | *SRSF3--PNPLA1* | chr6:36594481:+ | chr6:36291320:+ | OG |  |
| C1000_RNA_HCC_588_T | *ST7--CAPZA2* | chr7:116953691:+ | chr7:116916060:+ |  |  |
| C1000_RNA_HCC_747_T | *STAMBPL1--LIPM* | chr10:88916817:+ | chr10:88802939:+ |  |  |
| C1000_RNA_HCC_568_T | *STX12--FAM76A* | chr1:27789631:+ | chr1:27755195:+ |  |  |
| C1000_RNA_HCC_583_T | *TBC1D14--KIAA0232* | chr4:6967424:+ | chr4:6880787:+ |  |  |
| C1000_RNA_HCC_672_T | *TBCD--CCN4* | chr17:82884202:+ | chr8:133212864:+ |  |  |
| C1000_RNA_HCC_672_T | *TCF12--TMOD3* | chr15:56921098:+ | chr15:51916015:+ | TSG |  |
| C1000_RNA_HCC_672_T | *TCF12--TMOD3* | chr15:56921098:+ | chr15:51934250:+ | TSG |  |
| C1000_RNA_HCC_962_T | *TIAM2--SCAF8* | chr6:154860998:+ | chr6:154773989:+ |  |  |
| C1000_RNA_HCC_583_T | *TJP2--UQCR10* | chr9:69174432:+ | chr22:29769678:+ |  |  |
| C1000_RNA_HCC_516_T | *TLK1--SCTR* | chr2:171160290:- | chr2:119441599:- |  |  |
| C1000_RNA_HCC_516_T | *TLK1--SCTR* | chr2:171160290:- | chr2:119446885:- |  |  |
| C1000_RNA_HCC_682_T | *TMEM63A--LIN9* | chr1:225877395:- | chr1:226266332:- |  |  |
| C1000_RNA_HCC_684_T | *TSTD3--MAP7* | chr6:99531790:+ | chr6:136411697:- |  |  |
| C1000_RNA_HCC_653_T | *TTPAL--HNF4A* | chr20:44486706:+ | chr20:44431175:+ |  |  |
| C1000_RNA_HCC_891_T | *UBE2H--PDK4* | chr7:129951278:- | chr7:95595164:- |  |  |
| C1000_RNA_HCC_891_T | *UBE2H--PDK4* | chr7:129952881:- | chr7:95595164:- |  |  |
| C1000_RNA_HCC_734_T | *UBE2Q1--TDRD10* | chr1:154551397:- | chr1:154544860:+ |  |  |
| C1000_RNA_HCC_558_T | *UBL3--SYNE1* | chr13:29849512:- | chr6:152381362:- |  | CG |
| C1000_RNA_HCC_682_T | *UBXN6--MPND* | chr19:4452364:- | chr19:4352897:+ |  |  |
| C1000_RNA_HCC_916_T | *UHRF1BP1L--ANKS1B* | chr12:100142592:- | chr12:99825389:- |  |  |
| C1000_RNA_HCC_588_T | *USP14--THOC1* | chr18:180339:+ | chr18:226901:- |  |  |
| C1000_RNA_HCC_912_T | *USP22--GRAP* | chr17:21015752:- | chr17:19024383:- |  |  |
| C1000_RNA_HCC_786_T | *UVSSA--ESRRG* | chr4:1366431:+ | chr1:216677491:- |  |  |
| C1000_RNA_HCC_988_T | *VAV2--AP003419.1;POLD4* | chr9:133939103:- | chr11:67352019:- |  |  |
| C1000_RNA_HCC_583_T | *WAC--KIAA1217* | chr10:28589851:+ | chr10:23877406:+ |  |  |
| C1000_RNA_HCC_906_T | *WDFY2--SERPINE3* | chr13:51584824:+ | chr13:51355043:+ |  |  |
| C1000_RNA_HCC_908_T | *WDR45B--ANKFN1* | chr17:82643949:- | chr17:56440327:+ |  |  |
| C1000_RNA_HCC_779_T | *WNK1--ERC1* | chr12:894635:+ | chr12:1141620:+ |  | CG |
| C1000_RNA_HCC_988_T | *XPR1--RASAL2* | chr1:180682411:+ | chr1:178473075:+ |  |  |
| C1000_RNA_HCC_972_T | *XRRA1--NXPE2* | chr11:74921214:- | chr11:114679657:+ |  |  |
| C1000_RNA_HCC_495_T | *ZBTB1--SYNE2* | chr14:64516751:+ | chr14:64174944:+ |  |  |
| C1000_RNA_HCC_506_T | *ZBTB25--PPP4R4* | chr14:64487114:- | chr14:94227275:+ |  |  |
| C1000_RNA_HCC_482_T | *ZC3H7B--EP300* | chr22:41351646:+ | chr22:41176247:+ |  | TSG |
| C1000_RNA_HCC_906_T | *ZCCHC14--TANGO6* | chr16:87460008:- | chr16:68974028:+ |  |  |
| C1000_RNA_HCC_910_T | *ZNF490--TECR* | chr19:12597149:- | chr19:14562525:+ |  |  |
| C1000_RNA_HCC_551_T | *ZNF512B--SYCP2* | chr20:63959918:- | chr20:59882165:- |  |  |
| C1000_RNA_HCC_672_T | *ZNF592--GPR37L1* | chr15:84778312:+ | chr1:202127741:+ |  |  |

**Table S8**

**Table S8.** The details of novel fusion genes of 254 Taiwanese HCCs in our cohort.

| **Group** | **#FusionGene** | **head group** | **tail group** | **sample count** |
| --- | --- | --- | --- | --- |
| Novel-111 | *ABCC3--TMEM100* |  |  | 1 |
| Novel-111 | *ABLIM1--ATRNL1* |  |  | 1 |
| Novel-111 | *ABLIM1--GFRA1* |  |  | 1 |
| Novel-111 | *ABLIM1--NRAP* |  |  | 1 |
| Novel-111 | *AGMO--DGKB* |  |  | 1 |
| Novel-111 | *AKR1C4--AKR1C8P* |  |  | 1 |
| Novel-111 | *APOH--NKTR* |  | CG | 1 |
| Novel-111 | *ARF3--PRKAG1* |  |  | 1 |
| Novel-111 | *ARHGEF10L--HEG1* | TSG |  | 1 |
| Novel-111 | *ATP5F1A--DYM* |  |  | 1 |
| Novel-111 | *B4GALT1--RYR1* |  |  | 1 |
| Novel-111 | *BRPF3--KHDRBS2* |  |  | 1 |
| Novel-111 | *C19orf12--ZNF875* |  |  | 1 |
| Novel-111 | *C1S--GNAI2* |  | CG | 1 |
| Novel-111 | *CALU--FAM71F1* |  |  | 1 |
| Novel-111 | *CARD10--RBPMS* |  |  | 1 |
| Novel-111 | *CCND1--FGF19* | OG |  | 1 |
| Novel-111 | *CFLAR--NBEAL1* |  |  | 1 |
| Novel-111 | *CHCHD3--IMMP2L* |  |  | 1 |
| Novel-111 | *CHP1--INO80* |  | CG | 1 |
| Novel-111 | *CNST--FMO4* |  |  | 1 |
| Novel-111 | *COX11--VMP1* |  |  | 1 |
| Novel-111 | *CPB2--LCP1* |  | CG | 1 |
| Novel-111 | *CPT1A--AHNAK* |  |  | 1 |
| Novel-111 | *CREBRF--ERGIC1* |  |  | 1 |
| Novel-111 | *CYB561A3--CPSF7* |  |  | 1 |
| Novel-111 | *CYP3A5--TRIM4* |  |  | 1 |
| Novel-111 | *DCHS2--FGA* |  |  | 1 |
| Novel-111 | *EDC3--CHRNA3* |  |  | 1 |
| Novel-111 | *EDC3--DUOXA1* |  |  | 1 |
| Novel-111 | *EIF3L--JUND* |  |  | 1 |
| Novel-111 | *ELOVL5--GCLC* |  |  | 1 |
| Novel-111 | *FDXR--ALDH1A2* |  |  | 1 |
| Novel-111 | *FMC1-LUC7L2;FMC1--TMEM209* |  |  | 1 |
| Novel-111 | *FUT10--MTMR7* |  |  | 1 |
| Novel-111 | *G3BP1--SAR1B* |  |  | 1 |
| Novel-111 | *GBF1--ELOVL3* |  |  | 1 |
| Novel-111 | *GOLT1A--RXRG* |  |  | 1 |
| Novel-111 | *GRAMD4--CERK* |  |  | 1 |
| Novel-111 | *IL6R--UBAP2L* |  |  | 1 |
| Novel-111 | *INTS4--PRUNE2* |  |  | 1 |
| Novel-111 | *KANSL1--AC005324.3;CDRT1* | TSG |  | 1 |
| Novel-111 | *KDM4B--ZFYVE9* |  |  | 1 |
| Novel-111 | *KIAA1958--PLPP7* |  |  | 1 |
| Novel-111 | *LARP1B--MFSD8* |  |  | 1 |
| Novel-111 | *LASP1--NCOR1* | CG | TSG | 1 |
| Novel-111 | *LIMD1--AC006059.2;HIGD1A* |  |  | 1 |
| Novel-111 | *LINC02203--AC134980.3* |  |  | 1 |
| Novel-111 | *LPP--RNF139* | OG |  | 1 |
| Novel-111 | *MARS1--SPRYD4* |  |  | 1 |
| Novel-111 | *MAST3--GID4* |  |  | 1 |
| Novel-111 | *MBNL2--TNFSF13B* |  |  | 1 |
| Novel-111 | *MED1--NLRP13* |  |  | 1 |
| Novel-111 | *MYH14--LRRC4B* |  |  | 1 |
| Novel-111 | *NDST1--CYTH4* |  |  | 1 |
| Novel-111 | *NR5A2--NFASC* |  |  | 1 |
| Novel-111 | *NUCKS1--RAB29* |  |  | 1 |
| Novel-111 | *PADI1--CROCC* |  |  | 1 |
| Novel-111 | *PAH--C12orf42* |  |  | 1 |
| Novel-111 | *PARD3B--DNER* |  |  | 1 |
| Novel-111 | *PDXDC1--AC138969.1* |  |  | 1 |
| Novel-111 | *PEX5--EMG1* |  |  | 1 |
| Novel-111 | *PIK3R1--NKD2* | TSG |  | 1 |
| Novel-111 | *PLEKHA5--ATP5IF1* |  |  | 1 |
| Novel-111 | *PPP3CC--COP1* |  |  | 1 |
| Novel-111 | *QKI--CDC45* | Both |  | 1 |
| Novel-111 | *RABEP2--ATP2A1* |  |  | 1 |
| Novel-111 | *RAPGEF6;AC008695.1--DACH2* |  |  | 1 |
| Novel-111 | *RAPH1--CYP20A1* |  |  | 1 |
| Novel-111 | *RHOA--MST1* | Both |  | 1 |
| Novel-111 | *RND1--ARF3;AC073610.2* |  |  | 1 |
| Novel-111 | *RNF139--LPP* |  | OG | 1 |
| Novel-111 | *RNGTT--PKNOX2* |  |  | 1 |
| Novel-111 | *RPL12--NIBAN2* |  |  | 1 |
| Novel-111 | *RPS6KB1;AC005702.1--NSF* |  |  | 1 |
| Novel-111 | *RXRA--RAPGEF1* | CG |  | 1 |
| Novel-111 | *SETD5--ITPR1* |  |  | 1 |
| Novel-111 | *SHC1--ENAH* |  |  | 1 |
| Novel-111 | *SHMT1--SHROOM3* |  |  | 1 |
| Novel-111 | *SLC25A25--ASH1L* |  |  | 1 |
| Novel-111 | *SLC25A27--UBR2* |  |  | 1 |
| Novel-111 | *SLC30A7--DPYD* |  | CG | 1 |
| Novel-111 | *SLC35D1--C1orf141* |  |  | 1 |
| Novel-111 | *SPATA6L--INSL6* |  |  | 1 |
| Novel-111 | *SREBF2--TEF* |  |  | 1 |
| Novel-111 | *SRSF3--PNPLA1* | OG |  | 1 |
| Novel-111 | *STAMBPL1--LIPM* |  |  | 1 |
| Novel-111 | *STX12--FAM76A* |  |  | 1 |
| Novel-111 | *TBCD--CCN4* |  |  | 1 |
| Novel-111 | *TCF12--TMOD3* | TSG |  | 1 |
| Novel-111 | *TJP2--UQCR10* |  |  | 1 |
| Novel-111 | *TLK1--SCTR* |  |  | 1 |
| Novel-111 | *TMEM63A--LIN9* |  |  | 1 |
| Novel-111 | *TSTD3--MAP7* |  |  | 1 |
| Novel-111 | *UBE2H--PDK4* |  |  | 1 |
| Novel-111 | *UBE2Q1--TDRD10* |  |  | 1 |
| Novel-111 | *UBL3--SYNE1* |  | CG | 1 |
| Novel-111 | *UBXN6--MPND* |  |  | 1 |
| Novel-111 | *USP22--GRAP* |  |  | 1 |
| Novel-111 | *UVSSA--ESRRG* |  |  | 1 |
| Novel-111 | *VAV2--AP003419.1;POLD4* |  |  | 1 |
| Novel-111 | *WAC--KIAA1217* |  |  | 1 |
| Novel-111 | *WDR45B--ANKFN1* |  |  | 1 |
| Novel-111 | *XPR1--RASAL2* |  |  | 1 |
| Novel-111 | *XRRA1--NXPE2* |  |  | 1 |
| Novel-111 | *ZBTB1--SYNE2* |  |  | 1 |
| Novel-111 | *ZBTB25--PPP4R4* |  |  | 1 |
| Novel-111 | *ZCCHC14--TANGO6* |  |  | 1 |
| Novel-111 | *ZNF490--TECR* |  |  | 1 |
| Novel-111 | *ZNF512B--SYCP2* |  |  | 1 |
| Novel-111 | *ZNF592--GPR37L1* |  |  | 1 |
| Novel-actifact-7 | *ACSM2A--ACSM2B* |  |  | 1 |
| Novel-actifact-7 | *AKR1C3--AKR1C1* |  |  | 1 |
| Novel-actifact-7 | *AP000781.2;SLC43A3--SLC43A1* |  |  | 1 |
| Novel-actifact-7 | *CFHR5--CFHR2* |  |  | 1 |
| Novel-actifact-7 | *CYP2C19--CYP2C18;AL583836.1* | CG |  | 1 |
| Novel-actifact-7 | *CYP3A5--CYP3A4* |  |  | 1 |
| Novel-actifact-7 | *FGA--FGG* |  |  | 1 |
| Novel-reverse-HEAD-TAIL-12 | *ACADS--SPPL3* |  |  | 1 |
| Novel-reverse-HEAD-TAIL-12 | *ACER3--TSKU* |  |  | 1 |
| Novel-reverse-HEAD-TAIL-12 | *CECR2--BCL2L13* |  |  | 1 |
| Novel-reverse-HEAD-TAIL-12 | *CLNS1A--RSF1* |  |  | 1 |
| Novel-reverse-HEAD-TAIL-12 | *MAST2--TMEM69* |  |  | 1 |
| Novel-reverse-HEAD-TAIL-12 | *MPHOSPH9--PITPNM2* |  |  | 1 |
| Novel-reverse-HEAD-TAIL-12 | *NPLOC4--CCDC57* |  |  | 1 |
| Novel-reverse-HEAD-TAIL-12 | *RFC1--UGDH* | CG |  | 1 |
| Novel-reverse-HEAD-TAIL-12 | *RHOA--IP6K1* | Both |  | 1 |
| Novel-reverse-HEAD-TAIL-12 | *SLC38A10--CCDC57* |  |  | 1 |
| Novel-reverse-HEAD-TAIL-12 | *TTPAL--HNF4A* |  |  | 1 |
| Novel-reverse-HEAD-TAIL-12 | *ZC3H7B--EP300* |  | TSG | 1 |
| Reported-43 | *AFMID--SYNGR2* |  |  | 1 |
| Reported-43 | *AL355315.1;HOGA1--UBTD1* |  |  | 1 |
| Reported-43 | *ALDH2;AC002996.1--ACAD10* | CG |  | 1 |
| Reported-43 | *BAZ1B--MLXIPL* |  |  | 1 |
| Reported-43 | *BIRC6--SPAST* | OG |  | 1 |
| Reported-43 | *BIRC6--TTC27* | OG |  | 1 |
| Reported-43 | *CDK13--SUGCT* |  |  | 1 |
| Reported-43 | *CGNL1--TCF12* |  | TSG | 1 |
| Reported-43 | *COBLL1--SLC38A11* |  |  | 1 |
| Reported-43 | *COPB2--MRPS22* |  |  | 1 |
| Reported-43 | *CPEB3--IDE* | TSG |  | 1 |
| Reported-43 | *CTNNA1--KDM3B* | CG | CG | 1 |
| Reported-43 | *DDX46--SEC24A* |  |  | 1 |
| Reported-43 | *DNAJB1--PRKACA* | CG | OG | 1 |
| Reported-43 | *ECE1--HP1BP3* |  |  | 1 |
| Reported-43 | *FBXW11--STK10* |  |  | 1 |
| Reported-43 | *GIGYF2--EFHD1* |  |  | 1 |
| Reported-43 | *IP6K1--UBA7* |  |  | 1 |
| Reported-43 | *IRAK2--BRK1* |  |  | 1 |
| Reported-43 | *ITCH--ASIP* |  |  | 2 |
| Reported-43 | *ITPK1--MIPOL1* |  |  | 1 |
| Reported-43 | *JUP--ACLY* |  |  | 1 |
| Reported-43 | *KIAA1958--SNX30* |  |  | 1 |
| Reported-43 | *LPIN2--MYOM1* |  |  | 1 |
| Reported-43 | *LRP5--CHKA* | CG |  | 1 |
| Reported-43 | *MED15--COMT* |  |  | 1 |
| Reported-43 | *NFIC--CELF5* |  |  | 1 |
| Reported-43 | *PI4KB--SELENBP1* |  |  | 1 |
| Reported-43 | *PLPP1--SLC38A9* |  |  | 1 |
| Reported-43 | *PPFIA1--RBM4;RBM14-RBM4* |  |  | 1 |
| Reported-43 | *PPFIBP1--STK38L* | CG |  | 1 |
| Reported-43 | *PTPRF--AL451062.3;ST3GAL3* |  |  | 1 |
| Reported-43 | *RNF213--SLC26A11* | CG |  | 1 |
| Reported-43 | *RXRA--WDR5* | CG |  | 1 |
| Reported-43 | *SEC16A--NOTCH1* |  | Both | 1 |
| Reported-43 | *SLC45A2--AMACR;C1QTNF3-AMACR* |  |  | 5 |
| Reported-43 | *ST7--CAPZA2* |  |  | 1 |
| Reported-43 | *TBC1D14--KIAA0232* |  |  | 1 |
| Reported-43 | *TIAM2--SCAF8* |  |  | 1 |
| Reported-43 | *UHRF1BP1L--ANKS1B* |  |  | 1 |
| Reported-43 | *USP14--THOC1* |  |  | 1 |
| Reported-43 | *WDFY2--SERPINE3* |  |  | 1 |
| Reported-43 | *WNK1--ERC1* |  | CG | 1 |
| Reported-actifact-7 | *A2M--PZP* |  |  | 1 |
| Reported-actifact-7 | *ACVR1B--ACVRL1* | TSG |  | 1 |
| Reported-actifact-7 | *AL358113.1;TJP2--PIP5K1B* |  |  | 1 |
| Reported-actifact-7 | *IL1R1--IL1R2* |  |  | 1 |
| Reported-actifact-7 | *MALRD1--PLXDC2* |  |  | 1 |
| Reported-actifact-7 | *PARG--BMS1* |  |  | 1 |
| Reported-actifact-7 | *RNF138--RNF125* |  |  | 2 |

**Table S9**

**Table S9.** The details of 148 genes with novel alternative splicing of 254 Taiwanese HCCs in our cohort.

| Group | Gene name | Gene ID | Gene type |
| --- | --- | --- | --- |
| both_high_bad | *ABCC5* | ENSG00000114770 | protein_coding |
| both_high_bad | *AHCYL2* | ENSG00000158467 | protein_coding |
| both_high_bad | *ANXA6* | ENSG00000197043 | protein_coding |
| both_high_bad | *BRD7* | ENSG00000166164 | protein_coding |
| both_high_bad | *C17orf75* | ENSG00000108666 | protein_coding |
| both_high_bad | *CCDC121* | ENSG00000176714 | protein_coding |
| both_high_bad | *CCDC88A* | ENSG00000115355 | protein_coding |
| both_high_bad | *CCNL2* | ENSG00000221978 | protein_coding |
| both_high_bad | *CYB5R1* | ENSG00000159348 | protein_coding |
| both_high_bad | *DLEU2* | ENSG00000231607 | lncRNA |
| both_high_bad | *DLG1* | ENSG00000075711 | protein_coding |
| both_high_bad | *DNAJB12* | ENSG00000148719 | protein_coding |
| both_high_bad | *DNM2* | ENSG00000079805 | protein_coding |
| both_high_bad | *EFCAB12* | ENSG00000172771 | protein_coding |
| both_high_bad | *EFCAB13* | ENSG00000178852 | protein_coding |
| both_high_bad | *EIF1* | ENSG00000173812 | protein_coding |
| both_high_bad | *EIF2AK3* | ENSG00000172071 | protein_coding |
| both_high_bad | *EIF5A* | ENSG00000132507 | protein_coding |
| both_high_bad | *FAM20A* | ENSG00000108950 | protein_coding |
| both_high_bad | *FGG* | ENSG00000171557 | protein_coding |
| both_high_bad | *FHIT* | ENSG00000189283 | protein_coding |
| both_high_bad | *FXR2* | ENSG00000129245 | protein_coding |
| both_high_bad | *GNS* | ENSG00000135677 | protein_coding |
| both_high_bad | *HNRNPL* | ENSG00000104824 | protein_coding |
| both_high_bad | *IDI1* | ENSG00000067064 | protein_coding |
| both_high_bad | *INTS8* | ENSG00000164941 | protein_coding |
| both_high_bad | *MAP3K12* | ENSG00000139625 | protein_coding |
| both_high_bad | *MAP4K3* | ENSG00000011566 | protein_coding |
| both_high_bad | *MCM8* | ENSG00000125885 | protein_coding |
| both_high_bad | *MXRA7* | ENSG00000182534 | protein_coding |
| both_high_bad | *N4BP2L1* | ENSG00000139597 | protein_coding |
| both_high_bad | *NABP1* | ENSG00000173559 | protein_coding |
| both_high_bad | *NBN* | ENSG00000104320 | protein_coding |
| both_high_bad | *NDUFA9* | ENSG00000139180 | protein_coding |
| both_high_bad | *NUP50-DT* | ENSG00000226328 | lncRNA |
| both_high_bad | *PIGH* | ENSG00000100564 | protein_coding |
| both_high_bad | *PITPNM1* | ENSG00000110697 | protein_coding |
| both_high_bad | *PPP1R13L* | ENSG00000104881 | protein_coding |
| both_high_bad | *PWWP3A* | ENSG00000160953 | protein_coding |
| both_high_bad | *RND1* | ENSG00000172602 | protein_coding |
| both_high_bad | *RPL22* | ENSG00000116251 | protein_coding |
| both_high_bad | *RRP7A* | ENSG00000189306 | protein_coding |
| both_high_bad | *RSL1D1* | ENSG00000171490 | protein_coding |
| both_high_bad | *SCYL2* | ENSG00000136021 | protein_coding |
| both_high_bad | *SENP8* | ENSG00000166192 | protein_coding |
| both_high_bad | *SLC25A1* | ENSG00000100075 | protein_coding |
| both_high_bad | *SLC39A3* | ENSG00000141873 | protein_coding |
| both_high_bad | *SREBF1* | ENSG00000072310 | protein_coding |
| both_high_bad | *SRP9* | ENSG00000143742 | protein_coding |
| both_high_bad | *SRSF5* | ENSG00000100650 | protein_coding |
| both_high_bad | *STRIP1* | ENSG00000143093 | protein_coding |
| both_high_bad | *SVIP* | ENSG00000198168 | protein_coding |
| both_high_bad | *TC2N* | ENSG00000165929 | protein_coding |
| both_high_bad | *TMEM250* | ENSG00000238227 | protein_coding |
| both_high_bad | *TNFSF4* | ENSG00000117586 | protein_coding |
| both_high_bad | *TP53TG1* | ENSG00000182165 | lncRNA |
| both_high_bad | *TPGS2* | ENSG00000134779 | protein_coding |
| both_high_bad | *TRIM25* | ENSG00000121060 | protein_coding |
| both_high_bad | *UBAC2* | ENSG00000134882 | protein_coding |
| both_high_bad | *UBC* | ENSG00000150991 | protein_coding |
| both_high_bad | *UBE2V1* | ENSG00000244687 | protein_coding |
| both_high_bad | *VPS13A* | ENSG00000197969 | protein_coding |
| both_high_bad | *VPS29* | ENSG00000111237 | protein_coding |
| both_high_bad | *WDR12* | ENSG00000138442 | protein_coding |
| both_high_bad | *ZBED8* | ENSG00000221886 | protein_coding |
| both_high_good | *AFF4* | ENSG00000072364 | protein_coding |
| both_high_good | *AGAP4* | ENSG00000188234 | protein_coding |
| both_high_good | *AHCYL1* | ENSG00000168710 | protein_coding |
| both_high_good | *ALDH5A1* | ENSG00000112294 | protein_coding |
| both_high_good | *ARMC10* | ENSG00000170632 | protein_coding |
| both_high_good | *ARPC5* | ENSG00000162704 | protein_coding |
| both_high_good | *ASMTL* | ENSG00000169093 | protein_coding |
| both_high_good | *ATAD2B* | ENSG00000119778 | protein_coding |
| both_high_good | *ATR* | ENSG00000175054 | protein_coding |
| both_high_good | *BRPF1* | ENSG00000156983 | protein_coding |
| both_high_good | *C1orf109* | ENSG00000116922 | protein_coding |
| both_high_good | *CALCOCO2* | ENSG00000136436 | protein_coding |
| both_high_good | *CCNI* | ENSG00000118816 | protein_coding |
| both_high_good | *CD59* | ENSG00000085063 | protein_coding |
| both_high_good | *CERS2* | ENSG00000143418 | protein_coding |
| both_high_good | *CES1* | ENSG00000198848 | protein_coding |
| both_high_good | *CHERP* | ENSG00000085872 | protein_coding |
| both_high_good | *CLPTM1* | ENSG00000104853 | protein_coding |
| both_high_good | *COA4* | ENSG00000181924 | protein_coding |
| both_high_good | *COL4A2* | ENSG00000134871 | protein_coding |
| both_high_good | *CROCCP2* | ENSG00000215908 | transcribed_unprocessed_pseudogene |
| both_high_good | *CUL4A* | ENSG00000139842 | protein_coding |
| both_high_good | *DAPK1* | ENSG00000196730 | protein_coding |
| both_high_good | *DHRSX* | ENSG00000169084 | protein_coding |
| both_high_good | *DIPK2A* | ENSG00000181744 | protein_coding |
| both_high_good | *DNHD1* | ENSG00000179532 | protein_coding |
| both_high_good | *DSTN* | ENSG00000125868 | protein_coding |
| both_high_good | *DYM* | ENSG00000141627 | protein_coding |
| both_high_good | *EEF2K* | ENSG00000103319 | protein_coding |
| both_high_good | *EPB41L5* | ENSG00000115109 | protein_coding |
| both_high_good | *ETFRF1* | ENSG00000205707 | protein_coding |
| both_high_good | *FOXN3* | ENSG00000053254 | protein_coding |
| both_high_good | *FRMD4B* | ENSG00000114541 | protein_coding |
| both_high_good | *FSTL1* | ENSG00000163430 | protein_coding |
| both_high_good | *GGA1* | ENSG00000100083 | protein_coding |
| both_high_good | *GRINA* | ENSG00000178719 | protein_coding |
| both_high_good | *H3-3A* | ENSG00000163041 | protein_coding |
| both_high_good | *HMGCR* | ENSG00000113161 | protein_coding |
| both_high_good | *HSD3B7* | ENSG00000099377 | protein_coding |
| both_high_good | *HSPA12B* | ENSG00000132622 | protein_coding |
| both_high_good | *ING1* | ENSG00000153487 | protein_coding |
| both_high_good | *IRF3* | ENSG00000126456 | protein_coding |
| both_high_good | *KCMF1* | ENSG00000176407 | protein_coding |
| both_high_good | *L3MBTL1* | ENSG00000185513 | protein_coding |
| both_high_good | *LDHA* | ENSG00000134333 | protein_coding |
| both_high_good | *LETMD1* | ENSG00000050426 | protein_coding |
| both_high_good | *LINC00884* | ENSG00000233058 | lncRNA |
| both_high_good | *LRPAP1* | ENSG00000163956 | protein_coding |
| both_high_good | *MAF* | ENSG00000178573 | protein_coding |
| both_high_good | *MIOS* | ENSG00000164654 | protein_coding |
| both_high_good | *MPST* | ENSG00000128309 | protein_coding |
| both_high_good | *NARF* | ENSG00000141562 | protein_coding |
| both_high_good | *NFIB* | ENSG00000147862 | protein_coding |
| both_high_good | *NOLC1* | ENSG00000166197 | protein_coding |
| both_high_good | *NPIPB14P* | ENSG00000226232 | transcribed_unprocessed_pseudogene |
| both_high_good | *NR2C2* | ENSG00000177463 | protein_coding |
| both_high_good | *OSER1-DT* | ENSG00000223891 | lncRNA |
| both_high_good | *PARK7* | ENSG00000116288 | protein_coding |
| both_high_good | *PDLIM3* | ENSG00000154553 | protein_coding |
| both_high_good | *PHF3* | ENSG00000118482 | protein_coding |
| both_high_good | *PTDSS2* | ENSG00000174915 | protein_coding |
| both_high_good | *RAB7A* | ENSG00000075785 | protein_coding |
| both_high_good | *REPIN1* | ENSG00000214022 | protein_coding |
| both_high_good | *RSRP1* | ENSG00000117616 | protein_coding |
| both_high_good | *SIN3A* | ENSG00000169375 | protein_coding |
| both_high_good | *SMAD1* | ENSG00000170365 | protein_coding |
| both_high_good | *SNX17* | ENSG00000115234 | protein_coding |
| both_high_good | *SPPL2B* | ENSG00000005206 | protein_coding |
| both_high_good | *SSBP1* | ENSG00000106028 | protein_coding |
| both_high_good | *TCP11L2* | ENSG00000166046 | protein_coding |
| both_high_good | *TEF* | ENSG00000167074 | protein_coding |
| both_high_good | *TRAPPC12* | ENSG00000171853 | protein_coding |
| both_high_good | *TTC6* | ENSG00000139865 | protein_coding |
| both_high_good | *UBA1* | ENSG00000130985 | protein_coding |
| both_high_good | *VEZF1* | ENSG00000136451 | protein_coding |
| both_high_good | *ZBTB7A* | ENSG00000178951 | protein_coding |
| both_high_good | *ZMAT1* | ENSG00000166432 | protein_coding |
| both_high_good | *ZMYM2* | ENSG00000121741 | protein_coding |
| both_high_good | *ZNF428* | ENSG00000131116 | protein_coding |
| both_high_good & both_high_bad | *CDK13* | ENSG00000065883 | protein_coding |
| both_high_good & both_high_bad | *CFLAR* | ENSG00000003402 | protein_coding |
| both_high_good & both_high_bad | *EGLN1* | ENSG00000135766 | protein_coding |
| both_high_good & both_high_bad | *ZNF717* | ENSG00000227124 | protein_coding |

**Supplementary results**

**Clinical data**

The demographic data of 254 HCC patients are shown in Table S1. There were 42% HBV infection-related HCC patients, 32% HCV infection-related HCC patients, 11% patients with HCC unrelated to HBV or HCV, 7% with HCC related to both HBV and HCV infection, and 7% with double cancers including HCC and another cancer. The male-to-female ratio was 75:25, and the mean age was 60. The mean ages were 55, 65, 61, 58, and 66 years for the HBV, HCV, HBV+HCV, no-B-nor-C, and double cancer groups, respectively. For Edmondson-Steiner grade, 3 cases (1%), 135 cases (53%), 106 cases (42%), 9 cases (4%), and 1 case were grade I, II, III, IV, and unknown, respectively. Macrovascular invasion was present in 76 (30%) cases. For fibrosis score (METAVIR system), 10 cases (4%), 41 cases (16%), 51 cases (20%), 46 cases (18%), 69 cases (27%), and 37 cases (15%) for score 0, 1, 2, 3, 4, and unknown, respectively. The cirrhosis was present in 157 (62%) cases. For clinical stage, 122 cases (48%), 95 cases (37%), 17 cases (7%), 12 cases (5%), 1 case, 4 cases (2%), 1 case, and 2 cases (1%) were stage I, II, III, IIIA, IIIB, IIIC, IVB and unknown, respectively.

**References**

1. Chang, Y.S., et al., *Cancer carrier screening in the general population using whole-genome sequencing.* Cancer Med, 2022.

2. Chang, Y.S., et al., *Integrated genomic analyses of hepatocellular carcinoma.* Hepatol Int, 2022.

3. Draizen, E.J., et al., *HistoneDB 2.0: a histone database with variants--an integrated resource to explore histones and their variants.* Database (Oxford), 2016. **2016**.

4. Lim, L.J., et al., *Roles and Regulation of Long Noncoding RNAs in Hepatocellular Carcinoma.* Cancer Res, 2019. **79**(20): p. 5131-5139.

5. Rheinbay, E., et al., *Analyses of non-coding somatic drivers in 2,658 cancer whole genomes.* Nature, 2020. **578**(7793): p. 102-111.

6. Karczewski, K.J., et al., *The mutational constraint spectrum quantified from variation in 141,456 humans.* Nature, 2020. **581**(7809): p. 434-443.

7. Gudmundsson, S., et al., *Variant interpretation using population databases: Lessons from gnomAD.* Hum Mutat, 2022. **43**(8): p. 1012-1030.

8. Gaujoux, R. and C. Seoighe, *A flexible R package for nonnegative matrix factorization.* BMC Bioinformatics, 2010. **11**: p. 367.

9. Danecek, P., et al., *Twelve years of SAMtools and BCFtools.* Gigascience, 2021. **10**(2).

10. Tate, J.G., et al., *COSMIC: the Catalogue Of Somatic Mutations In Cancer.* Nucleic Acids Res, 2019. **47**(D1): p. D941-D947.

11. Chang, Y.S., et al., *Molecular Classification of Hepatocellular Carcinoma Using Wnt-Hippo Signaling Pathway-Related Genes.* Cancers (Basel), 2022. **14**(19).

12. Wood, D.E., J. Lu, and B. Langmead, *Improved metagenomic analysis with Kraken 2.* Genome Biol, 2019. **20**(1): p. 257.

13. O'Leary, N.A., et al., *Reference sequence (RefSeq) database at NCBI: current status, taxonomic expansion, and functional annotation.* Nucleic Acids Res, 2016. **44**(D1): p. D733-45.

14. Chang, Y.S., et al., *Metatranscriptomic Analysis of Human Lung Metagenomes from Patients with Lung Cancer.* Genes (Basel), 2021. **12**(9).

15. Chang, Y.S., et al., *Genome-Wide Analysis of Prognostic Alternative Splicing Signature and Splicing Factors in Lung Adenocarcinoma.* Genes (Basel), 2020. **11**(11).

16. Yu, C.H., et al., *ARID1A loss derepresses a group of human endogenous retrovirus-H loci to modulate BRD4-dependent transcription.* Nature Communications, 2022. **13**(1): p. 3501.

17. Aran, D., Z. Hu, and A.J. Butte, *xCell: digitally portraying the tissue cellular heterogeneity landscape.* Genome Biol, 2017. **18**(1): p. 220.

18. Therneau, T.M.E., A. ;Cynthia, C, *survival: Survival Analysis.* 2021.

19. Kassambara, A.K., M. ; Biecek, P.; Fabian, S., *survminer: Drawing Survival Curves using ‘ggplot2’. (2021).* 2021.
